# [Tc(NO)Cl_2_(PPh_3_)_2_(CH_3_CN)] and Its Reactions with 2,2′-Dipyridyl Dichalcogenides

**DOI:** 10.3390/molecules30040793

**Published:** 2025-02-08

**Authors:** Till Erik Sawallisch, Susanne Margot Rupf, Abdullah Abdulkader, Moritz Johannes Ernst, Maximilian Roca Jungfer, Ulrich Abram

**Affiliations:** 1 Institute of Chemistry and Biochemistry, Freie Universität Berlin, Fabeckstr. 34/36, 14195 Berlin, Germanysusianne95@zedat.fu-berlin.de (S.M.R.); abdullah.abdulkader@yahoo.com (A.A.); ernstm96@zedat.fu-berlin.de (M.J.E.); 2 Institute of Organic Chemistry, Ruprecht-Karls Universität Heidelberg, Im Neuenheimer Feld 270, 69120 Heidelberg, Germany

**Keywords:** technetium, nitrosyls, dichalcogenides, 2-Pyridyl chalcogenolates, NMR, EPR, X-ray diffraction

## Abstract

The sparingly soluble technetium(I) complex [Tc^I^(NO)Cl_2_(PPh_3_)_2_(CH_3_CN)] (**1**) slowly dissolves during reactions with 2,2′-dipyridyl ditelluride, (2-pyTe)_2_, 2,2′-dipyridyl diselenide, (2-pySe)_2_, or 2,2′-dipyridyl disulfide, (2-pyS)_2_, under formation of deeply colored solutions. Blue (Te compound) or red solids (Se compound) of the composition [{Tc^I^(NO)Cl_2_(PPh_3_)_2_}_2_{µ_2_-(2-pyE)_2_}], E = Te (**3**), Se (**4**), precipitate from the reaction solutions upon addition of toluene. They represent the first technetium complexes with dichalcogenides. While [{Tc^I^(NO)Cl_2_(PPh_3_)}_2_{µ_2_-(2-pyTe)_2_}] (**3**) is the sole product, a small amount of a second product, [Tc^II^(NO)Cl_2_(PPh_3_)(2-pySe)] (**5**), was obtained from the respective mother solution of the reaction with the diselenide. From the corresponding reaction between **1** and (2-pyS)_2_, the technetium(II) compound, [Tc^II^(NO)Cl_2_(PPh_3_)(2-pyS)] (**6**), could be isolated exclusively. The products were studied by single-crystal X-ray diffraction and spectroscopic methods including ^99^Tc NMR for the technetium(I) products and EPR spectroscopy for the Tc(II) complexes. The experimental results are accompanied by DFT considerations, which help to rationalize the experimental observations.

## 1. Introduction

**Figure 1:**
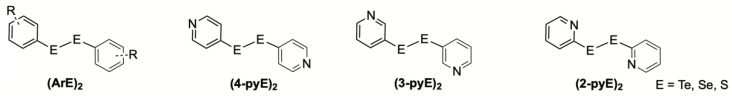
Diaryl dichalcogenides mentioned in this paper and abbreviations used.

The organic chemistry of aryl diselenides and aryl ditellurides is well-established, and, over the past years, such compounds have also found increasing interest as components of metal complexes. Several recent reviews have addressed the progress of the respective research in various fields. This includes fundamental structural chemistry but also applicational aspects of material science, biological chemistry, catalysis, or their use as components in electronic building blocks [1,2,3,4,5,6,7,8,9]. In addition to differently substituted phenyl chalcogenides, (ArE)_2_, bis(pyridyl) diselenides and ditellurides (*n*-pyE)_2_ (Figure 1) in particular have been extensively studied [10,11]. Depending on the position of the pyridine nitrogen atom, such compounds are excellent building blocks for nanomaterials or MOFs, i.e., (4-pyE)_2_ or (3-pyE)_2_ [12,13,14,15,16,17,18,19,20,21], while (2-pyE)_2_ can not only act as ligands in metal complexes but also as precursors for the synthesis of 2-pyridylchalcogenato complexes [10,11].

The pyridylchalcogenolato ligands, which can be obtained by facile reduction of the corresponding (2-pyE)_2_ precursors, show multifaceted coordination chemistry [10,11,22,23,24,25,26], while molecular transition metal complexes with the parent diselenides or ditellurides are relatively rare [27,28,29,30,31,32,33,34]. Only one compound with a ‘group 7 element’ has been reported previously, the Mn(II) complex [Mn{(2-pySe)_2_}Br_2_] [27].

In the present work, we report reactions of the technetium(I) nitrosyl complex [Tc(NO)Cl_2_(PPh_3_)_2_(CH_3_CN)] (**1**) with (2-pyTe)_2_, (2-pySe)_2_ and (2-pyS)_2_, describe the structures of the respective products, and compare the reactivities of the different dichalcogenides.

## 2. Results and Discussion

### 2.1. Structure and Reactivity of the Starting Complex [Tc(NO)Cl_2_(PPh_3_)_2_(CH_3_CN)]

**Scheme 1:**
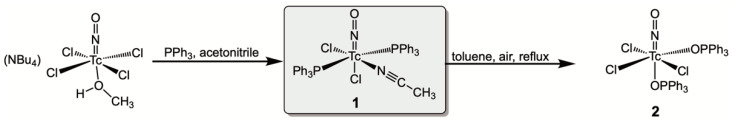
Syntheses of [Tc(NO)Cl_2_(PPh_3_)_2_(CH_3_CN)] [35] and [Tc(NO)Cl_3_(OPPh_3_)_2_].

[Tc(NO)Cl_2_(PPh_3_)_2_(CH_3_CN)] (**1**) is readily prepared from (NBu_4_)[Tc(NO)Cl_4_(MeOH)] with PPh_3_ in acetonitrile (Scheme 1) [35]. An excess of triphenylphosphine acts as a reductant for the technetium(II) starting material, and the poor solubility of the product ensures a high yield and avoids the formation of side products. Although only slightly soluble, compound **1** has been successfully used as a precursor for the synthesis of a considerable number of other nitrosyltechnetium complexes [35,36,37,38,39,40,41]. This is most probably due to the reported gradual decomposition by the loss of the acetonitrile ligand in solution [35], which does commonly not cause problems during ligand-exchange procedures. Likewise, there are also no reports about defined side-products when [Tc(NO)Cl_2_(PPh_3_)_2_(CH_3_CN)] was used as a precursor.

**Figure 2:**
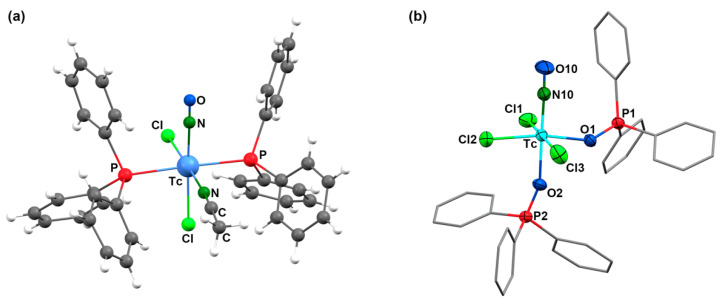
(**a**) Ball and stick representation of the molecular structures of [Tc(NO)Cl_2_(PPh_3_)_2_(CH_3_CN)] (**1**) and (**b**) ellipsoid plot of the structure of [Tc(NO)Cl_3_(OPPh_3_)_2_] (**2**). Thermal ellipsoids represent a 30 percent probability. For color codes, see the atomic labeling scheme.

Surprisingly, the molecular structure of this important precursor of the nitrosyl technetium chemistry has not been elucidated by X-ray diffraction, as the structure of the analogous rhenium(I) compound [Re(NO)Cl_2_(PPh_3_)_2_(CH_3_CN)]. During our (less successful) attempts to conduct ligand exchange, starting from **1** with (2-pyE)_2_ ligands (as in Figure 1) in acetonitrile, we isolated some pale orange-yellow crystals of [Tc(NO)Cl_2_(PPh_3_)_2_(CH_3_CN)], which allowed us to derive basic information about the molecular structure of the complex, such as the composition of the coordination sphere and the arrangement. Unfortunately, the quality of the available single crystals was low; thus, the quality of the derived data was limited. Consequently, they shall not be used here to discuss details of the coordination polyhedron or bond lengths and angles, which are given in the Supplementary Materials and can be inspected there. The coordination positions of the ligands in the environment of technetium and other basic information, however, can doubtlessly be derived and are shown in Figure 2a. The molecular structure of compound **1** is unexceptional, with a distorted octahedral coordination environment around technetium. The nitrosyl ligand is roughly linear, as in all previously studied nitrosyl complexes of technetium [35,36,37,38,39,40,41] and shall, thus, be regarded as a formally NO^+^ ligand. This is in good agreement with the diamagnetism of the compound and the ν_NO_ stretch in the IR spectrum at 1721 cm^−1^ [35]. The acetonitrile ligand binds in an equatorial coordination position *cis* to the nitrosyl ligand. This results in a bonding situation, as found in the similar rhenium complexes [Re(NO)Cl_2_(PR_3_)_2_(CH_3_CN)] (R = Me, cyclohexyl, tolyl) [42,43]. The crystal structure of the analogous triphenylphosphine complex [Re(NO)Cl_2_(PPh_3_)_2_(CH_3_CN)] has not yet been reported.

As previously mentioned, [Tc(NO)Cl_2_(PPh_3_)_2_(CH_3_CN)] (**1**) is a facile starting material for reactions with a variety of ligand systems, and special precautions are not required as long as the incoming ligands are reactive enough. In the case of less reactive ligands, however, the use of an inert atmosphere is strongly recommended since the formation of phosphine oxide complexes cannot be excluded. This has been proven by a prolonged heating of **1** in toluene in air. The sparingly soluble starting compound gradually dissolved in boiling toluene to give a dark purple solution. After a refluxing period of 5 h, an almost clear solution was obtained, from which a purple solid was deposited upon cooling. Single crystals of [Tc(NO)Cl_3_(OPPh_3_)_2_] (**2**) were obtained by slow evaporation of the filtered mother solution. The identity of the crystals with the bulk solid was checked by their IR and EPR spectra. In the technetium(II) complex **2**, the ν_NO_ stretch appears at a clearly higher frequency (1798 cm^−1^) than in compound **1**, as a result of the lower degree of back-donation into the antibonding π* orbitals of the NO^+^ ligand.

[Tc(NO)Cl_3_(OPPh_3_)_2_] crystallizes as toluene solvate in the monoclinic space group *P2_1_/n* with two crystallographically-independent species in the asymmetric unit. The molecular structure of **2** is depicted in Figure 2, and some selected bond lengths and angles are compared to the values of complex **1** in Table 1. One of the triphenylphosphine oxide ligands is coordinated *trans* to the nitrosyl ligand, while the other one is in a plane with the three chlorido ligands. The Tc–O bond lengths are very similar with values between 2.070(4) and 2.086(4) Å, which indicates that the multiple-bonded nitrosyl ligand in the technetium(II) complex **2** does not cause a significant *trans* influence. A similar behavior is observed for the Tc–Cl bonds of the diamagnetic d^6^ complex **1**. The Tc–O–P bonds in complex **2** are bend as is usual for the coordinated triphenylphosphine oxide ligands. The P–O bond lengths (1.490(4) to 1.507(4) Å) reflect the common double-bond character.

**Figure 3:**
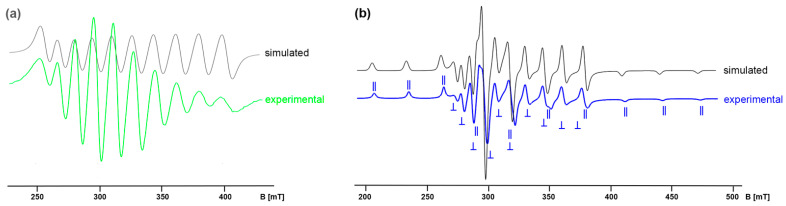
EPR spectra of [Tc(NO)Cl_3_(OPPh_3_)_2_] (**2**) in CHCl_3_: (**a**) at room temperature and (**b**) in frozen solution at *T* = 77 K (‖ indicates the lines of the parallel part; ⊥ indicates the lines of the perpendicular part of the spectrum).

The technetium ion in the Tc(II) complex [Tc(NO)Cl_3_(OPPh_3_)_2_] (**2**) has a d^5^ low-spin configuration with one unpaired electron, which allows the measurement of resolved EPR spectra in liquid and frozen solutions. They show well-resolved hyperfine couplings with the ^99^Tc nucleus. ^99^Tc has a nuclear spin of *I* = 9/2, which causes typical 10-line patterns. Figure 3 shows the spectra of a CHCl_3_ solution of compound **2** at room temperature (a) and at *T* = 77 K (b). The frozen-solution spectrum indicates axial symmetry, resulting in two 10-line patterns each in the parallel and the perpendicular parts of the spectrum.

The experimental spectrum of Figure 3b can be described by the spin Hamiltonian (1), where g_‖_, g_⊥_, A_‖_^Tc^, and A_⊥_^Tc^ are the principal values of the ^99^Tc hyperfine tensor A^Tc^. All other expressions have their usual meaning.

(1) $${\hat{H}}_{sp}=\beta_{e}\left[g_{\|}B_{z}{\hat{S}}_{z}+g_{⊥}\left(B_{x}{\hat{S}}_{x}+B_{y}{\hat{S}}_{y}\right)\right]+A_{\|}^{Tc}{\hat{S}}_{z}{\hat{I}}_{z}+A_{⊥}^{Tc}({\hat{S}}_{x}{\hat{I}}_{x}+{\hat{S}}_{y}{\hat{I}}_{y})$$

Couplings to the ^14^N nucleus of the axial nitrosyl ligand are expectedly small and are not resolved. This is in good agreement with the EPR spectra of all previously studied nitrosyltechnetium(II) compounds. The same holds true for potential couplings to ^31^P nuclei of the OPPh_3_ ligands. They should be very small since no direct Tc–P bonds are established (see Figure 2). The experimental EPR values obtained for [Tc(NO)Cl_3_(OPPh_3_)_2_] are summarized in Table 2 and compared to the corresponding data of a few other Tc(II) nitrosyl or thionitrosyl complexes with phosphine and/or phosphine oxide ligands. In the first column of the Table, the compositions of the equatorial coordination spheres of the complexes are indicated. It should be noted that the MO of the unpaired electron has mainly ‘xy-character’, which means that the EPR parameters are mainly determined by the donor atoms of the equatorial plane.

Compound **2** of the present study is indeed only the second nitrosyl or thionitrosyl technetium(II) complex with a phosphine oxide ligand in its equatorial coordination sphere. Its bromido analog [Tc(NO)Br_3_(OPPh_3_)_2_] has been formed during attempts to oxidize the cyclopentadienyl compound [Tc(NO)(Cp)Br(PPh_3_)] with elemental bromine [44]. A comparison of the EPR parameters, listed in Table 1, indicates a general increase in ^99^Tc hyperfine couplings when phosphine ligands are replaced by phosphine oxides. This can be understood by a lower degree of delocalization of electron density into the ligand orbitals of oxygen donors and is not unusual for axially-symmetric technetium(II) complexes. In a similar way, the influence of the halide ligands on the spectral parameters can be explained, where the transfer of some electron density in the orbitals of bromido or iodido ligands could be proven experimentally, while such effects are much smaller for chlorido ligands [49,50,51].

The formation of the phosphine oxide complex **2** is, in a way, reversible, in that the addition of excess PPh_3_ and HCl to a solution of **2** in acetonitrile results in the reduction and reconstitution of [Tc(NO)Cl_2_(PPh_3_)_2_(CH_3_CN)]. Thus, the detected formation of **2** during ligand exchange procedures starting from **1** does not present a considerable problem, which also applies to reactions of the technetium(I) precursor with 2,2′-dipyridyl dichalcogenides used in the present study.

### 2.2. Reactions of [Tc(NO)Cl_2_(PPh_3_)_2_(CH_3_CN)] (**1**) with 2,2′-Dipyridyl Dichalcogenides

**Scheme 2:**
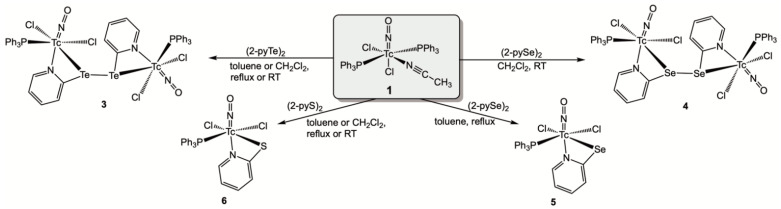
Reactions of [Tc(NO)Cl_2_(PPh_3_)_2_(CH_3_CN)] with 2,2′-dipyridyl dichalcogenides and the structures of the products.

The composition of the complexes formed upon treatment of compound **1** with (2-pyE)_2_ ligands depends on the chalcogen *E* and the reaction conditions applied. Only one product could be isolated with (2-pyTe)_2_, the dark blue, binuclear compound [{Tc(NO)Cl_2_(PPh_3_)}_2_{µ_2_-(2-pyTe)_2_}] (**3**), which is only sparingly soluble and immediately precipitated from CH_2_Cl_2_ (room temperature reaction) or from boiling toluene. A similar product, [{Tc(NO)Cl_2_(PPh_3_)}_2_{µ_2_-(2-pySe)_2_}] (**4**), was obtained during a room-temperature reaction with the corresponding diselenide, while a reduction of (2-pySe)_2_ was observed during prolonged reaction times or at elevated temperatures. The newly formed 2-pyridylselenolate acts as a chelating ligand and forms the technetium(II) complex [Tc(NO)Cl_2_(PPh_3_)(2-pySe)] (**5**). An analogous compound, [Tc(NO)Cl_2_(PPh_3_)(2-pyS)] (**6**), is the sole product of the reaction of **1** with (2-pyS)_2_, irrespective of the reaction conditions. Attempts to isolate a corresponding technetium(I) product with intact disulfide failed, even at –20 °C. Scheme 2 contains a summary of the successful reactions.

**Figure 4:**
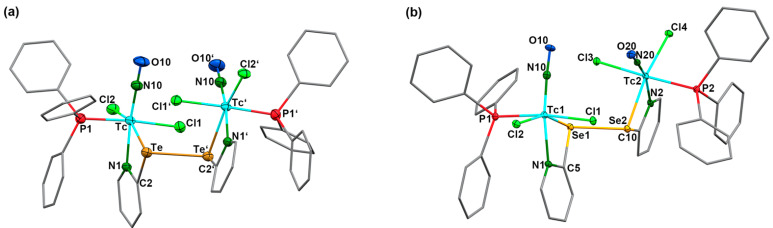
Molecular structures of (**a**) [{Tc(NO)Cl_2_(PPh_3_)}_2_{µ_2_-(2-pyTe)_2_}] (**3**) and (**b**) [{Tc(NO)Cl_2_(PPh_3_)}_2_{µ_2_-(2-pySe)_2_}] (**4**). Thermal ellipsoids represent a 30 percent probability. For color code see the atomic labelling scheme.

The coordination mode of the (2-pyE)_2_ ligands in compound **3** and **4** with two *N,Te* or *N,Se* donor sets bridging two metal ions is without precedence. Most of the hitherto studied metal complexes with 2,2′-dipyridyl dichalcogenides concern the respective disulfides and most of the metal ions are bonded exclusively via the nitrogen atoms of the ligands [21]. Such a bonding mode is also found in a number of complexes with (2-pySe)_2_ [19,27,28,29] or (2-pyTe)_2_ [31,33,34]. Chelate formation involving the chalcogen atoms has only been observed in exceptional cases and commonly results in tridentate *N,E,N* coordination to one metal ion [31,34,52]. Only three compounds are known in which such ligands act as bridges between two metal atoms, and in none of them have two *N,E* chelates been established [31,34,53]. This, however, is the case in the technetium complexes **3** and **4**. The molecular structures of the two compounds are depicted in Figure 4, and selected bond lengths and angles are provided in Table 3.

Complexes **3** and **4** are the first examples of technetium complexes with ditelluride or diselenide ligands. The coordination spheres of the technetium atoms are distorted octahedra. This is mainly due to the limiting bite angles inside the four-membered chelate rings, which cause significant deviations from the ideal 90° and 180° angles inside the respective *N*,*Cl*,*N*,*Te*/*Se* coordination planes. The torsion angles around the chalcogen-chalcogen bonds of the coordinated (2-pyE)_2_ ligands are 85.0° for the ditelluride and 90.6° for the diselenide. The planes of the pyridine rings are twisted by approximately 25°. With regard to the Tc–Tc distances of >5.1 Å in compounds **3** and **4**, no interactions between the technetium atoms can be assumed.

Generally, there are only a few structurally characterized compounds with technetium-tellurium [54] or technetium–selenium bonds [54,55,56,57]. The Tc–Te/Se bond lengths found in **3** and **4** are in the range of those in the [Tc^V^O(PhE)_4_]^−^ anions (E = Te: 2.662 Å, E = Se: 2.473 Å) [54]. Slightly shorter bonds have been found in 5-coordinate technetium(III) complexes of the composition [Tc(PPh_3_)_2_(arylE)_3_] [54], and clearly longer Tc–Se bonds (2.637–2.654 Å) are reported for technetium(I) complexes with selenoether and selenourea ligands [55]. Interestingly, the formation of the *Te*,*N* and *Se*,*N* chelates has a marked influence on the central Te–Te and Se–Se bonds. Their bond lengths increase upon coordination by approximately 0.16 Å (Te complex) and 0.25 Å (Se compound) compared to the values of the uncoordinated dichalcogenides [28,58,59,60]. The suggested weakening of these bonds as a result of the coordination to technetium is discussed as part of the theoretical investigation using density functional theory (DFT) in a later section of this study. It is remarkable that the lengthening of these bonds is more pronounced for the selenium compound and may help to understand the observed formation of a second product, [Tc(NO)Cl_2_(PPh_3_)(2-pySe)] (**5**), during reactions of [Tc(NO)Cl_2_(PPh_3_)_2_(CH_3_CN)] with (2-pySe)_2_, which is observed at harsher reaction conditions. Compound **5** precipitates from the reaction mixture in refluxing toluene as a fairly stable green powder. It is a technetium(II) complex, as could be proven by the measured IR and EPR spectra (vide infra). Solutions of **5** slowly decompose. Thus, we were not yet able to grow single crystals of this compound for X-ray diffraction. The spectroscopic data, however, strongly support a structure in analogy to the corresponding thiolato complex **6**.

**Figure 5:**
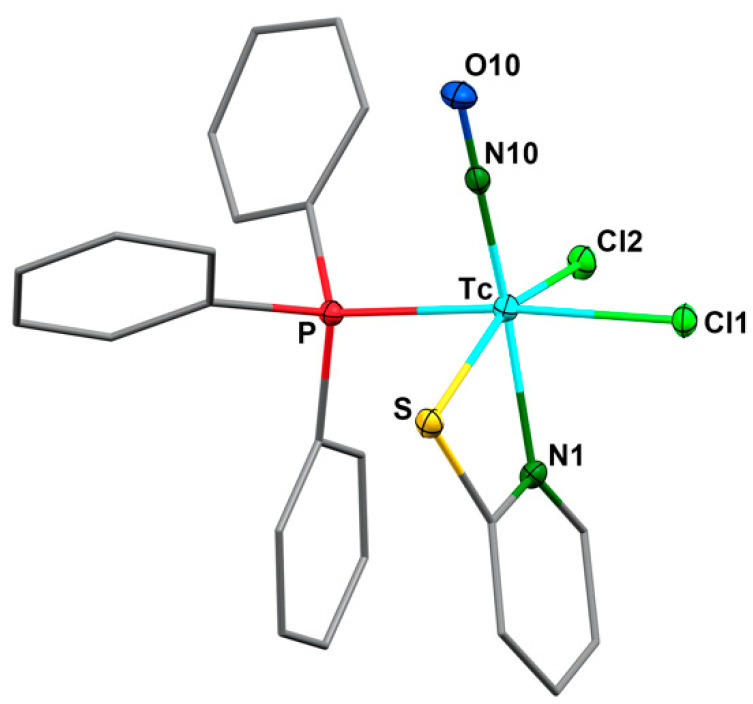
Molecular structure of [Tc(NO)Cl_2_(PPh_3_)(2-pyS)] (**6**). Thermal ellipsoids represent a 30 percent probability. For color code see the atomic labelling scheme.

[Tc(NO)Cl_2_(PPh_3_)(2-pyS)] (**6**) is formed in good yields upon treatment of [Tc(NO)Cl_2_(PPh_3_)_2_(CH_3_CN)] (**1**) with (2-pyS)_2_. Reduction of the disulfide is observed even at room temperature and there was no evidence for the formation of an intermediate with a coordinated disulfide. Green single crystals of compound **6** were obtained from a CH_2_Cl_2_/toluene mixture. The molecular structure of the products is shown in Figure 5; selected bond lengths and angles are summarized in Table 4.

The exclusive formation of a technetium(II) complex during a reaction starting from the Tc(I) precursor [Tc(NO)Cl_2_(PPh_3_)_2_(CH_3_CN)] might be regarded as a result of the ready reduction of the disulfide since simple ligand-exchange reactions of the similar starting material [Tc(NO)Cl_2_(PPh_3_)_2_(CH_3_OH)] with 2-pyridinethiol retains the metals oxidation state, exclusively forming the Tc(I) compounds [Tc(NO)Cl(PPh_3_)_2_(2-pyS)] and [Tc(NO)(PPh_3_)(2-pyS)_2_] [61]. The arrangement of the {2-pyS}^−^ ligand in the technetium(I) products with the nitrogen donor in *trans*-position to NO^+^ is also found in compound **6**. There are also no significant differences in the bond lengths. The linear nitrosyl unit with a Tc–N–O angle of 177.0(2)° is in agreement with the treatment as NO^+^, which is also confirmed by the IR spectroscopic data.

**Figure 6:**
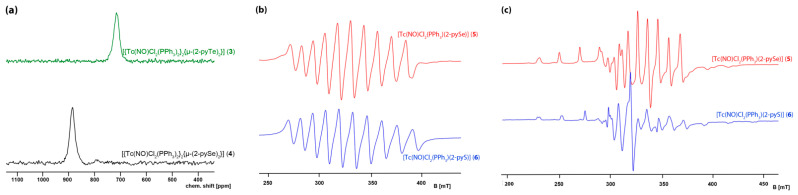
(**a**) ^99^Tc NMR spectra of [{Tc(NO)Cl_2_(PPh_3_)}_2_{µ_2_-(2-pyTe)_2_}] (**3**) and [{Tc(NO)Cl_2_(PPh_3_)}_2_{µ_2_-(2-pySe)_2_}] (**4**), (**b**) room-temperature EPR spectra in CHCl_3_ of [Tc(NO)Cl_2_(PPh_3_)(2-pySe)] (**5**) (g_0_ = 2.0315; a_0_^Tc^ = 119 × 10^−4^ cm^−1^) and [Tc(NO)Cl_2_(PPh_3_)(2-pyS)] (**6**) (g_0_ = 2.0225; a_0_^Tc^ = 126 × 10^−4^ cm^−1^), and (**c**) frozen-solution EPR spectra in CHCl_3_ of **5** (g_‖_ = 2.0850, g_⊥_ = 2.0265; A_‖_^Tc^ = 199 × 10^−4^ cm^−1^, A_⊥_^Tc^ = 86 × 10^−4^ cm^−1^, A_‖_^P^ = 20 × 10^−4^ cm^−1^, A_⊥_^P^ = 18 × 10^−4^ cm^−1^) and **6** (g_‖_ = 2.0650, g_⊥_ = 2.0265; A_‖_^Tc^ = 150 × 10^−4^ cm^−1^, A_⊥_^Tc^ = 86 × 10^−4^ cm^−1^, A_‖_^P^ = 15 × 10^−4^ cm^−1^, A_⊥_^P^ = 9 × 10^−4^ cm^−1^).

The ν_NO_ stretches of Tc(I) complexes **3** and **4** appear between 1722 and 1753 cm^−1^, while those of the Tc(II) products **5** and **6** are found at 1778 and 1782 cm^−1^. The differences are due to the larger degree of backdonation into antibonding ligand orbitals in the technetium(I) complexes with a d^6^ electronic configuration. The diamagnetism of the Tc(I) compounds allows the recording of NMR spectra including the ^99^Tc resonances. ^99^Tc is a remarkable NMR nucleus with a nuclear spin of I = 9/2 and a high relative molar receptivity *versus* ^1^H of approximately 0.4 [62]. This allows for the detection of small differences in the coordination sphere of diamagnetic technetium complexes. A significant drawback is the strong influence of the molecular symmetry on the linewidth of the ^99^Tc NMR signals due to the large quadrupole moment of this nucleus, which results in line widths between some 100 Hz and several kHz. A partial compensation for this inconvenience is given by the extremely large spectral range in which ^99^Tc NMR signals appear. Figure 6a illustrates the situation for the complexes **3** and **4**. Although they are only sparingly soluble, they are chemically very similar, and, as their ^99^Tc NMR line widths are in the range of 2000 Hz, they can readily be recorded with a sufficient signal/noise ratio. They appear at clearly different chemical shifts. The latter fact allows the use of this method to monitor chemical reactions between diamagnetic species and helps to predict the composition of the contributing species. Suitable ^31^P NMR signals could not be resolved for compounds **3** and **4**, which is a frequent feature for compounds with Tc–P bonds and commonly explained by a strong line-broadening due to scalar couplings between ^31^P and the large quadrupole moment of ^99^Tc [46].

In contrast, compounds **5** and **6** are paramagnetic technetium(II) compounds with a d^5^ ‘low-spin’ configuration. The resulting S = ½ system allows for the detection of well-resolved solution EPR spectra at ambient temperatures. They are compared in Figure 6. The expected 10-line pattern due to couplings of the unpaired electron with the nuclear spin of ^99^Tc (I = 9/2) is clearly seen in their liquid solution spectra (Figure 6b). Two sets of ten lines are found in frozen solution spectra of the compounds confirming the ‘axial symmetry’ of the spectra, as has been described for compound **2** vide supra, and a similar spin Hamiltonian (2) can be applied for the description of the spectra. Since the coordination spheres of the technetium atoms in **5** and **6** contain phosphine ligands, which establish couplings with the unpaired electron, the corresponding ^31^P superhyperfine interactions must be considered.

(2) $${\hat{H}}_{sp}=\beta_{e}\left[g_{\|}B_{z}{\hat{S}}_{z}+g_{⊥}\left(B_{x}{\hat{S}}_{x}+B_{y}{\hat{S}}_{y}\right)\right]+A_{\|}^{Tc}{\hat{S}}_{z}{\hat{I}}_{z}+A_{⊥}^{Tc}\left({\hat{S}}_{x}{\hat{I}}_{x}+{\hat{S}}_{y}{\hat{I}}_{y}\right)+\sum SA^{P}I^{P}$$

A comparison of the experimental spectra and the derived parameters (see Figure 6) gives a general trend. The ‘replacement’ of a sulfur donor atom in the equatorial coordination sphere by a selenium atom results in a clear decrease of the ^99^Tc couplings, which means that more electron density is transferred to ligand orbitals. DFT calculations on the B3LYP-GD3B/StuttgartRSC(Tc)/StuttgartRLC+STO-3G(Te)/6-31G*(C,N,P,Cl)/6-31G(H) level indicate that the SOMO orbitals in the paramagnetic Tc(II) monomers are indeed spread over technetium, the chalcogen, the nitrosyl ligand, and the organic pyridyl backbone, while the spin density shows a localization of the unpaired electron at technetium with only slight delocalization onto the nitrosyl ligand. The calculated MO of the unpaired electron is in good agreement with the experimental findings and the only small couplings with the ^31^P nuclei of the phosphine ligands in compounds **5** and **6** (Figure 6c). A visualization of the relevant orbitals can be found in the Supplementary Materials.

The conducted experiments, the spectral data and the structural parameters confirm clear differences between the two technetium(I) complexes **3** and **4** and between the technetium(II) complexes **5** and **6**. The selenium compounds represent a kind of link concerning the spectral parameters and the observed reactivity. Although no direct or quantitative information can be derived from the observed bond lengthening of the Te–Te and Se–Se bonds during the formation of compounds **3** and **4**, a double one electron transfer from each Tc(I) ion to the adjacent chalcogen atom in the dichalcogenide can be assumed for the observed bond cleavage and the generation of the {2-pySe}^−^ and {2-pyS}^−^ ligands. To get more insights into the electronic situation of the complexes, we conducted some studies with computational methods.

### 2.3. DFT Calculations

To rationalize the observed reactivity and the discrepancy in the behavior of the different dichalcogenides, density functional theory (DFT) calculations were performed on the B3LYP level in the gas phase. Better thermochemical parameters were derived based on calculations at the B3LYP-GD3BJ level that were additionally corrected for low-energy rotational modes (see Section 3 for details).

On all levels of theory, the relative trend for the oxidation of technetium and the reduction of the dichalcogenide was Te < Se < S, which is in perfect agreement with the experimental observations. On the higher level calculations, including a solvent model and dispersion correction, it becomes evident that the ditelluride technetium(I) complex **3** is an irreversible thermodynamic sink with an energetic preference of ca. 50 kJ/mol, while, for the selenium compounds, the energy difference allows for an equilibrium between monomeric Tc(II) selenolate and dimeric Tc(I) diselenide. These findings are consistent with the observations made for both Tc(I) and Tc(II) compounds depending on the conditions (ΔΔG ≈ 10 kJ/mol). For the disulfide, the dissociation under the internal redox reaction between S and Tc is favored by ca. 40 kJ/mol, which is in full agreement with the formation of the thiolato complex **6** as the sole product during the reactions conducted. The homolytic cleavage of the E–E bonds in the free dichalcogenides is energetically severely disfavored in all cases, indicating that the metal coordination plays a crucial role in the symmetric dissociation. Interestingly, the lowest barrier is even encountered for the dissociation of the Te–Te bond, which agrees with chemical intuition and highlights the contrast to the metal-mediated reaction described herein in both experiment and theory.

The theoretical widening of the E–E bond upon coordination of the corresponding dichalcogenide to technetium is 2.13 Å → 2.56 Å (Δ(S)_DFT_ = 0.43 Å), 2.49 Å → 2.78 Å (Δ(Se)_DFT_ = 0.29 Å), and 2.83 Å → 2.98 Å (Δ(Te)_DFT_ = 0.15 Å), which is in close agreement with the experimentally observed values of Δ(Te)_exp_ = 0.16 Å and Δ(Se)_exp_ = 0.25 Å. Interestingly, this behavior of the present system represents a unique opportunity to verify the influence of the coordination in two different dichalcogenides with the otherwise same general structure. The observed widening of the E–E bond is consistent with a prediction made for the metal-induced reduction of dichalcogenides by the normally not sufficiently reducing, but intrinsically released, triphenylphosphine ligands upon coordination of the dichalcogenide to group 7 or group 10 metals [63,64,65]. As calculated for palladium species, the chalcogen-chalcogen bond is weakened due to a polarization of the chalcogen-chalcogen bond upon coordination, with the metal-coordinated chalcogen atom becoming partially more negative, while the non-coordinated chalcogen atom shows a significantly increased σ-hole, which makes the E–E bond more prone to the nucleophilic attack of the incoming phosphine, reducing agent forming metal chalcogenolato complexes [65]. The corresponding electrostatic potential (ESP) maps of the dichalcogenides and their technetium complexes are provided as Supplementary Materials; however, the effect of the increased σ-hole is masked by a compression of the structures due to increased interaction of the suitably positioned chloride donor atoms on the σ-hole of the chalcogens. Nevertheless, the bond length deviations are strong evidence for the suggested mechanistic concept of ref. [65].

The experimental ^99^Tc NMR data of compounds **3** and **4** have been used to optimize and test a DFT approach for the prediction of corresponding chemical shifts; for details, see a previous report [66]. The experimental and calculated data are in good agreement (for compound **3**: 716 vs. 703 ppm, for compound **4**: 883 vs. 989 ppm), also keeping in mind that simple solvent effects may cause chemical shift differences up to 100 ppm in the large scale of ^99^Tc chemical shifts of several thousand ppm.

**Figure 7:**
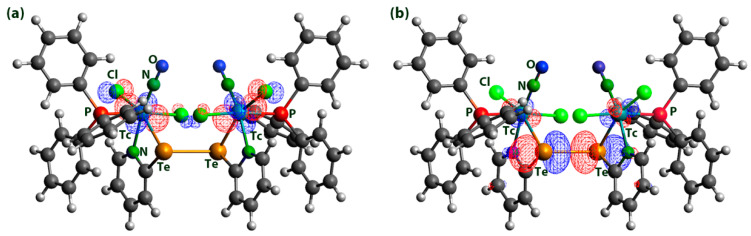
(**a**) HOMO and (**b**) LUMO of [{Tc^I^(NO)Cl_2_(PPh_3_)}_2_{µ_2_-{2-pyTe)_2_] at an isosurface value of 0.05 at the B3LYP-GD3B/StuttgartRSC(Tc)/StuttgartRLC+STO-3G(Te)/6-31G*(C,N,P,Cl)/6-31G(H) level. For color code, see the atomic labelling scheme.

The HOMO and LUMO orbitals in the dimers correspond to a technetium-centered d-orbital and to the anti-bonding σ* orbital of the chalcogen-chalcogen single-bond, respectively, in all cases. The corresponding situation is visualized for the ditelluride complex **3** in Figure 7. Analogous Figures for the other compounds are given as Supplementary Materials.

As the compounds are intensely colored, time-dependent (TD-) DFT calculations were used to simulate the UV-Vis spectra of the compounds and to verify the presence of monomeric Tc(II) for S and Se, while dimeric Tc(I) is observed for Te (see Supplementary Materials). The observed band maxima and spectral shapes are in good agreement with the experimental spectra. The lowest energy absorption band of the Tc(II) compounds shows a bathochromic shift from S to Te, while a hypsochromic shift of the lowest energy visible absorption band is observed from S to Te in the Tc(I) compounds. Thus, [{Tc^I^(NO)Cl_2_(PPh_3_)}_2_{µ_2_-{2-pyTe)_2_] is deep blue as its highest absorption wavelength is lowered into the range of 650 nm compared to [{Tc^I^(NO)Cl_2_(PPh_3_)}_2_{µ_2_-{2-pyE)_2_] (E = S, Se). In comparison, a two-band-pattern with absorptions at ca. 450 nm and 700–750 nm in the UV-Vis spectra of [Tc(NO)Cl_2_(PPh_3_)(2-pyE)] (E = S, Se) leads to the observed green color.

The experimental ^99^Tc NMR data of compounds **3** and **4** have been used to test a DFT approach for the prediction of corresponding chemical shifts; for details, see a previous report [66]. The experimental and calculated data are in good agreement (for compound **3**: 716 vs. 703 ppm, for compound **4**: 883 vs. 989 ppm), also keeping in mind that simple solvent effects may cause chemical shift differences up to 100 ppm in the large scale of ^99^Tc chemical shifts of several thousand ppm.

## 3. Materials and Methods

Unless otherwise stated, reagent-grade solvents and starting materials were used. Solvents were dried and distilled prior to use. Inert conditions were only applied when explicitly mentioned. [Tc(NO)Cl_2_(PPh_3_)_2_(CH_3_CN)] [35], (2-pyTe)_2_ [34], and (2-pySe)_2_ [67] were prepared according to literature procedures.

### 3.1. Radiation Precaution

All synthetic work with technetium was performed in a laboratory approved for the handling of radioactive material. All personnel working in this project were permanently monitored for potential contaminations.

### 3.2. Physical Measurements

IR spectra were measured as KBr pellets on a Shimadzu IR Affinity-1 spectrometer (Shimadzu, Kyoto, Japan). NMR spectra were recorded on JEOL 400 MHz spectrometers (JEOL, Kyoto, Japan). X-Band EPR spectra were recorded in solution with a Magnettech Miniscope MS400 spectrometer (Magnettech, Berlin, Germany) at 300 and 78 K. Simulation and visualization of the EPR spectra were done with the EasySpin toolbox in MatLab (Version: 24.2.0) [68,69].

### 3.3. Syntheses

*[Tc(NO)Cl_3_(OPPh_3_)_2_]* (**2**). [Tc(NO)Cl_2_(PPh_3_)_2_(CH_3_CN)] (**1**) (76 mg, 0.1 mmol) was suspended in toluene (5 mL) and heated under reflux for 5 h in air. The insoluble starting material slowly dissolved and a dark solution was formed. A dark purple solid precipitated upon cooling and purple single crystals formed upon slow evaporation of the residual solution. Yield: 60% (47 mg). IR (KBr, cm^−1^): 3425(m), 3056(w), 2918(w), 1798(vs) ν_NO_, 1589(w), 1437(s), 1319(m), 1175(m) ν_PO_, 1159(m) ν_PO_, 1123(vs) ν_PO_, 1080(s), 1120(m), 1026(m), 997(m), 752(m), 727(vs), 692(s), 623(w), 536(s), 471(w). EPR (RT, CHCl_3_): g_0_ = 2.0291; a_0_^Tc^ = 152 × 10^−4^ cm^−1^. EPR (77 K, CHCl_3_): g_‖_ = 1.9750, g_⊥_ = 2.0320; A_‖_^Tc^ = 273 × 10^−4^ cm^−1^, A_⊥_^Tc^ = 110 × 10^−4^ cm^−1^.
*[{Tc(NO)Cl_2_(PPh_3_)}_2_{µ-(2-pyTe)_2_}]* (**3**). (a) (2-pyTe)_2_ (50 mg, 0.13 mmol) was dissolved in CH_2_Cl_2_ (3 mL) and added to a suspension of [Tc(NO)Cl_2_(PPh_3_)_2_(CH_3_CN)] (**1**) (76 mg, 0.1 mmol) in CH_2_Cl_2_ (3 mL). The mixture was stirred at room temperature for 3 h. A gradual dissolution of the starting material and the formation of a deep-blue solution was observed, from which a blue solid started to precipitate. The solution was filtered, and toluene (2 mL) was added to the filtrate. Slow evaporation of the solvents resulted in the formation of blue single crystals for X-ray diffraction. The crystalline product was identical to the blue powder, which separated from the reaction mixture in the first step, as could be proven by IR, NMR, and UV/vis measurements. Overall yield: 49 mg (74%). (b) (2-pyTe)_2_ (50 mg, 0.13 mmol) was dissolved in 2 mL toluene and added to a suspension of [Tc(NO)Cl_2_(PPh_3_)_2_(CH_3_CN)] (**1**) (76 mg, 0.1 mmol) in toluene (2 mL). The mixture was heated under reflux for 1 h. A deep-blue solid gradually precipitated from the reaction mixture during the reflux period. More product was obtained upon cooling. It was filtered off and recrystallized from CH_2_Cl_2_/toluene. Yield: 37 mg (55%). IR (KBr, cm^−1^): 3433(s), 3037(w), 2968(w), 2920(w), 1753(vs) ν_NO_, 1561(w), 1497(w), 1436(s), 1271(m), 1157(w), 1091(m), 1041(w), 1026(w), 748(m), 696(s), 522(s). ^1^H-NMR (CD_2_Cl_2_, ppm): 8.24 (d, 2H, py), 7.62–7.75 (m, 2H, py), 7.48–7.45 (m, 2H, py), 7.30–7.26 (m, 6H, PPh_3_), 7.09–7.26 (m, 24H, PPh_3_) 6.96–6.93 (m, 2H, py). ^99^Tc-NMR (CD_2_Cl_2_, ppm): 716 (ν_1/2_ = 2200 Hz). UV/Vis (CH_2_Cl_2_, λ_max_, nm, ε): 444, 2863 cm^−1^mol^−1^.
*[{Tc(NO)Cl_2_(PPh_3_)}_2_{µ-(2-pySe)_2_}]* (**4**). (2-pySe)_2_ (40 mg, 0.13 mmol) was dissolved in CH_2_Cl_2_ (3 mL) and added to a suspension of [Tc(NO)Cl_2_(PPh_3_)_2_(CH_3_CN)] (**1**) (76 mg, 0.1 mmol) in CH_2_Cl_2_ (3 mL). The mixture was stirred at room temperature for 3 h. A gradual dissolution of the starting material was observed. The solution was filtered, and toluene (2 mL) was added to the filtrate. Slow evaporation of the solvents resulted in the formation of red-brown single crystals, which were suitable for X-ray diffraction. Yield: 22 mg (35%). More product could be isolated from the mother liquor. This, however, contained significant amounts of the Tc(II) product **5**. Analytical data have been determined for the crystalline product **4**. IR (KBr, cm^−1^): 3428(s), 3049(w), 2968(w), 1722 (vs) ν_NO_, 1562(m), 1497(w), 1436(s), 1414(s) 1186(m), 1117(m), 754(m), 721(m), 694(s), 542(s), 522(m). ^1^H-NMR (CD_2_Cl_2_, ppm): 8.24 (d, 2H, py), 7.62–7.75 (m, 2H, py), 7.48–7.45 (m, 2H,py), 7.30–7.26 (m, 6H, PPh_3_), 7.09–7.26 (m, 24H, PPh_3_) 6.96–6.93 (m, 2H, py). ^99^Tc-NMR (CD_2_Cl_2_, ppm): 885 (ν_1/2_ = 1940 Hz). UV/Vis (CH_2_Cl_2_, λ_max_, nm, ε): 476, 3002 cm^−1^mol^−1^, 768, 2442 cm^−1^mol^−1^.
*[Tc(NO)Cl_2_(PPh_3_)(2-pySe)]* (**5**): [Tc(NO)Cl_2_(PPh_3_)_2_(CH_3_CN)] (76 mg, 0.1 mmol) was suspended in toluene (4 mL) and (2-pySe)_2_ (14 mg, 0.13 mmol) was added dissolved in 4 mL toluene. The mixture was heated under reflux for 90 min, filtered, and allowed to cool to room temperature. A small amount of a green powder was deposited upon slow evaporation of the dark brown solution. Yield: 15 mg (22%). More of compound **5** was contained in the remaining solution, as was confirmed by EPR and IR spectroscopy. This extra amount, however, could not be isolated in an analytically pure form due to impurities of **4** and a second paramagnetic Tc(II) product. Analytical data have been determined for the crystalline product **5**. IR (KBr, cm^−1^): 3426(m), 3053(w), 2922(m), 2852(w), 1778(s) ν_NO_, 1584(s), 1444(vs), 1416(vs), 1266(w), 1192(s), 1119(s), 754(s), 721(s), 688(s), 542(vs). EPR (RT, CHCl_3_): g_0_ = 2.0315; a_0_^Tc^ = 119 × 10^−4^ cm^−1^. EPR (77 K, CHCl_3_): g_‖_ = 2.0850, g_⊥_ = 2.0265; A_‖_^Tc^ = 199 × 10^−4^ cm^−1^, A_⊥_^Tc^ = 86 × 10^−4^ cm^−1^, A_‖_^P^ = 20 × 10^−4^ cm^−1^, A_⊥_^P^ = 18 × 10^−4^ cm^−1^.
*[Tc(NO)Cl_2_(PPh_3_)(2-pyS)]* (**6**): (a) (2-pyS)_2_ (28 mg, 0.13 mmol) was dissolved in CH_2_Cl_2_ (3 mL) and added to a suspension of [Tc(NO)Cl_2_(PPh_3_)_2_(CH_3_CN)] (**1**) (76 mg, 0.1 mmol) in CH_2_Cl_2_ (3 mL). The mixture was stirred at room temperature for 3 h. A gradual dissolution of the starting material and the formation of a green solution was observed. The solution was filtered and toluene (2 mL) was added to the filtrate. Slow evaporation of the solvents resulted in the formation of green single crystals for X-ray diffraction. Yield: 48 mg (85%). (b) The same product was obtained when the reaction was performed in refluxing toluene (1 h). Yield: 43 mg (75%). IR (KBr, cm^−1^): 3068(w), 1759(vs) ν_NO_, 1585(w), 1481(w), 1433(s), 1271(m), 1134(w), 1097(m), 1090(w), 997(w), 764(m), 746(s), 729(s), 694(s), 525(m), 513(m), 497(m), 443(w). EPR (RT, CHCl_3_): g_0_ = 2.0225; a_0_^Tc^ = 126 × 10^−4^ cm^−1^. EPR (77 K, CHCl_3_): g_‖_ = 2.0650, g_⊥_ = 2.0265; A_‖_^Tc^ = 150 × 10^−4^ cm^−1^, A_⊥_^Tc^ = 86 × 10^−4^ cm^−1^, A_‖_^P^ = 15 × 10^−4^ cm^−1^, A_⊥_^P^ = 9 × 10^−4^ cm^−1^. UV/Vis (CH_2_Cl_2_, λ_max_, nm, ε): 462, 2051 cm^−1^mol^−1^, 698, 3650 cm^−1^mol^−1^.

### 3.4. X-Ray Crystallography

The intensities for the X-ray determinations were collected on a Bruker CCD instrument (BRUKER, Billerica, MA, USA) with Mo/Kα radiation. Semi-empirical absorption corrections were carried out by SADABS [70]. Structure solution and refinement were performed with the SHELX programs [71,72] included in the OLEX2 program package [73]. Hydrogen atoms were calculated for idealized positions and treated with the ‘riding model’ option of SHELXL. The solvent mask option of OLEX2 was applied to treat diffuse electron density due to disordered solvents. Details are given in the Supplementary Materials. The representation of molecular structures was conducted using the program Mercury [74].

### 3.5. Computational Chemistry

DFT calculations were performed on the high-performance computing systems of the Freie Universität Berlin ZEDAT (Curta) using the program package GAUSSIAN 16 Rev. A.03 [75]. The gas phase geometry optimizations in vacuum were performed using coordinates derived from the X-ray crystal structures or were modeled with the use of crystal structure fragments using GAUSSVIEW [76], while initial guesses for calculations involving an implicit polarizable continuum model with integral equation formalism (IEF-PCM) for the solvent toluene were derived from the gas phase optimized structures [77]. The gas phase calculations were performed by using the hybrid density functional B3LYP [78,79,80], while the solution phase calculations were empirically corrected for dispersion by Grimme’s D3 method with Becke-Johnson damping [81]. The relativistic small-core basis set Stuttgart RSC 1997, with the respective effective core potential (ECP), was applied to Tc [82,83]. The Stuttgart relativistic large-core basis set augmented by STO-3G polarization functions was applied to S, Se, and Te, together with the respective ECPs [84,85,86,87]. The standard all-electron basis set 6-31G* was applied to C, N, P, and Cl [88,89,90]. The 6-31G basis set was applied for all other atoms [91]. The hybrid functional B3LYP was chosen based on robustness and with regard to a compromise between computational cost and reliable geometry optimization. Although relativistic effects should be small for Tc, as stated in the original benchmarking of the basic constituents of the Stuttgart basis set and ECP, the relativistic small core basis set was proven as a robust yet versatile basis set for Tc in B3LYP (see, e.g., [66,92,93,94,95]). For all other atoms, the choice of basis functions was a compromise between size/accuracy and computational time, and, although the results do not differ much, we preferably use the bigger LANL2DZ for donor atoms directly coordinated to technetium due to its increased accuracy. NMR tensors were calculated for the B3LYP-level optimized gas-phase structures using the B3P86 functional [78,96] combined with the dedicated all-electron NMR basis set x2c-TZVPPall-s for all atoms [97]. Similar approaches have recently been suggested; however, such methods commonly revolved around the exact replication of experimental chemical shifts by exact modeling of solvent, relativistic, and quadrupolar effects with methods of high computational cost and expert knowledge requirements [98,99,100,101,102,103,104], whereas the presented approach of using low-cost functionals combined with low-cost modern basis sets is of much lower computational cost could allow a routine implementation to complement experimental studies by non-experts after an in-depth benchmarking. Such benchmarking is currently ongoing, and a corresponding manuscript is in preparation. All basis sets and ECPs were obtained from the basis set exchange database [105].

The convergence of the optimized geometries was verified by frequency calculations. The absence of negative frequencies characterizes the obtained geometries as energetic minima. Convergence criteria for the frequency calculations: Maximum Force 0.00045; RMS Force 0.00030; Maximum Displacement 0.0018; RMS Displacement 0.0012. Often, the region on the energy hypersurface was very flat for the present systems, so, sometimes, the convergence of single parameters was not tightly adhered to, and structures showing neglectable predicted changes in energy (<2 × 10^−7^ Hartree) were accepted as converged structures if no imaginary frequencies were obtained. The entropic [106] and enthalpic [107] contributions of low-energy modes to the free energy were corrected using the quasi-harmonic approximation of Grimme, as implemented in the freely accessible Python code GoodVibes of Funes-Ardoiz and Paton with a cut-off at 500 cm^−1^ [108].

Further analysis of the obtained wave functions was done using MultiWFN [109]. Visualization of orbitals was done using GAUSSVIEW [76] or Avogadro [110]. A method for the estimation of NMR chemical shifts was taken from ref. [66].

## 4. Conclusions

When exposed to the technetium(I) complex [Tc(NO)Cl_2_(PPh_3_)_2_(CH_3_CN)], 2,2′-Dipyridyl ditelluride, 2,2′-dipyridyl diselenide, and 2,2′-dipyridyl disulfide show clear differences in their reactivity. While reduction of the disulfide and the exclusive formation of the technetium(II) complex [Tc(NO)Cl_2_(PPh_3_)(2-pyS)] is observed, technetium(I) complexes with intact dichalcogenides are isolated during reactions with the corresponding diselenide and ditelluride. Interestingly, the chalcogen-chalcogen bonds in the complexes are widened in the complexes compared with those in the uncoordinated pro-ligands. This effect is more significant for the diselenide, which is in accord with the observed reactivity since this bond is cleaved when the reaction is conducted under elevated temperatures and a Tc(II) compound with the corresponding selenolate is formed. The experimental findings are supported by DFT calculation, which suggests a clear contribution of the back-donation of electron density into antibonding orbitals of the coordinated dichalcogenides, which are located in the corresponding E–E bonds.

Although derived for complexes of the artificial element technetium, the experimental findings might have implications for the explanation of slight differences observed for thiolate/disulfide and selenolate/diselenide couples in biological systems and/or during the development of pharmaceuticals, branches which enter more and more the focus of interest in the current research field [111,112,113,114,115].

## Figures and Tables

**Table 1:** Selected bond lengths (Å) and angles (°) in [Tc(NO)Cl_3_(OPPh_3_)_2_] (**2**) ^(a)^.

Tc–N10	1.753(6) 1.842(6)	N10–O10	1.109(6) 0.932(7)	Tc–Cl1	2.370(2) 2.357(2)	Tc–Cl2	2.335(2) 2.265(3)	Tc–Cl3	2.349(2) 2.366(2)
Tc–O1	2.070(4) 2.082(4)	O1–P1	1.490(4) 1.492(4)	Tc–O2	2.086(4) 2.073(4)	O2–P2	1.487(4) 1.507(4)	Tc–N10–O10	177.7(6) 177.2(10)
Tc–O1–P1	143.9(3) 146.7(3)	Tc–O2–P2	159.8(3) 148.3(2)						

^(a)^ Values for two independent species. For details, including the individual labelling scheme, see the Supplementary Materials.

**Table 2:** EPR parameters of [Tc(NO)Cl_3_(OPPh_3_)_2_] (**3**) together with the values of some other Tc(NO) and Tc(NS) complexes. Coupling constants are given in 10^−4^ cm^−1^.

Compound		g_0_	a_0_^Tc^	g_‖_	g_⊥_	A_‖_^Tc^	A_⊥_^Tc^	A_‖_^P^	Ref.
[Tc(NO)Cl_3_(OPPh_3_)_2_] (**2**)	(**Cl_3_O**) ^(a)^	2.0291	152	1.9750	2.0320	273	110	-	This work
[Tc(NO)Br_3_(OPPh_3_)_2_]	(**Br_3_O**) ^(a)^	2.056	148	2.042	2.059	237	100	-	[44]
[Tc(NO)Cl_3_(PMe_2_Ph)_2_]	(**Cl_2_P_2_**) ^(a)^	2.045	125	2.034	2.053	215	88	19 ^(b)^	[45]
[Tc(NO)Br_3_(PMe_2_Ph)_2_]	(**Br_2_P_2_**) ^(a)^	2.108	111	2.119	2.100	184	79	-	[45]
[Tc(NS)Cl_3_(PPh_3_)_2_]	(**Cl_2_P_2_**) ^(a)^	2.011	164	1.955	2.0455	270	128	-	[46]
[Tc(NS)Cl_3_(PPh_3_)(OPPh_3_)]	(**Cl_3_P**) ^(a)^	2.009	166	1.978	1.999	290	134	-	[46]
[Tc(NS)Cl_3_(PMe_2_Ph)_2_]	(**Cl_2_P_2_**) ^(a)^	2.045	133	2.027	2.038	219	101	19 ^(b)^	[47]
[Tc(NS)Cl_3_(PMe_2_Ph)(OPMe_2_Ph)]	(**Cl_3_P**) ^(a)^	2.032	149	2.027	2.039	237	106	24 ^(c)^	[48]

^(a)^ Composition of the equatorial coordination sphere, ^(b)^ Triplet, ^(c)^ Doublet.

**Table 3:** Selected bond lengths (Å) and angles (°) in [{Tc(NO)Cl_2_(PPh_3_)}_2_{µ_2_-(2-pyTe)_2_}] (**3**) and [{Tc(NO)Cl_2_(PPh_3_)}_2_{µ_2_-(2-pySe)_2_}] (**4**).

Compound **3** ^(a)^							
Tc–N10	1.734(4)	N10–O10	1.184(5)	Tc–Cl1	2.463(1)	Tc–Cl2	2.416(1)
Tc–P1	2.390(1)	Tc–Te	2.6587(5)	Tc–N1	2.181(3)	Te–Te’	2.8319(6)
Tc…Tc’	5.199(2)	Tc–N10–O10	179.5(5)	Te–Tc–N1	70.72(8)	N1–C2–Te	105.8(3)
Compound **4**							
Tc1–N10	1.742(4)	N10–O10	1.197(5)	Tc1–Cl1	2.443(1)	Tc1–Cl2	2.410(1)
Tc1–P1	2.404(1)	Tc1–Se1	2.4730(6)	Tc2–N20	1.743(4)	N20–O20	1.189(5)
Tc2–Cl3	2.446(1)	Tc2–Cl4	2.409(1)	Tc2–P2	2.395(1)	Tc2–Se2	2.4663(6)
Tc2–N2	2.195(4)	Se1–Se2	2.5491(7)	Tc1…Tc2	5.109(2)	Tc1–N10–O10	176.5(4)
Se1–Tc1–N1	69.9(1)	Tc1–N10–O10	176.7(4)	Se2–Tc2–N2	69.8(1)	N1–C5–Se1	108.0(3)
N2–C10–Se2	107.8(4)						

^(a)^ Symmetry operation: x, 1 − y, 3/2 − z.

**Table 4:** Selected bond lengths (Å) and angles (°) in [Tc(NO)Cl_2_(PPh_3_)(2-pyS)] (**6**).

Tc–N10	1.754(2)	N10–O10	1.176(3)	Tc–Cl1	2.4129(7)
Tc–Cl2	2.390(1)	Tc–P	2.4642(7)	Tc–S	2.345(2)
Tc–N1	2.140(2)	Tc–N10–O10	177.0(2)	S–Tc–N1	68.94(7)

## Data Availability

The original contributions presented in this study are included in the article/Supplementary Materials. Further inquiries can be directed to the corresponding author(s).

## Supplementary Materials

The following supporting information can be downloaded at: https://www.mdpi.com/article/10.3390/molecules30040793/s1: Table S1. Crystallographic data and data collection parameters. Figure S1. Ellipsoid representation of the structure of [Tc(NO)Cl_2_(PPh_3_)_2_(CH_3_CN) (**1**). The thermal ellipsoids are set at a 30% probability level. Hydrogen atoms bonding to carbon atoms are omitted for clarity. Table S2. Bond lengths (Å) in [Tc(NO)Cl_2_(PPh_3_)_2_(CH_3_CN) (**1**). Table S3. Bond angles (°) in [Tc(NO)Cl_2_(PPh_3_)_2_(CH_3_CN) (**1**). Figure S2. Ellipsoid representation of the structure of [{Tc(NO)Cl_2_(PPh_3_)}_2_{µ_2_-(2-pyTe)_2_}] (**3**) x CH_2_Cl_2_ x 2 toluene (removed by a solvent mask due to an extended disorder). The thermal ellipsoids are set at a 30% probability level. Hydrogen atoms are omitted for clarity. Table S4. Bond lengths (Å) in [{Tc(NO)Cl_2_(PPh_3_)}(µ_2_-pyTeTepy)] x CH_2_Cl_2_. Table S5. Bond angles (°) in [{Tc(NO)Cl_2_(PPh_3_)}_2_{µ_2_-(2-pyTe)_2_}] (**3**) x CH_2_Cl_2_. Figure S3. Ellipsoid representation of [{Tc(NO)Cl_2_(PPh_3_)}_2_{µ_2_-(2-pySe)_2_}] (**4**) x 1.5 toluene. The thermal ellipsoids are set at a 30% probability level. Hydrogen atoms are omitted for clarity. Table S6. Bond lengths (Å) in [{Tc(NO)Cl_2_(PPh_3_)}_2_{µ_2_-(2-pySe)_2_}] (**4**) x 1.5 toluene. Table S7. Bond angles (°) in [{Tc(NO)Cl_2_(PPh_3_)}_2_{µ_2_-(2-pySe)_2_}] (**4**) x 1.5 toluene. Figure S4. Ellipsoid representation of the complexes contained in [Tc^II^(NO)Cl_2_(PPh_3_)(2-pyS)] (**6**) x 0.5 toluene, also illustrating disorders in the PyS^−^ ligand, two of the phenyl rings and the solvent toluene. The thermal ellipsoids are set at a 30% probability level. Hydrogen atoms are omitted for clarity. Table S8. Bond lengths (Å) in [Tc^II^(NO)Cl_2_(PPh_3_)(2-pyS)] (**6)** x 0.5 toluene. Table S9. Bond angles (°) in [Tc^II^(NO)Cl_2_(PPh_3_)(2-pyS)] (**6**) x 0.5 toluene. Figure S5. IR spectrum (KBr) of [Tc(NO)Cl_3_(OPPh_3_)_2_] (**2**). Figure S6. Room-temperature EPR spectrum of [Tc(NO)Cl_3_(OPPh_3_)_2_] (**2**) in CHCl_3_. Figure S7. Frozen-solution EPR spectrum (T = 78 K) of [Tc(NO)Cl_3_(OPPh_3_)_2_] (**2**) in CHCl_3_. Figure S8. IR spectrum (KBr) of [{Tc^I^(NO)Cl_2_(PPh_3_)}_2_{µ_2_-(2-pyTe)_2_}] (**3**). Figure S9. ^1^H NMR spectra of [{Tc^I^(NO)Cl_2_(PPh_3_)}_2_{µ_2_-(2-pyTe)_2_}] (**3**) in CD_2_Cl_2_. Figure S10. ^99^Tc and ^31^P NMR (not visible) spectra of [{Tc^I^(NO)Cl_2_(PPh_3_)}_2_{µ_2_-(2-pyTe)_2_}] (**3**) in CD_2_Cl_2_. Figure S11. Normalized experimental UV-Vis spectrum of [{Tc^I^(NO)Cl_2_(PPh_3_)}_2_{µ_2_-{2-pyTe)_2_}]. Figure S12. IR spectrum (KBr) of [{Tc^I^(NO)Cl_2_(PPh_3_)}_2_{µ_2_-(2-pySe)_2_}] (**4**). Figure S13. ^1^H NMR spectra of [{Tc^I^(NO)Cl_2_(PPh_3_)}_2_{µ_2_-(2-pySe)_2_}] (**4**) in DMSO-D_6_. Figure S14. ^99^Tc spectrum of [{Tc^I^(NO)Cl_2_(PPh_3_)}_2_{µ_2_-(2-pySe)_2_}] (**4**) DMSO-D_6_. Figure S15. Normalized experimental UV-Vis spectrum of [Tc^II^(NO)Cl_2_(PPh_3_)(2-pySe)]. Figure S16. IR spectrum (KBr) of [Tc(NO)Cl_2_(PPh_3_)(2-pySe)]. Figure S17. Room-temperature X-band EPR spectrum of [Tc(NO)Cl_2_(PPh_3_)(2-pySe)] in CHCl_3_. Figure S18. Frozen-solution (T = 77 K) X-band EPR spectrum of [Tc(NO)Cl_2_(PPh_3_)(2-pySe)] in CHCl_3_. Figure S19. IR spectrum (KBr) of [Tc(NO)Cl_2_(PPh_3_)(2-pyS)]. Figure S20. Room-temperature X-band EPR spectrum of [Tc(NO)Cl_2_(PPh_3_)(2-pyS)] in CHCl_3_. Figure S21. Frozen-solution (T = 77 K) X-band EPR spectrum of [Tc(NO)Cl_2_(PPh_3_)(2-pyS)] in CHCl_3_. Figure S22. Normalized experimental UV-Vis spectrum of [Tc^II^(NO)Cl_2_(PPh_3_)(2-pyS)]. Figure S23. HOMO of [{Tc^I^(NO)Cl_2_(PPh_3_)}_2_{µ_2_-{2-pyS)_2_}] at an isosurface value of 0.05. B3LYP-GD3B/StuttgartRSC(Tc)/StuttgartRLC+STO-3G(S)/6-31G*(C,N,P,Cl)/6-31G(H) level. Figure S24. LUMO of [{Tc^I^(NO)Cl_2_(PPh_3_)}_2_{µ_2_-{2-pyS)_2_}] at an isosurface value of 0.05. B3LYP-GD3B/StuttgartRSC(Tc)/StuttgartRLC+STO-3G(S)/6-31G*(C,N,P,Cl)/6-31G(H) level. Figure S25. Comparison between experimental UV-Vis spectrum of [{Tc^I^(NO)Cl_2_(PPh_3_)}_2_{µ_2_-{2-pyTe)_2_}] and simulated UV-Vis spectra of the (hypothetical) monomeric [Tc^II^(NO)Cl_2_(PPh_3_)(2-pyTe)] or dimeric [{Tc^I^(NO)Cl_2_(PPh_3_)}_2_{µ_2_-{2-pyTe)_2_}]; 50 transitions were respectively considered (indicated by lines). B3LYP-GD3B/StuttgartRSC(Tc)/StuttgartRLC+STO-3G(Te)/6-31G*(C,N,P,Cl)/6-31G(H) level. The spectral signature in the visible part of the spectrum is consistent with the presence of the dimeric Tc(I) instead of the (hypothetical) monomeric Tc(II) compound. Figure S26. Spin density of [Tc(NO)Cl_2_(PPh_3_)(2-pyS)] at an isosurface level of 0.01. B3LYP-GD3B/StuttgartRSC(Tc)/StuttgartRLC+STO-3G(S)/6-31G*(C,N,P,Cl)/6-31G(H) level. Figure S27. SOMO of [Tc(NO)Cl_2_(PPh_3_)(2-pyS)]. B3LYP-GD3B/StuttgartRSC(Tc)/StuttgartRLC+STO-3G(S)/6-31G*(C,N,P,Cl)/6-31G(H) level. Figure S28. LUMO of [Tc(NO)Cl_2_(PPh_3_)(2-pyS)]. B3LYP-GD3B/StuttgartRSC(Tc)/StuttgartRLC+STO-3G(S)/6-31G*(C,N,P,Cl)/6-31G(H) level. Figure S29. Comparison between experimental UV-Vis spectrum of [Tc^II^(NO)Cl_2_(PPh_3_)(2-pyS)] and simulated UV-Vis spectra of the monomeric [Tc^II^(NO)Cl_2_(PPh_3_)(2-pyS)] or (hypothetic) dimeric [{Tc^I^(NO)Cl_2_(PPh_3_)}_2_{µ_2_-{2-pyS)_2_}]; 50 transitions were respectively considered (indicated by lines). B3LYP-GD3B/StuttgartRSC(Tc)/StuttgartRLC+STO-3G(S)/6-31G*(C,N,P,Cl)/6-31G(H) level. The spectral signature in the visible part of the spectrum is consistent with the presence of the monomeric Tc(II) instead of the (hypothetical) dimeric Tc(I) compound. Figure S30. HOMO of [{Tc^I^(NO)Cl_2_(PPh_3_)}_2_{µ_2_-{2-pySe)_2_}] at an isosurface value of 0.05. B3LYP-GD3B/StuttgartRSC(Tc)/StuttgartRLC+STO-3G(Se)/6-31G*(C,N,P,Cl)/6-31G(H) level. Figure S31. LUMO of [{Tc^I^(NO)Cl_2_(PPh_3_)}_2_{µ_2_-{2-pySe)_2_}] at an isosurface value of 0.05. B3LYP-GD3B/StuttgartRSC(Tc)/StuttgartRLC+STO-3G(Se)/6-31G*(C,N,P,Cl)/6-31G(H) level. Figure S32. Comparison between experimental UV-Vis spectrum of [Tc^II^(NO)Cl_2_(PPh_3_)(2-pySe)] and simulated UV-Vis spectra of the monomeric [Tc^II^(NO)Cl_2_(PPh_3_)(2-pySe)] or dimeric [{Tc^I^(NO)Cl_2_(PPh_3_)}_2_{µ_2_-{2-pySe)_2_}]; 50 transitions were respectively considered (indicated by lines). B3LYP-GD3B/StuttgartRSC(Tc)/StuttgartRLC+STO-3G(Se)/6-31G*(C,N,P,Cl)/6-31G(H) level. The spectral signature in the visible part of the spectrum is consistent with the presence of the monomeric Tc(II) instead of the dimeric Tc(I) compound. Figure S33. Spin density of [Tc(NO)Cl_2_(PPh_3_)(2-pySe)] at an isosurface level of 0.01. B3LYP-GD3B/StuttgartRSC(Tc)/StuttgartRLC+STO-3G(Se)/6-31G*(C,N,P,Cl)/6-31G(H) level. Figure S34. SOMO of [Tc(NO)Cl_2_(PPh_3_)(2-pySe)]. B3LYP-GD3B/StuttgartRSC(Tc)/StuttgartRLC+STO-3G(Se)/6-31G*(C,N,P,Cl)/6-31G(H) level. Figure S35. LUMO of [Tc(NO)Cl_2_(PPh_3_)(2-pySe)]. B3LYP-GD3B/StuttgartRSC(Tc)/StuttgartRLC+STO-3G(Se)/6-31G*(C,N,P,Cl)/6-31G(H) level. Figure S36. Simulated UV-Vis spectrum of [Tc(NO)Cl_2_(PPh_3_)(2-pySe)]; 50 transitions were considered. B3LYP-GD3B/StuttgartRSC(Tc)/StuttgartRLC+STO-3G(Se)/6-31G*(C,N,P,Cl)/6-31G(H) level. Figure S37. HOMO of [{Tc^I^(NO)Cl_2_(PPh_3_)}_2_{µ_2_-(2-pyTe)_2_}] at an isosurface value of 0.05. B3LYP-GD3B/StuttgartRSC(Tc)/StuttgartRLC+STO-3G(Te)/6-31G*(C,N,P,Cl)/6-31G(H) level. Figure S38. LUMO of [{Tc^I^(NO)Cl_2_(PPh_3_)}_2_{µ_2_-{2-pyTe)_2_}] at an isosurface value of 0.05. B3LYP-GD3B/StuttgartRSC(Tc)/StuttgartRLC+STO-3G(Te)/6-31G*(C,N,P,Cl)/6-31G(H) level. Figure S39. Simulated UV-Vis spectrum of [{Tc^I^(NO)Cl_2_(PPh_3_)}_2_{µ_2_-(2-pyTe)_2_}]; 50 transitions were considered. B3LYP-GD3B/StuttgartRSC(Tc)/StuttgartRLC+STO-3G(S,Te,Te)/6-31G*(C,N,P,Cl)/6-31G(H) level. Figure S40. Spin density of [Tc(NO)Cl_2_(PPh_3_)(2-pyTe)] at an isosurface level of 0.01. B3LYP-GD3B/StuttgartRSC(Tc)/StuttgartRLC+STO-3G(Te)/6-31G*(C,N,P,Cl)/6-31G(H) level. Figure S41. SOMO of [Tc(NO)Cl_2_(PPh_3_)(2-pyTe)]. B3LYP-GD3B/StuttgartRSC(Tc)/StuttgartRLC+STO-3G(Te)/6-31G*(C,N,P,Cl)/6-31G(H) level. Figure S42. LUMO of [Tc(NO)Cl_2_(PPh_3_)(2-pyTe)]. B3LYP-GD3B/StuttgartRSC(Tc)/StuttgartRLC+STO-3G(Te)/6-31G*(C, N,P,Cl)/6-31G(H) level. Figure S43. Simulated UV-Vis spectrum of [Tc(NO)Cl_2_(PPh_3_)(2-pyTe)]; 50 transitions were considered. B3LYP-GD3B/StuttgartRSC(Tc)/StuttgartRLC+STO-3G(Te)/6-31G*(C, N,P,Cl)/6-31G(H) level. Table S10. Free energies ∆*G* obtained by the DFT calculations at different levels (gas-phase: standard conditions, B3LYP/StuttgartRSC(Tc)/StuttgartRLC+STO-3G(Te)/6-31G*(C,N,P,Cl)/6-31G(H) level; solvent: IEF-PCM for toluene at B3LYP-GD3B/StuttgartRSC(Tc)/StuttgartRLC+STO-3G(Te)/6-31G*(C,N,P,Cl)/6-31G(H) level & solvent with correction for rotational modes). Table S11. Highest occupied molecular orbital (HOMO or singly occupied molecular orbital; SOMO) and highest unoccupied molecular orbital (LUMO) energies and energy differences. Calculations with IEF-PCM for solvent toluene at B3LYP-GD3B/StuttgartRSC(Tc)/StuttgartRLC+STO-3G(Te)/6-31G*(C,N,P,Cl)/6-31G(H) level & solvent with correction for rotational modes). Table S12. ^99^Tc NMR chemical shifts and shielding tensors calculated at B3P86/x2c-TZVPPall-s level based on geometries calculated with IEF-PCM for solvent toluene at B3LYP-GD3B/StuttgartRSC(Tc)/StuttgartRLC+STO-3G(Te)/6-31G*(C,N,P,Cl)/6-31G(H) level & solvent with correction for rotational modes). Table S13. Free energies ∆*G* obtained by the DFT calculations for the dissociation of (2-pyE)_2_ in toluene (IEF-PCM) at B3LYP-GD3B/StuttgartRSC(Tc)/StuttgartRLC+STO-3G(Te)/6-31G*(C,N,P,Cl)/6-31G(H) level. Table S14. Highest occupied molecular orbital (HOMO or singly occupied molecular orbital; SOMO) and highest unoccupied molecular orbital (LUMO) energies and energy differences for ·{2-pyE} and (2-pyE)_2_. Calculations with IEF-PCM for solvent toluene at B3LYP-GD3B/StuttgartRSC(Tc)/StuttgartRLC+STO-3G(Te)/6-31G*(C,N,P,Cl)/6-31G(H) level & solvent with correction for rotational modes). Figure S44. HOMO of (2-pyS)_2_P at an isosurface value of 0.05. B3LYP-GD3B/StuttgartRSC(Tc)/StuttgartRLC+STO-3G(S)/6-31G*(C,N,P,Cl)/6-31G(H) level. Figure S45. LUMO of (2-pyS)_2_ at an isosurface value of 0.05. B3LYP-GD3B/StuttgartRSC(Tc)/StuttgartRLC+STO-3G(S)/6-31G*(C,N,P,Cl)/6-31G(H) level. Figure S46. HOMO of (2-pySe)_2_ at an isosurface value of 0.05. B3LYP-GD3B/StuttgartRSC(Tc)/StuttgartRLC+STO-3G(Se)/6-31G*(C,N,P,Cl)/6-31G(H) level. Figure S47. LUMO of (2-pyTe)_2_ at an isosurface value of 0.05. B3LYP-GD3B/StuttgartRSC(Tc)/StuttgartRLC+STO-3G(Se)/6-31G*(C,N,P,Cl)/6-31G(H) level. Figure S48. HOMO of (2-pyTe)_2_ at an isosurface value of 0.05. B3LYPGD3B/StuttgartRSC(Tc)/StuttgartRLC+STO-3G(Te)/6-31G*(C,N,P,Cl)/6-31G(H) level. Figure S49. LUMO of (2-pyTe)_2_ at an isosurface value of 0.05. B3LYP-GD3B/StuttgartRSC(Tc)/StuttgartRLC+STO-3G(Te)/6-31G*(C,N,P,Cl)/6-31G(H) level. Figure S50. SOMO of ·{2-pyS} at an isosurface value of 0.05. U-B3LYP-GD3B/StuttgartRSC(Tc)/StuttgartRLC+STO-3G(S)/6-31G*(C,N,P,Cl)/6-31G(H) level. Figure S51. LUMO of ·{pyS} at an isosurface value of 0.05. U-B3LYP-GD3B/StuttgartRSC(Tc)/StuttgartRLC+STO-3G(S)/6-31G*(C,N,P,Cl)/6-31G(H) level. Figure S52. SOMO of ·{2-pySe} at an isosurface value of 0.05. U-B3LYP-GD3B/StuttgartRSC(Tc)/StuttgartRLC+STO-3G(Se)/6-31G*(C,N,P,Cl)/6-31G(H) level. Figure S53. LUMO of ·{2-pySe} at an isosurface value of 0.05. U-B3LYP-GD3B/StuttgartRSC(Tc)/StuttgartRLC+STO-3G(Se)/6-31G*(C,N,P,Cl)/6-31G(H) level. Figure S54. SOMO of ·{2-pyTe} at an isosurface value of 0.05. U-B3LYP-GD3B/StuttgartRSC(Tc)/StuttgartRLC+STO-3G(Te)/6-31G*(C,N,P,Cl)/6-31G(H) level. Figure S55. LUMO of ·{2-pyTe} at an isosurface value of 0.05. U-B3LYP-GD3B/StuttgartRSC(Tc)/StuttgartRLC+STO-3G(Te)/6-31G*(C,N,P,Cl)/6-31G(H) level. Figure S56. Spin densities of ·{2-pyE} (left to right; E = S, Se, Te) at an isosurface value of 0.01. U-B3LYP-GD3B/StuttgartRSC(Tc)/StuttgartRLC+STO-3G(S)/6-31G*(C,N,P,Cl)/6-31G(H) level. Figure S57. Electrostatic potential (ESP) mapping of (2-pyS)_2_ at an isosurface value of 0.004 (left: transparent mesh to highlight molecular orientation, right: untransparent mesh to highlight the values). Dark blue: corresponds to a surface potential of 8.314 × 10^−2^, while green is 0 and red is −8.314 × 10^−2^. B3LYP-GD3B/StuttgartRSC(Tc)/StuttgartRLC+STO-3G(S)/6-31G*(C,N,P,Cl)/6-31G(H) level. Figure S58. Electrostatic potential (ESP) mapping of (2-pySe)_2_ at an isosurface value of 0.004 (left: transparent mesh to highlight molecular orientation, right: untransparent mesh to highlight the values). Dark blue: corresponds to a surface potential of 8.314 × 10^−2^, while green is 0 and red is -8.314 × 10^−2^. The values are normalized to those of free (2-pyS)_2_. B3LYP-GD3B/StuttgartRSC(Tc)/StuttgartRLC+STO-3G(Se)/6-31G*(C,N,P,Cl)/6-31G(H) level. Figure S59. Electrostatic potential (ESP) mapping of (2-pyTe)_2_ at an isosurface value of 0.004 (left: transparent mesh to highlight molecular orientation, right: untransparent mesh to highlight the values). Dark blue: corresponds to a surface potential of 8.314 × 10^−2^, while green is 0 and red is −8.314 × 10^−2^. The values are normalized to those of free (2-pyS)_2_. B3LYP-GD3B/StuttgartRSC(Tc)/StuttgartRLC+STO-3G(Te)/6-31G*(C,N, P,Cl)/6-31G(H) level. Figure S60. Electrostatic potential (ESP) mapping of [{Tc^I^(NO)Cl_2_(PPh_3_)}_2_{µ_2_-{2-pyS)_2_}] at an isosurface value of 0.004 (left: transparent mesh to highlight molecular orientation, right: untransparent mesh to highlight the values). Dark blue: corresponds to a surface potential of 8.314 × 10^−2^, while green is 0 and red is −8.314 × 10^−2^. B3LYP-GD3B/StuttgartRSC(Tc)/StuttgartRLC+STO-3G(S)/6-31G*(C,N,P,Cl)/6-31G(H) level. The position of the σ-hole *trans* to the pyridyl substituent is occupied by Cl and therefore obscured, while the position of σ-hole opposite to the chalcogen-chalcogen bond is obscured by the bulk of triphenyl phosphine. Figure S61. Electrostatic potential (ESP) mapping of [{Tc^I^(NO)Cl_2_(PPh_3_)}_2_{µ_2_-{2-pySe)_2_}] at an isosurface value of 0.004 (top: transparent mesh to highlight molecular orientation, bottom: untransparent mesh to highlight the values). Dark blue: corresponds to a surface potential of 8.314 × 10^−2^, while green is 0 and red is −8.314 × 10^−2^. The values are normalized to those of free (2-pyS)_2_. B3LYP-GD3B/StuttgartRSC(Tc)/StuttgartRLC+STO-3G(Se)/6-31G*(C,N,P,Cl)/6-31G(H) level. The position of the σ-hole *trans* to the pyridyl substituent is occupied by Cl and therefore obscured, while the position of σ-hole opposite to the chalcogen-chalcogen bond is obscured by the bulk of triphenyl phosphine. Figure S62. Electrostatic potential (ESP) mapping of [{Tc^I^(NO)Cl_2_(PPh_3_)}_2_{µ_2_-(2-pyTe)_2_}] at an isosurface value of 0.004 (left: transparent mesh to highlight molecular orientation, right: untransparent mesh to highlight the values). Dark blue: corresponds to a surface potential of 8.314 × 10^−2^, while green is 0 and red is −8.314 × 10^−2^. The values are normalized to those of free (2-pyS)_2_. B3LYP-GD3B/StuttgartRSC(Tc)/StuttgartRLC+STO-3G(Te)/6-31G*(C,N,P,Cl)/6-31G(H) level. Crystallographic information file (cif). CheckCif File. Structural data file (.xyz) of the structures for the DFT calculations.
