# Supplementary material for: [Tc(NO)Cl2(PPh3)2(CH3CN)] and Its Reactions with 2,2′-Dipyridyl Dichalcogenides
Source: Molecules. 2025 Feb 8;30(4):793. doi: 10.3390/molecules30040793 (PMC11858252; doi:10.3390/molecules30040793)
Supplement: Supplementary file 1 [file molecules-30-00793-s001.zip › molecules-3447112-supplementary/Supplement/Supplementary_Material_Tc_Dichalcogenides_rev.pdf]

Supplementary Materials to the paper entitled:

[Tc(NO)Cl_2(PPh_3)$_2$(CH_3CN)] and Its Reactions with 2,2′-Dipyridyl Dichalcogenides

Till Erik Sawallisch [1], Susanne Margot Rupf [1], Abdullah Abdulkader [1], Moritz Johannes Ernst [1], Maximilian Roca Jungfer [2*], and Ulrich Abram [1*]

[1] Institute of Chemistry and Biochemistry, Freie Universität Berlin, Fabeckstr. 34/36, 14195 Berlin, Germany.

[2] Institute of Organic Chemistry, Ruprecht-Karls Universität Heidelberg, Im Neuenheimer Feld 270, D-69120 Heidelberg, Germany.

Table of content

1. **Crystallographic data** 8

Table S1. Crystallographic data and data collection parameters 8

Figure S1. Ellipsoid representation of the structure of [Tc(NO)Cl_2(PPh_3)$_2$(CH_3CN) (**1**). The thermal ellipsoids are set at a 30% probability level. Hydrogen atoms bonding to carbon atoms are omitted for clarity. 11

Table S2. Bond lengths (Å) in [Tc(NO)Cl_2(PPh_3)$_2$(CH_3CN) (**1**). 11

Table S3. Bond angles (°) in [Tc(NO)Cl_2(PPh_3)$_2$(CH_3CN) (**1**). 12

Figure S2. Ellipsoid representation of the structure of [{Tc(NO)Cl_2(PPh_3)}$_2${μ_2-(2-pyTe)$_2$}] (**3**) x CH_2Cl_2 x 2 toluene (removed by a solvent mask due to an extended disorder). The thermal ellipsoids are set at a 30% probability level. Hydrogen atoms are omitted for clarity. 13

Table S4. Bond lengths (Å) in [{Tc(NO)Cl_2(PPh_3)}(μ_2-pyTeTepy)] x CH_2Cl_2. 13

Table S5. Bond angles (°) in [{Tc(NO)Cl_2(PPh_3)}$_2${μ_2-(2-pyTe)$_2$}] (**3**) x CH_2Cl_2. 14

Figure S3. Ellipsoid representation of [{Tc(NO)Cl_2(PPh_3)}$_2${μ_2-(2-pySe)$_2$}] (**4**) x 1.5 toluene. The thermal ellipsoids are set at a 30% probability level. Hydrogen atoms are omitted for clarity. 15

Table S6. Bond lengths (Å) in [{Tc(NO)Cl_2(PPh_3)}$_2${μ_2-(2-pySe)$_2$}] (**4**) x 1.5 toluene. 15

Table S7. Bond angles (°) in [{Tc(NO)Cl_2(PPh_3)}$_2${μ_2-(2-pySe)$_2$}] (**4**) x 1.5 toluene. 16

Figure S4. Ellipsoid representation of the complexes contained in [Tc^{II}(NO)Cl_2(PPh_3)(2-pyS)] (**6**) x 0.5 toluene, also illustrating disorders in the PyS^- ligand, two of the phenyl rings and the solvent toluene. The thermal ellipsoids are set at a 30% probability level. Hydrogen atoms are omitted for clarity. 18

Table S8. Bond lengths (Å) in [Tc^{II}(NO)Cl_2(PPh_3)(2-pyS)] (**6**) x 0.5 toluene. 18

Table S9. Bond angles (°) in [Tc^{II}(NO)Cl_2(PPh_3)(2-pyS)] (**6**) x 0.5 toluene. 19

2. **Selected Spectroscopic Data** 20

Figure S5. IR spectrum (KBr) of [Tc(NO)Cl_3($OPPh_3$)$_2$] (**2**). 20

Figure S6. Room-temperature EPR spectrum of [Tc(NO)Cl_3($OPPh_3$)$_2$] (**2**) in $CHCl_3$. 20

Figure S7. Frozen-solution EPR spectrum (T = 78 K) of [Tc(NO)Cl_3($OPPh_3$)$_2$] (**2**) in $CHCl_3$. 21

Figure S8. IR spectrum (KBr) of [{TcI(NO)Cl_2(PPh_3)}$_2${μ_2-(2-pyTe)$_2$}] (**3**). 21

Figure S9. ^{1}H NMR spectra of [{Tc^I(NO)Cl_2(PPh_3)}$_2${μ_2-(2-pyTe)$_2$}] (**3**) in CD_2Cl_2. 22

Figure S10. ^{99}Tc and ^{31}P NMR (not visible) spectra of [{Tc^I(NO)Cl_2(PPh_3)}$_2${μ_2-(2-pyTe)$_2$}] (**3**) in CD_2Cl_2. 22

Figure S11. . Normalized experimental UV-Vis spectrum of [{Tc^I(NO)Cl_2(PPh_3)}$_2${μ_2-{2-pyTe)$_2$}]. 23

Figure S12. IR spectrum (KBr) of of [{Tc^I(NO)Cl_2(PPh_3)}$_2${μ_2-(2-pySe)$_2$}] (**4**). 23

Figure S13. ^{1}H NMR spectra of of [{Tc^{I}(NO)Cl$_2$(PPh$_3$)}$_2${μ$_2$-(2-pySe)$_2$}] (**4**) in DMSO-D$_6$.. 24

Figure S14. ^{99}Tc spectrum of [{Tc^{I}(NO)Cl$_2$(PPh$_3$)}$_2${μ$_2$-(2-pySe)$_2$}] (**4**) DMSO-D$_6$............... 24

Figure S15. Normalized experimental UV-Vis spectrum of [Tc^{II}(NO)Cl$_2$(PPh$_3$)(2-pySe)]. 25

Figure S16. IR spectrum (KBr) of [Tc(NO)Cl$_2$(PPh$_3$)(PySe)]... 25

Figure S17. Room-temperature X-band EPR spectrum of [Tc(NO)Cl$_2$(PPh$_3$)(PySe)] in CHCl$_3$.......... 26

Figure S18. Frozen-solution (T = 77 K) X-band EPR spectrum of [Tc(NO)Cl$_2$(PPh$_3$)(PySe)] in CHCl$_3$. 26

Figure S19. IR spectrum (KBr) of [Tc(NO)Cl$_2$(PPh$_3$)(PyS)]. 27

Figure S20. Room-temperature X-band EPR spectrum of [Tc(NO)Cl$_2$(PPh$_3$)(PyS)] in CHCl$_3$.......... 27

Figure S21. Frozen-solution (T = 77 K) X-band EPR spectrum of [Tc(NO)Cl$_2$(PPh$_3$)(PyS)] in CHCl$_3$. 28

Figure S22. Normalized experimental UV-Vis spectrum of [Tc^{II}(NO)Cl$_2$(PPh$_3$)(2-pyS)]. 28

3. **Computational Chemistry 29**

Figure S23. HOMO of [{Tc^{I}(NO)Cl$_2$(PPh$_3$)}$_2${μ$_2$-{2-pyS)$_2$}] at an isosurface value of 0.05. B3LYP-GD3B/StuttgartRSC(Tc)/StuttgartRLC+STO-3G(S)/6-31G*(C,N,P,Cl)/6-31G(H) level.......... 29

Figure S24. LUMO of [{Tc^{I}(NO)Cl$_2$(PPh$_3$)}$_2${μ$_2$-{2-pyS)$_2$}] at an isosurface value of 0.05. B3LYP-GD3B/StuttgartRSC(Tc)/StuttgartRLC+STO-3G(S)/6-31G*(C,N,P,Cl)/6-31G(H) level.......... 29

Figure S25. Comparison between experimental UV-Vis spectrum of [{Tc^{I}(NO)Cl$_2$(PPh$_3$)}$_2${μ$_2$-{2-pyTe)$_2$}] and simulated UV-Vis spectra of the (hypothetical) monomeric [Tc^{II}(NO)Cl$_2$(PPh$_3$)(2-pyTe)] or dimeric [{Tc^{I}(NO)Cl$_2$(PPh$_3$)}$_2${μ$_2$-{2-pyTe)$_2$}]; 50 transitions were respectively considered (indicated by lines). B3LYP-GD3B/StuttgartRSC(Tc)/StuttgartRLC+STO-3G(Te)/6-31G*(C,N,P,Cl)/6-31G(H) level. The spectral signature in the visible part of the spectrum is consistent with the presence of the dimeric Tc(I) instead of the (hypothetical) monomeric Tc(II) compound.............. 30

Figure S26. Spin density of [Tc(NO)Cl$_2$(PPh$_3$)(2-pyS)] at an isosurface level of 0.01. B3LYP-GD3B/StuttgartRSC(Tc)/StuttgartRLC+STO-3G(S)/6-31G*(C,N,P,Cl)/6-31G(H) level.......... 30

Figure S27. SOMO of [Tc(NO)Cl$_2$(PPh$_3$)(2-pyS)]. B3LYP-GD3B/StuttgartRSC(Tc)/StuttgartRLC+STO-3G(S)/6-31G*(C,N,P,Cl)/6-31G(H) level. 31

Figure S28. LUMO of [Tc(NO)Cl$_2$(PPh$_3$)(2-pyS)]. B3LYP-GD3B/StuttgartRSC(Tc)/StuttgartRLC+STO-3G(S)/6-31G*(C,N,P,Cl)/6-31G(H) level. 31

Figure S29 Comparison between experimental UV-Vis spectrum of [Tc^{II}(NO)Cl$_2$(PPh$_3$)(2-pyS)] and simulated UV-Vis spectra of the monomeric [Tc^{II}(NO)Cl$_2$(PPh$_3$)(2-pyS)] or (hypothetic) dimeric [{Tc^{I}(NO)Cl$_2$(PPh$_3$)}$_2${μ$_2$-{2-pyS)$_2$}]; 50 transitions were respectively

considered (indicated by lines). B3LYP-GD3B/StuttgartRSC(Tc)/StuttgartRLC+STO-3G(S)/6-31G*(C,N,P,Cl)/6-31G(H) level. The spectral signature in the visible part of the spectrum is consistent with the presence of the monomeric Tc(II) instead of the (hypothetical) dimeric Tc(I) compound. 31

Figure S30. HOMO of [{Tc^{I}(NO)Cl_2(PPh_3)}$_2${μ_2-{2-pySe)$_2$}] at an isosurface value of 0.05. B3LYP-GD3B/StuttgartRSC(Tc)/StuttgartRLC+STO-3G(Se)/6-31G*(C,N,P,Cl)/6-31G(H) level. 32

Figure S31. LUMO of [{Tc^{I}(NO)Cl_2(PPh_3)}$_2${μ_2-{2-pySe)$_2$}] at an isosurface value of 0.05. B3LYP-GD3B/StuttgartRSC(Tc)/StuttgartRLC+STO-3G(Se)/6-31G*(C,N,P,Cl)/6-31G(H) level. 32

Figure S32. Comparison between experimental UV-Vis spectrum of [Tc^{II}(NO)Cl_2(PPh_3)(2-pySe)] and simulated UV-Vis spectra of the monomeric [Tc^{II}(NO)Cl_2(PPh_3)(2-pySe)] or dimeric [{Tc^{I}(NO)Cl_2(PPh_3)}$_2${μ_2-{2-pySe)$_2$}]; 50 transitions were respectively considered (indicated by lines). B3LYP-GD3B/StuttgartRSC(Tc)/StuttgartRLC+STO-3G(Se)/6-31G*(C,N,P,Cl)/6-31G(H) level. The spectral signature in the visible part of the spectrum is consistent with the presence of the monomeric Tc(II) instead of the dimeric Tc(I) compound. 33

Figure S33. Spin density of [Tc(NO)Cl_2(PPh_3)(2-pySe)] at an isosurface level of 0.01. B3LYP-GD3B/StuttgartRSC(Tc)/StuttgartRLC+STO-3G(Se)/6-31G*(C,N,P,Cl)/6-31G(H) level. 33

Figure S34. SOMO of [Tc(NO)Cl_2(PPh_3)(2-pySe)]. B3LYP-GD3B/StuttgartRSC(Tc)/StuttgartRLC+STO-3G(Se)/6-31G*(C,N,P,Cl)/6-31G(H) level. 34

Figure S35. LUMO of [Tc(NO)Cl_2(PPh_3)(2-pySe)]. B3LYP-GD3B/StuttgartRSC(Tc)/StuttgartRLC+STO-3G(Se)/6-31G*(C,N,P,Cl)/6-31G(H) level. 34

Figure S36. Simulated UV-Vis spectrum of [Tc(NO)Cl_2(PPh_3)(2-pySe)]; 50 transitions were considered. B3LYP-GD3B/StuttgartRSC(Tc)/StuttgartRLC+STO-3G(Se)/6-31G*(C,N,P,Cl)/6-31G(H) level. 34

Figure S37. HOMO of [{Tc^{I}(NO)Cl_2(PPh_3)}$_2${μ_2-(2-pyTe)$_2$}] at an isosurface value of 0.05. B3LYP-GD3B/StuttgartRSC(Tc)/StuttgartRLC+STO-3G(Te)/6-31G*(C,N,P,Cl)/6-31G(H) level. 35

Figure S38. LUMO of [{Tc^{I}(NO)Cl_2(PPh_3)}$_2${μ_2-{2-pyTe)$_2$}] at an isosurface value of 0.05. B3LYP-GD3B/StuttgartRSC(Tc)/StuttgartRLC+STO-3G(Te)/6-31G*(C,N,P,Cl)/6-31G(H) level. 35

Figure S39. Simulated UV-Vis spectrum of [{Tc^{I}(NO)Cl_2(PPh_3)}$_2${μ_2-(2-pyTe)$_2$}]; 50 transitions were considered. B3LYP-GD3B/StuttgartRSC(Tc)/StuttgartRLC+STO-3G(S,Te,Te)/6-31G*(C,N,P,Cl)/6-31G(H) level. 36

Figure S40. Spin density of [Tc(NO)Cl_2(PPh_3)(2-pyTe)] at an isosurface level of 0.01. B3LYP-GD3B/StuttgartRSC(Tc)/StuttgartRLC+STO-3G(Te)/6-31G*(C,N,P,Cl)/6-31G(H) level. 36

Figure S41. SOMO of [Tc(NO)Cl_2(PPh_3)(2-pyTe)]. B3LYP-GD3B/StuttgartRSC(Tc)/StuttgartRLC+STO-3G(Te)/6-31G*(C,N,P,Cl)/6-31G(H) level. .. 37

Figure S42. LUMO of [Tc(NO)Cl_2(PPh_3)(2-pyTe)]. B3LYP-GD3B/StuttgartRSC(Tc)/StuttgartRLC+STO-3G(Te)/6-31G*(C,N,P,Cl)/6-31G(H) level. .. 37

Figure S43. Simulated UV-Vis spectrum of [Tc(NO)Cl_2(PPh_3)(2-pyTe)]; 50 transitions were considered. B3LYP-GD3B/StuttgartRSC(Tc)/StuttgartRLC+STO-3G(Te)/6-31G*(C,N,P,Cl)/6-31G(H) level. 37

Table S10. Free energies Δ*G* obtained by the DFT calculations at different levels (gas-phase: standard conditions, B3LYP/StuttgartRSC(Tc)/StuttgartRLC+STO-3G(Te)/6-31G*(C,N,P,Cl)/6-31G(H) level; solvent: IEF-PCM for toluene at B3LYP-GD3B/StuttgartRSC(Tc)/StuttgartRLC+STO-3G(Te)/6-31G*(C,N,P,Cl)/6-31G(H) level & solvent with correction for rotational modes). 38

Table S11. Highest occupied molecular orbital (HOMO or singly occupied molecular orbital; SOMO) and highest unoccupied molecular orbital (LUMO) energies and energy differences. Calculations with IEF-PCM for solvent toluene at B3LYP-GD3B/StuttgartRSC(Tc)/StuttgartRLC+STO-3G(Te)/6-31G*(C,N,P,Cl)/6-31G(H) level & solvent with correction for rotational modes). 38

Table S12. ^{99}Tc NMR chemical shifts and shielding tensors calculated at B3P86/x2c-TZVPPall-s level based on geometries calculated with IEF-PCM for solvent toluene at B3LYP-GD3B/StuttgartRSC(Tc)/StuttgartRLC+STO-3G(Te)/6-31G*(C,N,P,Cl)/6-31G(H) level & solvent with correction for rotational modes). 38

Table S13. Free energies Δ*G* obtained by the DFT calculations for the dissociation of (2-pyE)$_2$ in toluene (IEF-PCM) at B3LYP-GD3B/StuttgartRSC(Tc)/StuttgartRLC+STO-3G(Te)/6-31G*(C,N,P,Cl)/6-31G(H) level. 39

Table S14. Highest occupied molecular orbital (HOMO or singly occupied molecular orbital; SOMO) and highest unoccupied molecular orbital (LUMO) energies and energy differences for ·{2-pyE} and (2-pyE)$_2$. Calculations with IEF-PCM for solvent toluene at B3LYP-GD3B/StuttgartRSC(Tc)/StuttgartRLC+STO-3G(Te)/6-31G*(C,N,P,Cl)/6-31G(H) level & solvent with correction for rotational modes). 39

Figure S44. HOMO of (2-pyS)$_2$P at an isosurface value of 0.05. B3LYP-GD3B/StuttgartRSC(Tc)/StuttgartRLC+STO-3G(S)/6-31G*(C,N,P,Cl)/6-31G(H) level. 39

Figure S45. LUMO of (2-pyS)$_2$ at an isosurface value of 0.05. B3LYP-GD3B/StuttgartRSC(Tc)/StuttgartRLC+STO-3G(S)/6-31G*(C,N,P,Cl)/6-31G(H) level. 40

Figure S46. HOMO of (2-pySe)$_2$ at an isosurface value of 0.05. B3LYP-GD3B/StuttgartRSC(Tc)/StuttgartRLC+STO-3G(Se)/6-31G*(C,N,P,Cl)/6-31G(H) level.... 40

Figure S47. LUMO of (2-pyTe)$_2$ at an isosurface value of 0.05. B3LYP-GD3B/StuttgartRSC(Tc)/StuttgartRLC+STO-3G(Se)/6-31G*(C,N,P,Cl)/6-31G(H) level.... 40

Figure S48. HOMO of (2-pyTe)$_2$ at an isosurface value of 0.05. B3LYP-GD3B/StuttgartRSC(Tc)/StuttgartRLC+STO-3G(Te)/6-31G*(C,N,P,Cl)/6-31G(H) level. .. 41

Figure S49. LUMO of $(2\text{-pyTe})_2$ at an isosurface value of 0.05. B3LYP-GD3B/StuttgartRSC(Tc)/StuttgartRLC+STO-3G(Te)/6-31G*(C,N,P,Cl)/6-31G(H) level. .. 41

Figure S50. SOMO of ·{2-pyS} at an isosurface value of 0.05. U-B3LYP-GD3B/StuttgartRSC(Tc)/StuttgartRLC+STO-3G(S)/6-31G*(C,N,P,Cl)/6-31G(H) level. 41

Figure S51. LUMO of ·{pyS} at an isosurface value of 0.05. U-B3LYP-GD3B/StuttgartRSC(Tc)/StuttgartRLC+STO-3G(S)/6-31G*(C,N,P,Cl)/6-31G(H) level. 42

Figure S52. SOMO of ·{2-pySe} at an isosurface value of 0.05. U-B3LYP-GD3B/StuttgartRSC(Tc)/StuttgartRLC+STO-3G(Se)/6-31G*(C,N,P,Cl)/6-31G(H) level.... 42

Figure S53. LUMO of ·{2-pySe} at an isosurface value of 0.05. U-B3LYP-GD3B/StuttgartRSC(Tc)/StuttgartRLC+STO-3G(Se)/6-31G*(C,N,P,Cl)/6-31G(H) level.... 42

Figure S54. SOMO of ·{2-pyTe} at an isosurface value of 0.05. U-B3LYP-GD3B/StuttgartRSC(Tc)/StuttgartRLC+STO-3G(Te)/6-31G*(C,N,P,Cl)/6-31G(H) level. .. 42

Figure S55. LUMO of ·{2-pyTe} at an isosurface value of 0.05. U-B3LYP-GD3B/StuttgartRSC(Tc)/StuttgartRLC+STO-3G(Te)/6-31G*(C,N,P,Cl)/6-31G(H) level. .. 43

Figure S56. Spin densities of ·{2-pyE} (left to right; E = S, Se, Te) at an isosurface value of 0.01. U-B3LYP-GD3B/StuttgartRSC(Tc)/StuttgartRLC+STO-3G(S)/6-31G*(C,N,P,Cl)/6-31G(H) level. 43

Figure S57. Electrostatic potential (ESP) mapping of $(2\text{-pyS})_2$ at an isosurface value of 0.004 (left: transparent mesh to highlight molecular orientation, right: untransparent mesh to highlight the values). Dark blue: corresponds to a surface potential of $8.314 \cdot 10^{-2}$, while green is 0 and red is $-8.314 \cdot 10^{-2}$. B3LYP-GD3B/StuttgartRSC(Tc)/StuttgartRLC+STO-3G(S)/6-31G*(C,N,P,Cl)/6-31G(H) level. 43

Figure S58. Electrostatic potential (ESP) mapping of $(2\text{-pySe})_2$ at an isosurface value of 0.004 (left: transparent mesh to highlight molecular orientation, right: untransparent mesh to highlight the values). Dark blue: corresponds to a surface potential of $8.314 \cdot 10^{-2}$, while green is 0 and red is $-8.314 \cdot 10^{-2}$. The values are normalized to those of free $(2\text{-pyS})_2$. B3LYP-GD3B/StuttgartRSC(Tc)/StuttgartRLC+STO-3G(Se)/6-31G*(C,N,P,Cl)/6-31G(H) level. 44

Figure S59. Electrostatic potential (ESP) mapping of $(2\text{-pyTe})_2$ at an isosurface value of 0.004 (left: transparent mesh to highlight molecular orientation, right: untransparent mesh to highlight the values). Dark blue: corresponds to a surface potential of $8.314 \cdot 10^{-2}$, while green is 0 and red is $-8.314 \cdot 10^{-2}$. The values are normalized to those of free $(2\text{-pyS})_2$. B3LYP-GD3B/StuttgartRSC(Tc)/StuttgartRLC+STO-3G(Te)/6-31G*(C,N,P,Cl)/6-31G(H) level. 44

Figure S60. Electrostatic potential (ESP) mapping of $[\{Tc^{I}(NO)Cl_2(PPh_3)\}_2\{\mu_2\text{-}\{2\text{-pyS}\}_2\}]$ at an isosurface value of 0.004 (left: transparent mesh to highlight molecular orientation, right: untransparent mesh to highlight the values). Dark blue: corresponds to a surface potential of $8.314 \cdot 10^{-2}$, while green is 0 and red is $-8.314 \cdot 10^{-2}$. B3LYP-GD3B/StuttgartRSC(Tc)/StuttgartRLC+STO-3G(S)/6-31G*(C,N,P,Cl)/6-31G(H) level. The position of the σ-hole *trans* to the pyridyl substituent is occupied by Cl and therefore

obscured, while the position of σ-hole opposite to the chalcogen-chalcogen bond is obscured by the bulk of triphenyl phosphine. 45

Figure S61. Electrostatic potential (ESP) mapping of [{Tc^{I}(NO)Cl_2(PPh_3)}$_2${μ_2-{2-pySe)$_2$}] at an isosurface value of 0.004 (top: transparent mesh to highlight molecular orientation, bottom: untransparent mesh to highlight the values). Dark blue: corresponds to a surface potential of $8.314 \cdot 10^{-2}$, while green is 0 and red is $-8.314 \cdot 10^{-2}$. The values are normalized to those of free (2-pyS)$_2$. B3LYP-GD3B/StuttgartRSC(Tc)/StuttgartRLC+STO-3G(Se)/6-31G*(C,N,P,Cl)/6-31G(H) level. The position of the σ-hole *trans* to the pyridyl substituent is occupied by Cl and therefore obscured, while the position of σ-hole opposite to the chalcogen-chalcogen bond is obscured by the bulk of triphenyl phosphine........ 46

Figure S62. Electrostatic potential (ESP) mapping of [{Tc^{I}(NO)Cl_2(PPh_3)}$_2${μ_2-(2-pyTe)$_2$}] at an isosurface value of 0.004 (left: transparent mesh to highlight molecular orientation, right: untransparent mesh to highlight the values). Dark blue: corresponds to a surface potential of $8.314 \cdot 10^{-2}$, while green is 0 and red is $-8.314 \cdot 10^{-2}$. The values are normalized to those of free (2-pyS)$_2$. B3LYP-GD3B/StuttgartRSC(Tc)/StuttgartRLC+STO-3G(Te)/6-31G*(C,N,P,Cl)/6-31G(H) level. 47

1. Crystallographic data

Table S1. Crystallographic data and data collection parameters.

	$[Tc(NO)Cl_2(PPh_3)_2(CH_3CN)]$ (**1**)	$[Tc(NO)Cl_3(OPPh_3)_2]$ (**2**) x 0.5 toluene
Empirical formula	$C_{38}H_{33}Cl_2N_2OP_2Tc$	$C_{39.5}H_{34}Cl_3NO_3P_2Tc$
Formula weight	764.50	836.97
Temperature/K	296.00	258.00
Crystal system	Monoclinic	monoclinic
Space group	$P2_1/c$	$P2_1/n$
a/Å	12.5196(15)	12.5998(8)
b/Å	14.2744(17)	14.1927(11)
c/Å	19.830(2)	44.238(3)
α/°	90	90
β/°	91.481(4)	97.499(2)
γ/°	90	90
Volume/Å^3	11889(2)	7843.3(10)
Z	4	8
ρ_{calc} / gcm^{-3}	1.433	1.418
μ / mm^{-1}	0.680	0.690
F(000)	1560.0	3408.0
Crystal size / mm^3	0.54 × 0.45 × 0.45	0.2 × 0.05 × 0.05
Radiation	MoKα (λ = 0.71073)	MoKα (λ = 0.71073)
2Θ range for data collection/°	4.328 to 49	4 to 49
Index ranges	-14 ≤ h ≤ 14, -16 ≤ k ≤ 16, -23 ≤ l ≤ 23	-14 ≤ h ≤ 14, -16 ≤ k ≤ 16, -51 ≤ l ≤ 51
Reflections collected	63338	155261
Independent reflections	5873 [R_{int} = 0.0771, R_{sigma} = 0.0341]	13047 [R_{int} = 0.0897, R_{sigma} = 0.0376]
Data/restraints/parameters	5873/0/326	13047/25/769
Goodness-of-fit on F^2	1.173	1.080
Final R indexes [I>=2σ (I)]	R_1 = 0.1108, wR_2 = 0.2195	R_1 = 0.0684, wR_2 = 0.1354
Final R indexes [all data]	R_1 = 0.1468, wR_2 = 0.2607	R_1 = 0.0865, wR_2 = 0.1452
Largest diff. peak/hole / e Å^{-3}	1.40/-0.97	1.64/-1.04
Diffractometer	Bruker APEX-II CCD	Bruker APEX-II CCD
Remarks	-	A solvent mask was calculated and 212 electrons were found in a volume of 1216 A^3 in 1 void per unit cell. This is consistent with the presence of 0.5 toluene per formula unit which account for 200 electrons per unit cell.
CCDC access code	Not deposited	2411997

Table S1. Crystallographic data and data collection parameters (continued)

	[{Tc(NO)Cl_2(PPh_3)}$_2${μ$_2$-(2-pyTe)$_2$}] (**3**) x CH_2Cl_2 x 2 toluene	[{Tc(NO)Cl_2(PPh_3)}$_2${μ$_2$-(2-pySe)$_2$}] (**4**) x 1.5 toluene
Empirical formula	$C_{61}H_{56}Cl_6N_4O_2P_2Tc_2Te_2$	$C_{56.5}H_{50}Cl_4N_4O_2P_2Se_2Tc_2$
Formula weight	1602.93	1374.66
Temperature/K	275.00	107.00
Crystal system	Orthorhombic	Monoclinic
Space group	Ibca	$P2_1/c$
a/Å	15.721(1)	12.9663(4)
b/Å	23.827(2))	28.637(1)
c/Å	33.806(4)	16.0265(6)
α/°	90	90
β/°	90	107.384(1)
γ/°	90	90
Volume/Å^3	12663(2)	5679.0(3)
Z	8	4
ρ_{calc} / gcm^{-3}	1.682	1.608
μ / mm^{-1}	1.689	2.058
F(000)	6304.0	2740.0
Crystal size / mm^3	0.4 × 0.05 × 0.05	0.25 × 0.05 × 0.05
Radiation	MoKα (λ = 0.71073)	MoKα (λ = 0.71073)
2Θ range for data collection/°	4.456 to 52.762	3.836 to 52.776
Index ranges	-19 ≤ h ≤ 18, -29 ≤ k ≤ 29, -42 ≤ l ≤ 42	-16 ≤ h ≤ 15, -35 ≤ k ≤ 35, -20 ≤ l ≤ 20
Reflections collected	71742	94786
Independent reflections	6489 [R_{int} = 0.0538, R_{sigma} = 0.0219]	11622 [R_{int} = 0.0727, R_{sigma} = 0.0350]
Data/restraints/parameters	6489/0/294	11622/0/623
Goodness-of-fit on F^2	1.108	1.176
Final R indexes [I>=2σ (I)]	R_1 = 0.0361, wR_2 = 0.0823	R_1 = 0.0437, wR_2 = 0.0968
Final R indexes [all data]	R_1 = 0.0532, wR_2 = 0.0984	R_1 = 0.0723, wR_2 = 0.1232
Largest diff. peak/hole / e Å^{-3}	0.85/-0.75	0.92/-0.93
Diffractometer	Bruker APEX-II CCD	Bruker APEX-II CCD
Remarks	A solvent mask was calculated and 792 electrons were found in a volume of 3060 A^3 in 2 voids per unit cell. This is consistent with the presence of 2 molecules of toluene per formula unit which account for 800 electrons per unit cell.	A solvent mask was calculated and 96 electrons were found in a volume of 816 A^3 in 3 voids per unit cell. This is consistent with the presence of 0.5 molecules of toluene per formula unit which account for 100 electrons per unit cell.
CCDC access code	2411998	2411999

Table S1. Crystallographic data and data collection parameters (continued)

	[Tc(NO)Cl_2(PPh_3)(2-pyS)] (**6**) x 0.5 toluene
Empirical formula	$C_{26.5}H_{23}Cl_2N_2OPSTc$
Formula weight	617.40
Temperature/K	100
Crystal system	Monoclinic
Space group	$P2_1/n$
a/Å	11.5899(6)
b/Å	15.0081(7)
c/Å	15.6037(9)
α/°	90
β/°	99.722(2)
γ/°	90
Volume/Å^3	2675.2(2)
Z	4
ρ_{calc} / gcm^{-3}	1.533
μ / mm^{-1}	0.898
F(000)	1248.0
Crystal size / mm^3	0.34 × 0.18 × 0.11
Radiation	MoKα (λ = 0.71073)
2Θ range for data collection/°	4.788 to 52.784
Index ranges	-14 ≤ h ≤ 14, -18 ≤ k ≤ 18, -19 ≤ l ≤ 19
Reflections collected	55155
Independent reflections	5474 [R_{int} = 0.0447, R_{sigma} = 0.0196]
Data/restraints/parameters	5474/0/381
Goodness-of-fit on F^2	1.155
Final R indexes [I>=2σ (I)]	R_1 = 0.0317, wR_2 = 0.0651
Final R indexes [all data]	R_1 = 0.0415, wR_2 = 0.0733
Largest diff. peak/hole / e Å^{-3}	0.90/-1.11
Diffractometer	Bruker Apex CCD
Remarks	
CCDC access code	2412000

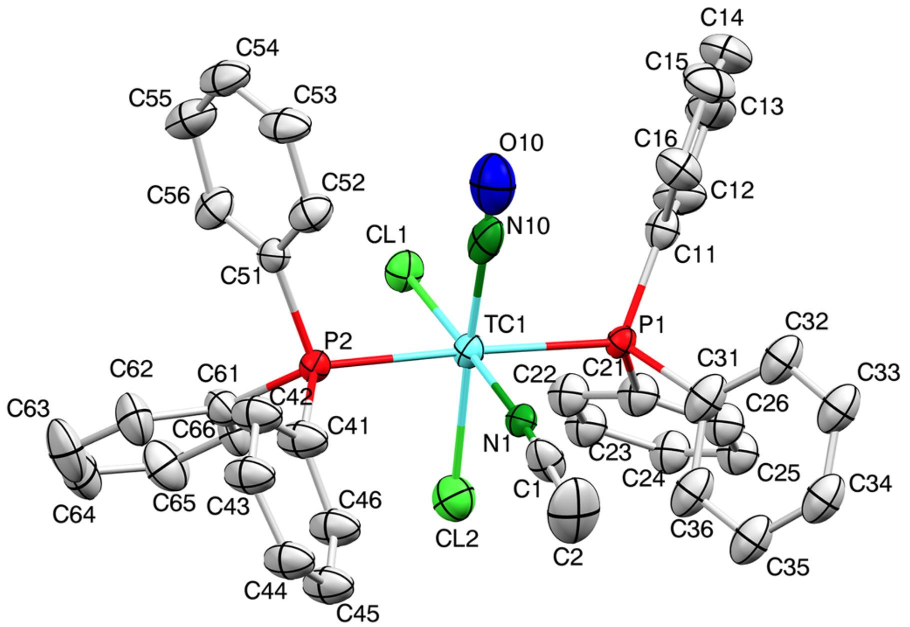

Figure S1. Ellipsoid representation of the structure of [Tc(NO)Cl$_2$(PPh$_3$)$_2$(CH$_3$CN)] (**1**). The thermal ellipsoids are set at a 30% probability level. Hydrogen atoms bonding to carbon atoms are omitted for clarity.

Table S2. Bond lengths (Å) in [Tc(NO)Cl$_2$(PPh$_3$)$_2$(CH$_3$CN)] (**1**).

Tc1	P2	2.458(3)	C66	C65	1.36(2)
Tc1	Cl1	2.432(3)	C36	C31	1.43(2)
Tc1	P1	2.476(3)	C36	C35	1.37(2)
Tc1	Cl2	2.408(5)	C52	C53	1.38(2)
Tc1	N1	2.115(11)	C31	C32	1.38(2)
Tc1	N10	1.869(18)	C23	C24	1.35(2)
P2	C51	1.831(13)	C23	C22	1.399(19)
P2	C41	1.833(15)	C32	C33	1.39(2)
P2	C61	1.828(14)	C16	C15	1.37(2)
P1	C21	1.816(15)	C55	C54	1.35(2)
P1	C11	1.812(15)	C46	C45	1.37(2)
P1	C31	1.813(16)	C53	C54	1.37(2)
N1	C1	1.128(16)	C45	C44	1.34(2)
C51	C56	1.361(18)	C42	C43	1.40(2)
C51	C52	1.402(19)	C2	C1	1.40(2)
C21	C26	1.39(2)	C35	C34	1.34(2)
C21	C22	1.39(2)	C12	C13	1.38(2)
C41	C46	1.39(2)	C34	C33	1.37(2)
C41	C42	1.37(2)	C44	C43	1.39(2)
C56	C55	1.40(2)	C62	C63	1.35(2)
C11	C16	1.39(2)	C64	C65	1.37(3)
C11	C12	1.38(2)	C64	C63	1.38(3)
C61	C66	1.370(19)	C15	C14	1.38(3)
C61	C62	1.42(2)	C13	C14	1.32(3)
C25	C26	1.415(19)	N10	O10	0.915(19)
C25	C24	1.32(2)			

Table S3. Bond angles (°) in [Tc(NO)Cl_2(PPh_3)$_2$(CH_3CN)] (**1**).

P2	Tc1	P1	176.49(12)	C12	C11	P1	123.1(12)
Cl1	Tc1	P2	88.40(11)	C12	C11	C16	116.0(15)
Cl1	Tc1	P1	90.39(11)	C66	C61	P2	123.1(11)
Cl2	Tc1	P2	89.04(13)	C66	C61	C62	116.1(14)
Cl2	Tc1	Cl1	94.89(14)	C62	C61	P2	120.8(12)
Cl2	Tc1	P1	87.78(13)	C24	C25	C26	119.9(15)
N1	Tc1	P2	90.0(3)	C65	C66	C61	122.4(15)
N1	Tc1	Cl1	178.4(3)	C35	C36	C31	121.2(18)
N1	Tc1	P1	91.2(3)	C53	C52	C51	118.8(15)
N1	Tc1	Cl2	85.2(3)	C36	C31	P1	117.3(14)
N10	Tc1	P2	92.0(5)	C32	C31	P1	126.7(15)
N10	Tc1	Cl1	89.3(5)	C32	C31	C36	115.9(15)
N10	Tc1	P1	91.3(5)	C24	C23	C22	120.1(15)
N10	Tc1	Cl2	175.7(6)	C21	C26	C25	119.8(15)
N10	Tc1	N1	90.6(6)	C31	C32	C33	121.7(18)
C51	P2	Tc1	109.6(4)	C15	C16	C11	121.8(16)
C51	P2	C41	105.1(7)	C54	C55	C56	120.8(17)
C41	P2	Tc1	116.2(6)	C45	C46	C41	120.6(15)
C61	P2	Tc1	120.4(5)	C54	C53	C52	122.3(17)
C61	P2	C51	103.0(6)	C44	C45	C46	121.9(16)
C61	P2	C41	100.7(7)	C55	C54	C53	118.5(16)
C21	P1	Tc1	118.5(5)	C41	C42	C43	119.9(15)
C11	P1	Tc1	115.6(4)	C34	C35	C36	120.7(19)
C11	P1	C21	100.9(6)	C25	C24	C23	122.2(15)
C11	P1	C31	107.0(8)	C13	C12	C11	121.3(18)
C31	P1	Tc1	111.9(5)	C21	C22	C23	119.6(14)
C31	P1	C21	101.5(7)	N1	C1	C2	178.0(17)
C1	N1	Tc1	176.7(11)	C35	C34	C33	120.6(17)
C56	C51	P2	120.4(11)	C45	C44	C43	118.6(15)
C56	C51	C52	118.6(13)	C63	C62	C61	121.2(18)
C52	C51	P2	120.5(10)	C65	C64	C63	118.3(17)
C26	C21	P1	121.2(12)	C16	C15	C14	120.1(18)
C26	C21	C22	118.4(13)	C14	C13	C12	122.1(19)
C22	C21	P1	119.8(11)	C66	C65	C64	120.8(18)
C46	C41	P2	117.9(12)	C34	C33	C32	120.0(18)
C42	C41	P2	123.5(13)	C13	C14	C15	118.8(18)
C42	C41	C46	118.4(14)	C44	C43	C42	120.3(15)
C51	C56	C55	121.0(15)	C62	C63	C64	121.0(17)
C16	C11	P1	120.9(11)	O10	N10	Tc1	176(3)

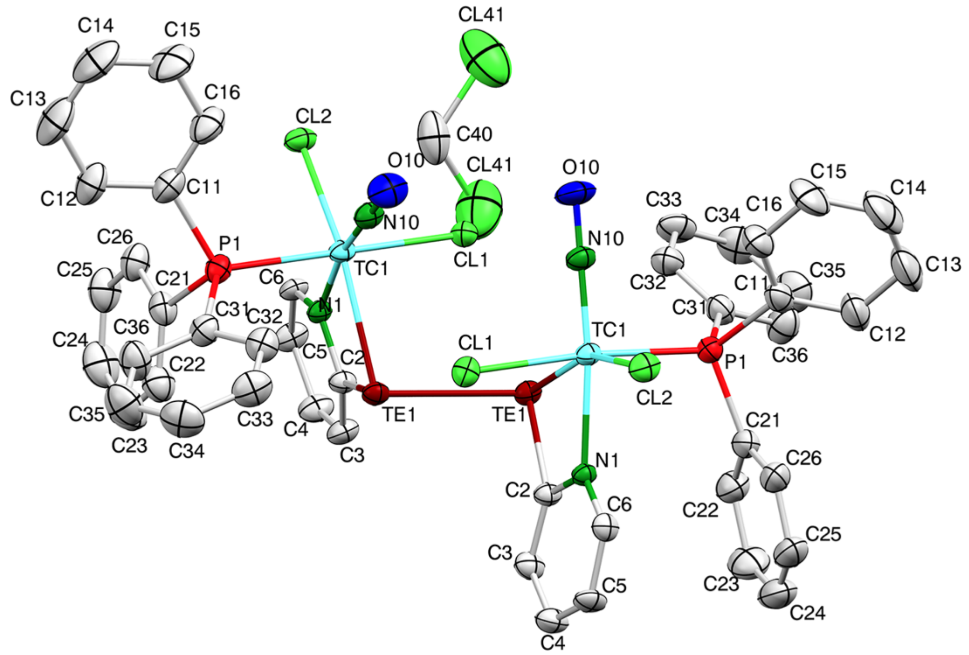

Figure S2. Ellipsoid representation of the structure of [{Tc(NO)Cl$_2$(PPh$_3$)}$_2${μ$_2$-(2-pyTe)$_2$}] (**3**) x CH_2Cl_2 x 2 toluene (removed by a solvent mask due to an extended disorder). The thermal ellipsoids are set at a 30% probability level. Hydrogen atoms are omitted for clarity.

Table S4. Bond lengths (Å) in [{Tc(NO)Cl$_2$(PPh$_3$)}(μ$_2$-pyTeTepy)] x CH_2Cl_2.

Te1	Te1[1]	2.8319(6)	C11	C16	1.368(8)
Te1	Tc1	2.6587(5)	C11	C12	1.398(8)
Te1	C2	2.153(4)	C21	C26	1.394(7)
Tc1	Cl1	2.4633(12)	C21	C22	1.374(8)
Tc1	Cl2	2.4161(11)	C31	C32	1.390(7)
Tc1	P1	2.3895(13)	C31	C36	1.389(7)
Tc1	N1	2.181(3)	C26	C25	1.382(8)
Tc1	N10	1.734(4)	C32	C33	1.389(7)
P1	C11	1.832(5)	C22	C23	1.405(8)
P1	C21	1.819(5)	C16	C15	1.386(8)
P1	C31	1.838(5)	C33	C34	1.386(9)
Cl41	C40	1.720(5)	C36	C35	1.384(8)
N1	C2	1.339(5)	C12	C13	1.389(10)
N1	C6	1.329(5)	C34	C35	1.374(10)
O10	N10	1.184(5)	C25	C24	1.356(10)
C2	C3	1.364(6)	C23	C24	1.368(10)
C6	C5	1.381(6)	C15	C14	1.356(11)
C3	C4	1.390(6)	C14	C13	1.376(12)
C4	C5	1.372(6)			

[1] x,1-y,3/2-z

Table S5. Bond angles (°) in [{Tc(NO)Cl_2(PPh_3)}$_2${μ$_2$-(2-pyTe)$_2$}] (**3**) x CH_2Cl_2.

Tc1	Te1	Te1[1]	100.667(12)	C3	C2	Te1	130.7(3)
C2	Te1	Te1[1]	92.04(11)	N1	C6	C5	121.3(4)
C2	Te1	Tc1	73.67(11)	C2	C3	C4	117.6(4)
Cl1	Tc1	Te1	88.59(3)	C5	C4	C3	119.1(4)
Cl2	Tc1	Te1	159.10(3)	C4	C5	C6	119.7(4)
Cl2	Tc1	Cl1	88.67(4)	C16	C11	P1	120.3(4)
P1	Tc1	Te1	92.31(3)	C16	C11	C12	118.8(5)
P1	Tc1	Cl1	173.95(4)	C12	C11	P1	120.9(5)
P1	Tc1	Cl2	88.37(4)	C26	C21	P1	119.2(4)
N1	Tc1	Te1	70.72(8)	C22	C21	P1	121.2(4)
N1	Tc1	Cl1	82.37(9)	C22	C21	C26	118.6(5)
N1	Tc1	Cl2	88.38(9)	C32	C31	P1	120.6(4)
N1	Tc1	P1	92.28(9)	C36	C31	P1	120.8(4)
N10	Tc1	Te1	100.61(12)	C36	C31	C32	118.5(5)
N10	Tc1	Cl1	94.69(13)	C25	C26	C21	121.6(6)
N10	Tc1	Cl2	100.26(12)	C33	C32	C31	121.3(5)
N10	Tc1	P1	91.03(13)	C21	C22	C23	119.8(6)
N10	Tc1	N1	170.83(15)	C11	C16	C15	121.6(7)
C11	P1	Tc1	116.4(2)	C34	C33	C32	118.8(6)
C11	P1	C31	101.3(2)	C35	C36	C31	120.7(6)
C21	P1	Tc1	108.65(16)	C13	C12	C11	119.3(7)
C21	P1	C11	105.9(2)	C35	C34	C33	120.8(6)
C21	P1	C31	105.7(3)	C24	C25	C26	118.9(6)
C31	P1	Tc1	117.89(16)	C24	C23	C22	119.8(6)
C2	N1	Tc1	109.4(3)	C34	C35	C36	119.9(7)
C6	N1	Tc1	131.7(3)	C14	C15	C16	119.2(8)
C6	N1	C2	118.7(3)	C25	C24	C23	121.5(6)
O10	N10	Tc1	179.5(5)	Cl41[2]	C40	Cl41	113.6(6)
N1	C2	Te1	105.8(3)	C15	C14	C13	120.8(7)
N1	C2	C3	123.6(4)	C14	C13	C12	120.2(8)

[1] x,1-y,3/2-z; [2] 1-x,3/2-y,z

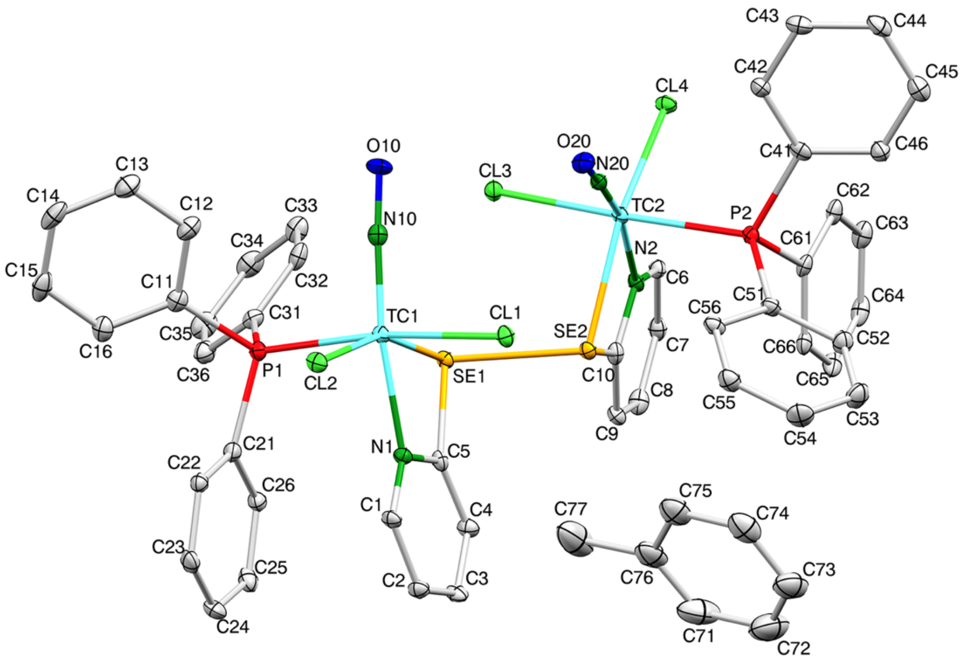

Figure S3. Ellipsoid representation of [{Tc(NO)Cl2(PPh3)}2{μ2-(2-pySe)2}] (**4**) x 1.5 toluene. The thermal ellipsoids are set at a 30% probability level. Hydrogen atoms are omitted for clarity.

Table S6. Bond lengths (Å) in [{Tc(NO)Cl2(PPh3)}2{μ2-(2-pySe)2}] (**4**) x 1.5 toluene.

Tc1	Se1	2.4730(6)	C41	C46	1.395(7)
Tc1	Cl1	2.4434(12)	C21	C26	1.399(7)
Tc1	Cl2	2.4101(12)	C21	C22	1.400(7)
Tc1	P1	2.4039(13)	C61	C62	1.398(7)
Tc1	N10	1.742(4)	C51	C56	1.392(7)
Tc1	N1	2.170(4)	C51	C52	1.400(7)
Tc2	Se2	2.4663(6)	C2	C3	1.390(7)
Tc2	Cl4	2.4089(12)	C23	C22	1.388(7)
Tc2	Cl3	2.4457(12)	C23	C24	1.372(8)
Tc2	P2	2.3953(13)	C56	C55	1.401(7)
Tc2	N2	2.195(4)	C42	C43	1.398(7)
Tc2	N20	1.743(4)	C26	C25	1.394(8)
Se1	Se2	2.5491(7)	C36	C35	1.386(8)
Se1	C5	1.931(5)	C24	C25	1.388(8)
Se2	C10	1.933(5)	C11	C12	1.397(8)
P2	C41	1.820(5)	C11	C16	1.395(7)
P2	C61	1.806(5)	C62	C63	1.393(7)
P2	C51	1.826(5)	C43	C44	1.395(8)
P1	C31	1.830(5)	C63	C64	1.380(8)
P1	C21	1.817(5)	C52	C53	1.385(8)
P1	C11	1.829(5)	C64	C65	1.378(8)
O10	N10	1.197(5)	C32	C33	1.394(7)
O20	N20	1.189(5)	C46	C45	1.387(8)
N2	C10	1.350(6)	C55	C54	1.376(8)
N2	C6	1.336(6)	C33	C34	1.384(8)
N1	C5	1.343(6)	C54	C53	1.391(8)

N1	C1	1.343(6)	C34	C35	1.392(9)
C4	C5	1.373(7)	C45	C44	1.378(8)
C4	C3	1.384(7)	C14	C15	1.372(9)
C31	C36	1.387(7)	C14	C13	1.379(9)
C31	C32	1.396(8)	C12	C13	1.391(8)
C10	C9	1.375(7)	C16	C15	1.396(8)
C9	C8	1.401(7)	C75	C74	1.389(10)
C1	C2	1.385(7)	C75	C76	1.404(10)
C6	C7	1.402(7)	C74	C73	1.368(11)
C8	C7	1.380(8)	C76	C77	1.509(11)
C66	C61	1.402(7)	C76	C71	1.372(12)
C66	C65	1.396(7)	C73	C72	1.365(12)
C41	C42	1.392(7)	C71	C72	1.411(13)

Table S7. Bond angles (°) in [{Tc(NO)Cl_2(PPh_3)}$_2${μ$_2$-(2-pySe)$_2$}] (**4**) x 1.5 toluene.

Cl1	Tc1	Se1	89.85(3)	N2	C10	Se2	107.8(4)
Cl2	Tc1	Se1	158.05(4)	N2	C10	C9	123.6(5)
Cl2	Tc1	Cl1	91.02(4)	C9	C10	Se2	128.5(4)
P1	Tc1	Se1	90.37(4)	C10	C9	C8	117.2(5)
P1	Tc1	Cl1	172.11(5)	N1	C1	C2	120.0(5)
P1	Tc1	Cl2	85.85(4)	N2	C6	C7	120.3(5)
N10	Tc1	Se1	98.00(14)	C7	C8	C9	119.5(5)
N10	Tc1	Cl1	94.20(14)	C65	C66	C61	120.1(5)
N10	Tc1	Cl2	103.81(14)	C42	C41	P2	121.4(4)
N10	Tc1	P1	93.58(14)	C42	C41	C46	118.8(5)
N10	Tc1	N1	167.53(17)	C46	C41	P2	119.7(4)
N1	Tc1	Se1	69.89(11)	C26	C21	P1	120.8(4)
N1	Tc1	Cl1	83.10(11)	C26	C21	C22	118.9(5)
N1	Tc1	Cl2	88.44(11)	C22	C21	P1	119.3(4)
N1	Tc1	P1	89.57(11)	C66	C61	P2	120.0(4)
Cl4	Tc2	Se2	158.19(4)	C62	C61	P2	120.4(4)
Cl4	Tc2	Cl3	88.25(4)	C62	C61	C66	118.6(5)
Cl3	Tc2	Se2	91.33(3)	C8	C7	C6	119.9(5)
P2	Tc2	Se2	92.16(3)	C56	C51	P2	119.4(4)
P2	Tc2	Cl4	85.98(4)	C56	C51	C52	119.3(5)
P2	Tc2	Cl3	172.34(5)	C52	C51	P2	121.3(4)
N2	Tc2	Se2	69.79(10)	C1	C2	C3	119.5(5)
N2	Tc2	Cl4	88.52(11)	C24	C23	C22	120.0(5)
N2	Tc2	Cl3	83.34(11)	C51	C56	C55	119.9(5)
N2	Tc2	P2	91.48(11)	C41	C42	C43	120.7(5)
N20	Tc2	Se2	96.99(13)	C25	C26	C21	119.8(5)
N20	Tc2	Cl4	104.76(13)	C4	C3	C2	120.3(5)
N20	Tc2	Cl3	95.66(14)	C35	C36	C31	120.2(5)
N20	Tc2	P2	90.68(14)	C23	C22	C21	120.7(5)
N20	Tc2	N2	166.67(17)	C23	C24	C25	120.4(5)
Tc1	Se1	Se2	105.10(2)	C12	C11	P1	119.2(4)
C5	Se1	Tc1	78.26(15)	C16	C11	P1	121.7(4)
C5	Se1	Se2	91.43(14)	C16	C11	C12	118.8(5)
Tc2	Se2	Se1	104.66(2)	C63	C62	C61	120.6(5)
C10	Se2	Tc2	79.03(15)	C44	C43	C42	119.6(5)

C10	Se2	Se1	93.10(14)	C24	C25	C26	120.3(5)
C41	P2	Tc2	115.52(17)	C64	C63	C62	120.1(5)
C41	P2	C51	102.7(2)	C53	C52	C51	120.4(5)
C61	P2	Tc2	108.98(16)	C65	C64	C63	120.1(5)
C61	P2	C41	105.9(2)	C33	C32	C31	120.2(5)
C61	P2	C51	104.7(2)	C45	C46	C41	120.6(5)
C51	P2	Tc2	117.93(17)	C54	C55	C56	120.2(5)
C31	P1	Tc1	114.52(17)	C34	C33	C32	119.9(6)
C21	P1	Tc1	109.55(17)	C55	C54	C53	120.2(5)
C21	P1	C31	107.1(2)	C64	C65	C66	120.5(5)
C21	P1	C11	105.8(2)	C33	C34	C35	119.8(5)
C11	P1	Tc1	117.40(18)	C44	C45	C46	120.5(5)
C11	P1	C31	101.6(2)	C15	C14	C13	119.7(5)
C10	N2	Tc2	103.3(3)	C13	C12	C11	120.4(6)
C6	N2	Tc2	137.2(3)	C11	C16	C15	119.7(6)
C6	N2	C10	119.4(4)	C52	C53	C54	119.9(5)
O10	N10	Tc1	176.5(4)	C45	C44	C43	119.9(5)
O20	N20	Tc2	176.7(4)	C14	C15	C16	121.0(6)
C5	N1	Tc1	103.8(3)	C14	C13	C12	120.3(6)
C5	N1	C1	119.7(4)	C36	C35	C34	120.3(6)
C1	N1	Tc1	136.4(3)	C74	C75	C76	120.4(8)
C5	C4	C3	116.8(5)	C73	C74	C75	120.9(7)
C36	C31	P1	123.1(4)	C75	C76	C77	120.4(8)
C36	C31	C32	119.4(5)	C71	C76	C75	118.0(7)
C32	C31	P1	117.5(4)	C71	C76	C77	121.7(8)
N1	C5	Se1	108.0(3)	C72	C73	C74	119.6(8)
N1	C5	C4	123.7(5)	C76	C71	C72	120.9(8)
C4	C5	Se1	128.4(4)	C73	C72	C71	120.2(9)

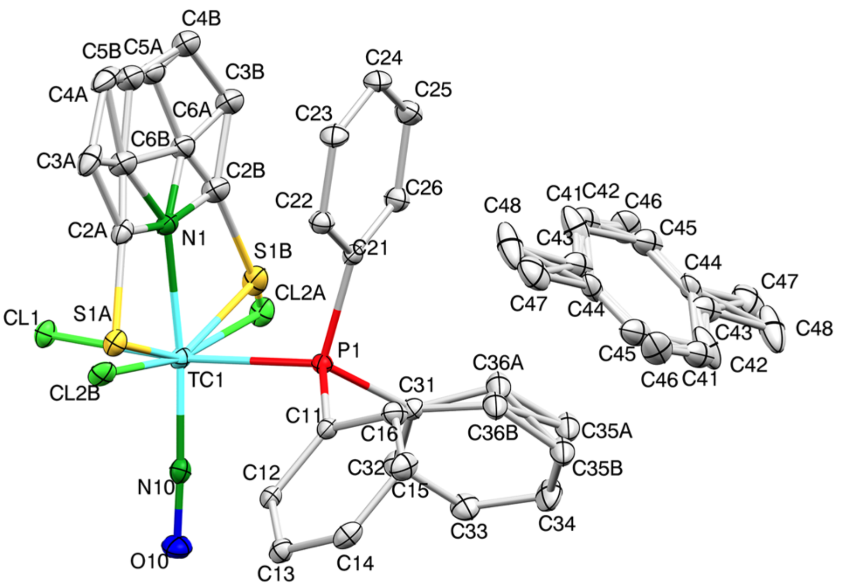

Figure S4. Ellipsoid representation of the complexes contained in [Tc^{II}(NO)Cl_2(PPh_3)(2-pyS)] (**6**) x 0.5 toluene, also illustrating disorders in the PyS^- ligand, two of the phenyl rings and the solvent toluene. The thermal ellipsoids are set at a 30% probability level. Hydrogen atoms are omitted for clarity.

Table S8. Bond lengths (Å) in [Tc^{II}(NO)Cl_2(PPh_3)(2-pyS)] (**6**) x 0.5 toluene.

Cl1	Tc1	2.4129(7)	C24	C25	1.379(5)
P1	C21	1.813(3)	C34	C35A	1.41(2)
P1	C11	1.823(3)	C34	C35B	1.38(2)
P1	C31	1.829(3)	Tc1	Cl2A	2.3901(11)
P1	Tc1	2.4642(7)	Tc1	S1A	2.3445(15)
O10	N10	1.176(3)	Tc1	Cl2B	2.587(8)
N1	Tc1	2.140(2)	Tc1	S1B	2.203(6)
N1	C2A	1.347(4)	S1A	C2A	1.757(4)
N1	C6A	1.326(4)	C2A	C3A	1.391(5)
N1	C2B	1.41(3)	C5A	C6A	1.383(6)
N1	C6B	1.41(2)	C5A	C4A	1.373(6)
N10	Tc1	1.754(2)	C36A	C35A	1.391(14)
C21	C22	1.397(4)	C4A	C3A	1.390(6)
C21	C26	1.391(4)	C47	C44	1.54(4)
C32	C31	1.388(4)	C46	C44	1.384(11)
C32	C33	1.390(4)	C46	C42[1]	1.439(15)
C11	C12	1.386(4)	C44	C42	1.384(15)
C11	C16	1.397(4)	S1B	C2B	1.78(3)
C12	C13	1.389(4)	C5B	C4B	1.39(4)
C22	C23	1.388(4)	C5B	C6B	1.34(4)
C31	C36A	1.40(3)	C35B	C36B	1.393(14)
C31	C36B	1.38(3)	C4B	C3B	1.43(3)
C13	C14	1.382(4)	C2B	C3B	1.38(3)
C26	C25	1.389(4)	C43	C41	1.388(19)
C14	C15	1.381(4)	C43	C48	1.59(4)
C16	C15	1.379(4)	C43	C45	1.371(14)
C33	C34	1.371(5)	C41	C45[1]	1.39(2)
C23	C24	1.385(4)			

[1] 2-x,1-y,1-z

Table S9. Bond angles (°) in $[Tc^{II}(NO)Cl_2(PPh_3)(2\text{-}pyS)]$ (**6**) x 0.5 toluene.

C21	P1	C11	105.48(12)	N1	Tc1	S1B	72.0(2)
C21	P1	C31	107.78(12)	N10	Tc1	Cl1	95.15(7)
C21	P1	Tc1	109.19(9)	N10	Tc1	P1	91.57(7)
C11	P1	C31	101.32(12)	N10	Tc1	N1	170.99(10)
C11	P1	Tc1	116.35(9)	N10	Tc1	Cl2A	101.21(8)
C31	P1	Tc1	115.87(9)	N10	Tc1	S1A	102.29(8)
C2A	N1	Tc1	99.73(19)	N10	Tc1	Cl2B	91.02(19)
C6A	N1	Tc1	138.1(2)	N10	Tc1	S1B	117.0(2)
C6A	N1	C2A	121.8(3)	Cl2A	Tc1	Cl1	90.89(3)
C2B	N1	Tc1	97.2(10)	Cl2A	Tc1	P1	85.28(3)
C6B	N1	Tc1	145.8(10)	S1A	Tc1	Cl1	93.09(4)
C6B	N1	C2B	116.1(14)	S1A	Tc1	P1	87.97(4)
O10	N10	Tc1	177.0(2)	S1A	Tc1	Cl2A	155.70(4)
C22	C21	P1	119.0(2)	S1B	Tc1	Cl1	92.46(18)
C26	C21	P1	121.2(2)	S1B	Tc1	P1	82.18(18)
C26	C21	C22	119.3(3)	S1B	Tc1	Cl2B	150.9(3)
C31	C32	C33	120.3(3)	C2A	S1A	Tc1	81.52(12)
C12	C11	P1	121.6(2)	N1	C2A	S1A	109.3(2)
C12	C11	C16	119.1(3)	N1	C2A	C3A	121.0(3)
C16	C11	P1	119.0(2)	C3A	C2A	S1A	129.7(3)
C11	C12	C13	120.3(2)	C4A	C5A	C6A	119.3(4)
C23	C22	C21	120.2(3)	N1	C6A	C5A	120.0(4)
C32	C31	P1	120.7(2)	C35A	C36A	C31	120.5(16)
C32	C31	C36A	118.8(13)	C5A	C4A	C3A	120.8(4)
C36A	C31	P1	120.4(12)	C36A	C35A	C34	118.7(10)
C36B	C31	P1	118.3(13)	C4A	C3A	C2A	117.1(4)
C36B	C31	C32	119.1(13)	C44	C46	C42[1]	117.4(12)
C14	C13	C12	120.1(3)	C46	C44	C47	125.5(13)
C25	C26	C21	120.0(3)	C42	C44	C47	113.0(16)
C15	C14	C13	119.8(3)	C42	C44	C46	121.4(12)
C15	C16	C11	120.2(3)	C44	C42	C46[1]	121.2(16)
C16	C15	C14	120.4(3)	C2B	S1B	Tc1	85.0(8)
C34	C33	C32	120.3(3)	C6B	C5B	C4B	123(4)
C24	C23	C22	120.0(3)	C34	C35B	C36B	120.7(10)
C25	C24	C23	120.1(3)	C5B	C4B	C3B	118(3)
C24	C25	C26	120.4(3)	C31	C36B	C35B	119.5(16)
C33	C34	C35A	119.6(9)	N1	C2B	S1B	105.7(13)
C33	C34	C35B	118.8(9)	C3B	C2B	N1	124(2)
Cl1	Tc1	P1	172.81(2)	C3B	C2B	S1B	130(2)
Cl1	Tc1	Cl2B	92.5(2)	C5B	C6B	N1	121(3)
P1	Tc1	Cl2B	89.9(2)	C2B	C3B	C4B	117(2)
N1	Tc1	Cl1	83.58(6)	C41	C43	C48	126.4(18)
N1	Tc1	P1	90.18(6)	C45	C43	C41	112.0(17)
N1	Tc1	Cl2A	87.75(7)	C45	C43	C48	121.5(19)
N1	Tc1	S1A	68.94(7)	C43	C41	C45[1]	121.6(15)
N1	Tc1	Cl2B	80.14(18)				

[1] 2-x,1-y,1-z

2. Selected Spectroscopic Data

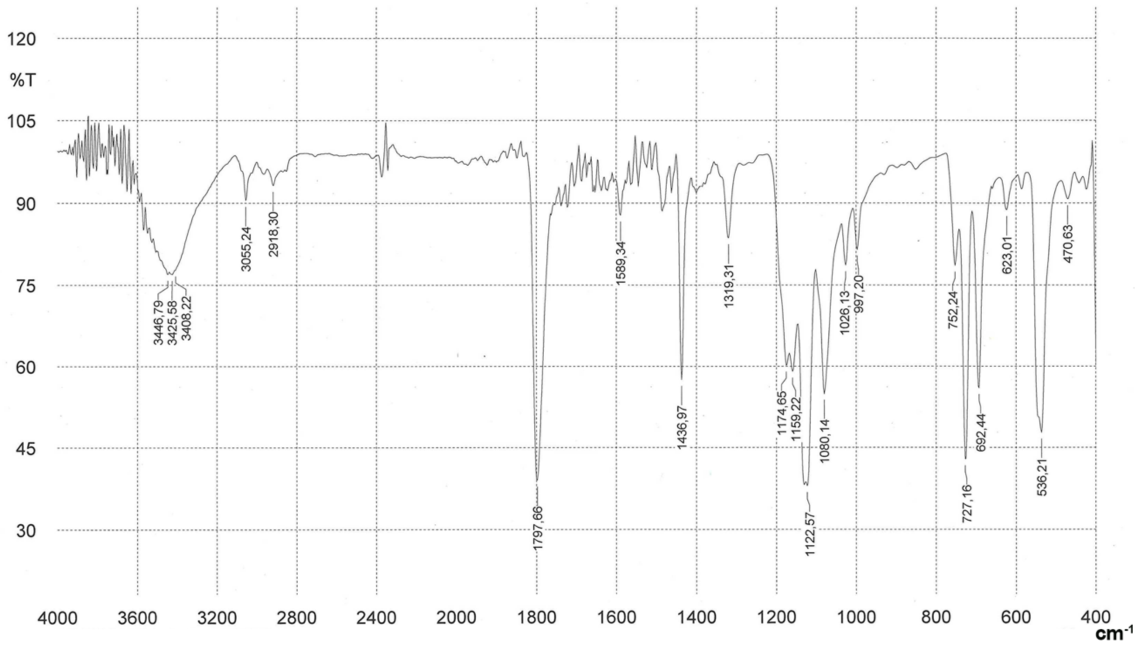

Figure S5. IR spectrum (KBr) of [Tc(NO)Cl_3($OPPh_3$)$_2$] (**2**).

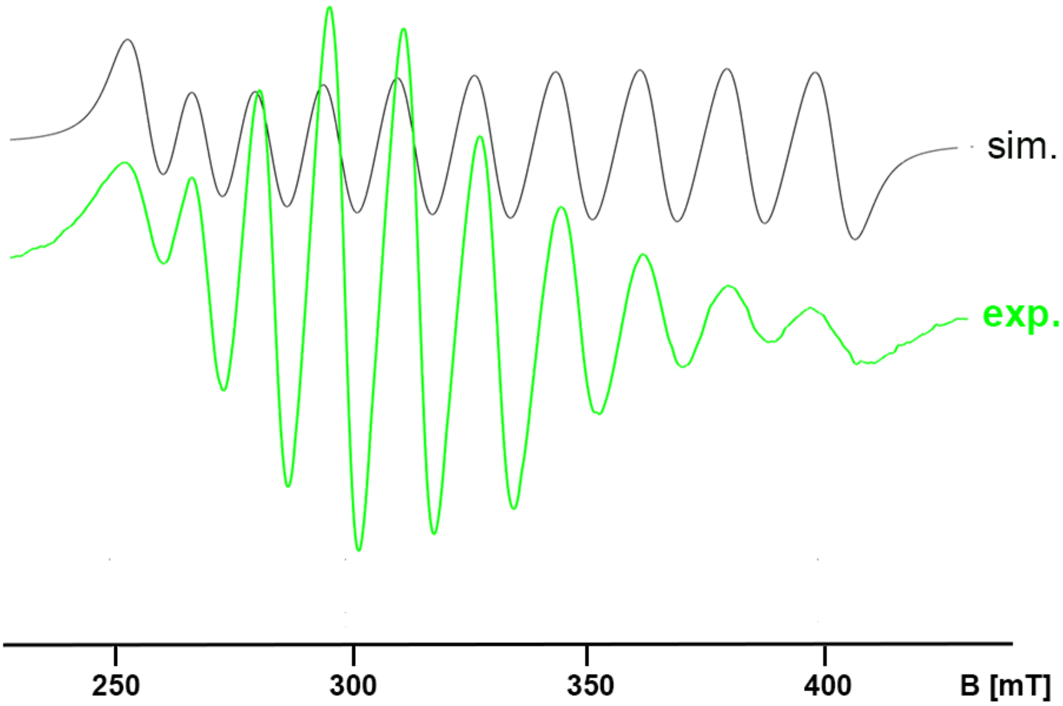

Figure S6. Room-temperature EPR spectrum of [Tc(NO)Cl_3($OPPh_3$)$_2$] (**2**) in $CHCl_3$.

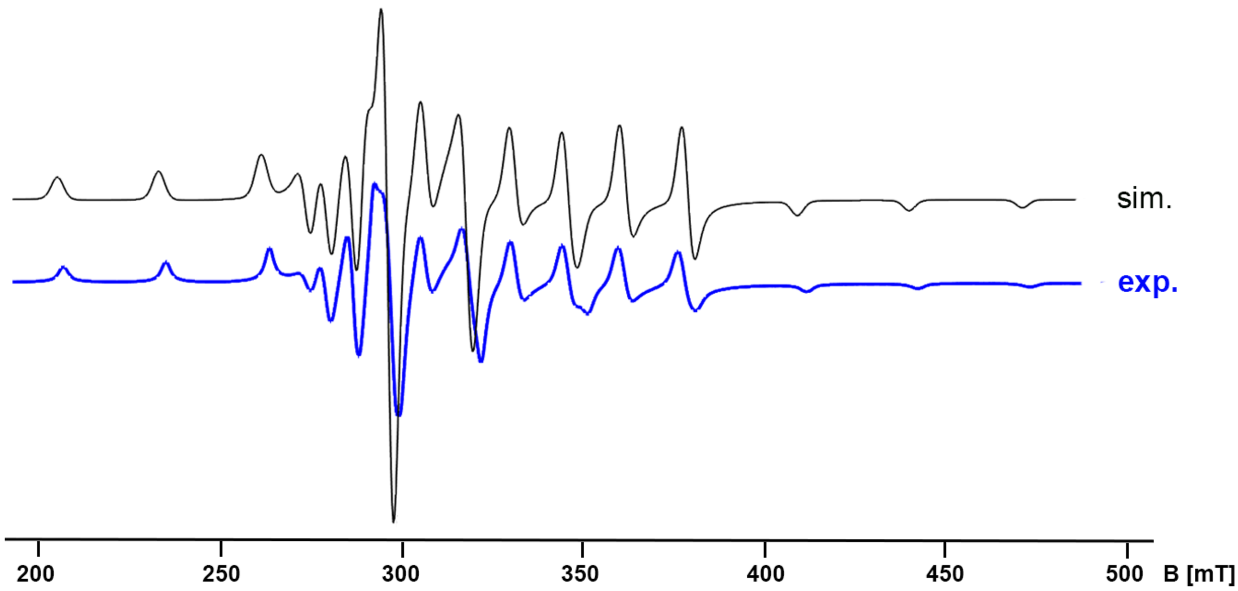

Figure S7. Frozen-solution EPR spectrum (T = 78 K) of $[Tc(NO)Cl_3(OPPh_3)_2]$ (**2**) in $CHCl_3$.

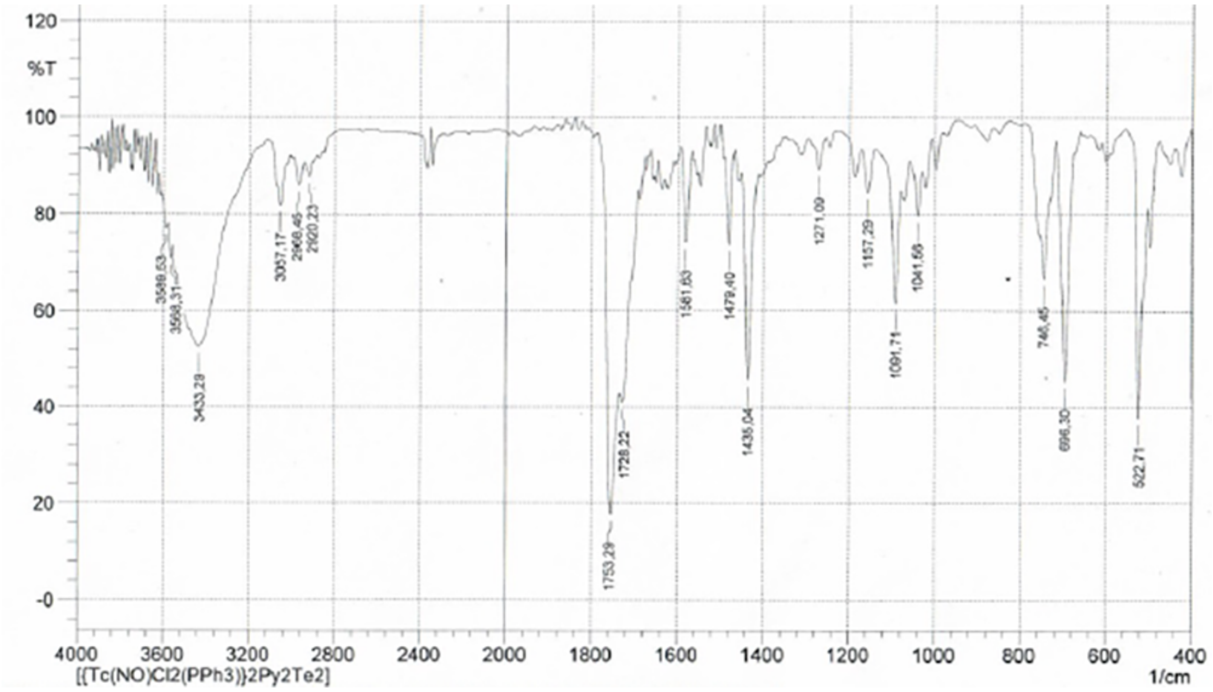

Figure S8. IR spectrum (KBr) of $[\{TcI(NO)Cl_2(PPh_3)\}_2\{\mu_2\text{-}(2\text{-pyTe})_2\}]$ (**3**).

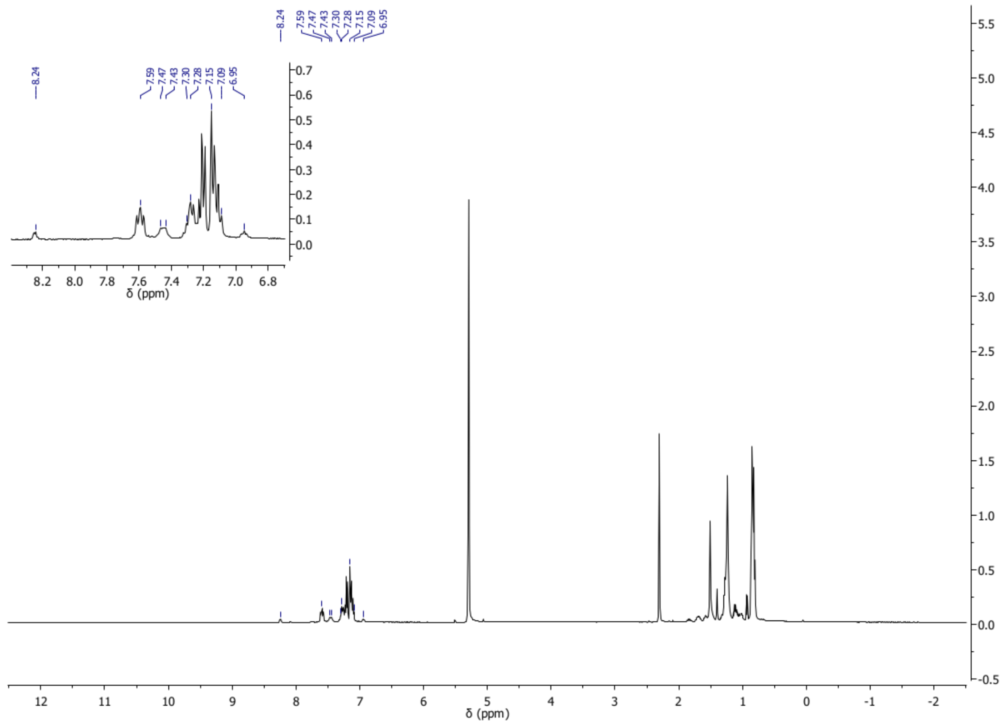

Figure S9. ^{1}H NMR spectra of [{Tc^{I}(NO)Cl$_2$(PPh$_3$)}$_2${μ$_2$-(2-pyTe)$_2$}] (**3**) in CD_2Cl_2.

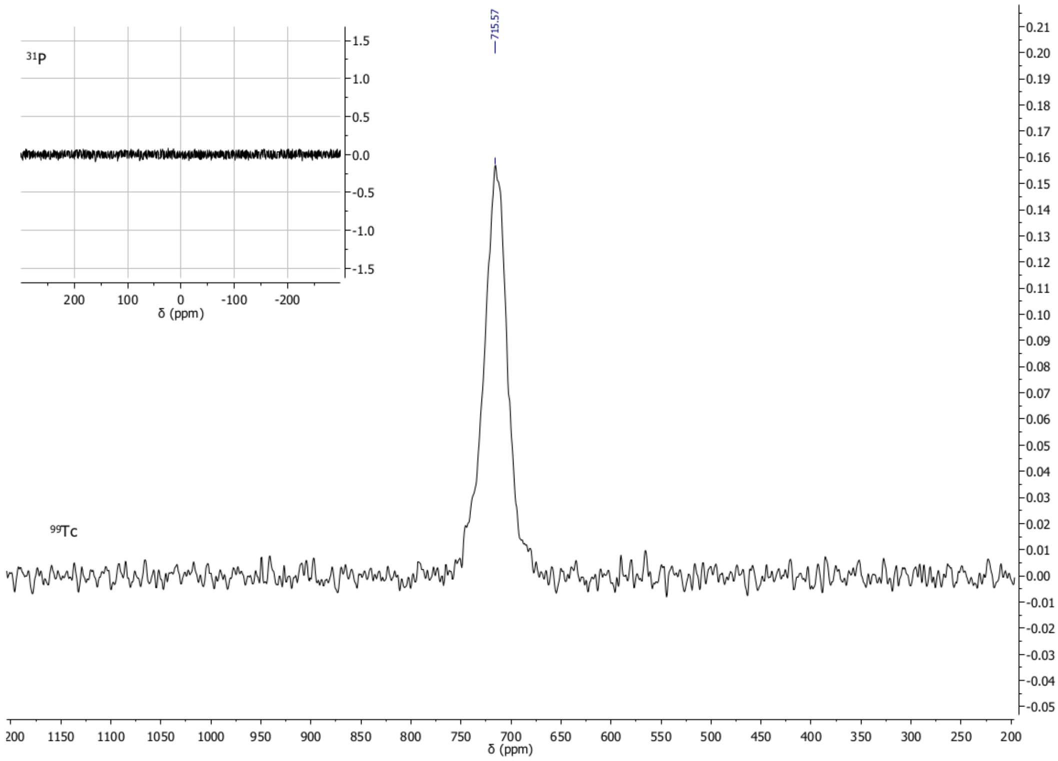

Figure S10. ^{99}Tc and ^{31}P NMR (not visible) spectra of [{Tc^{I}(NO)Cl$_2$(PPh$_3$)}$_2${μ$_2$-(2-pyTe)$_2$}] (**3**) in CD_2Cl_2.

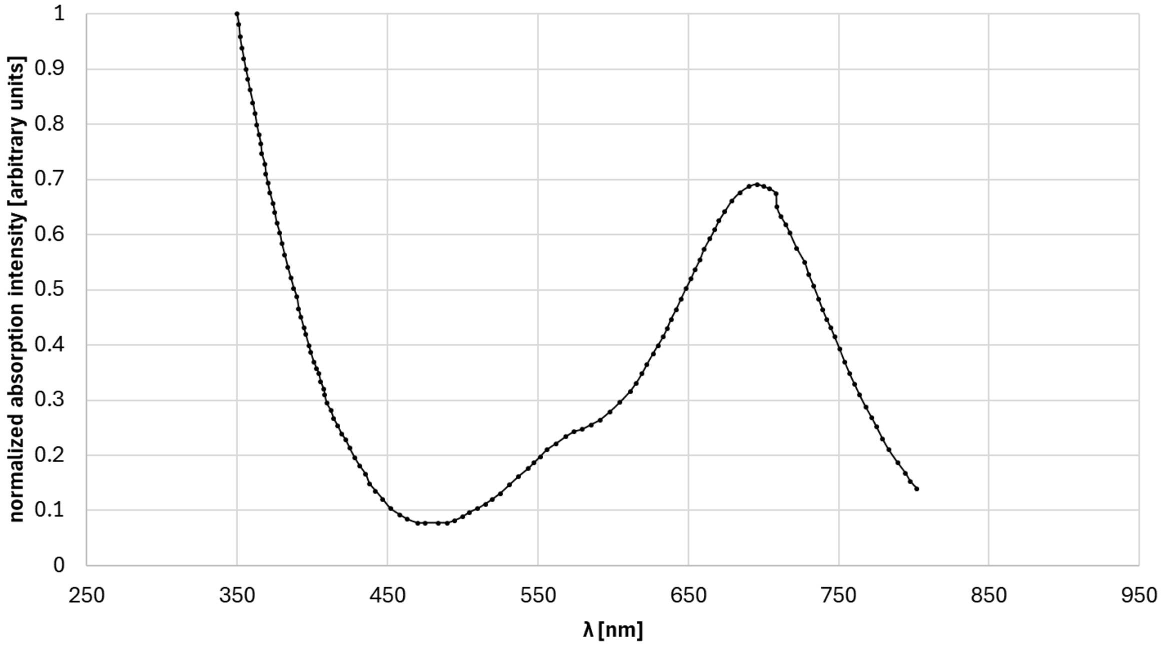

Figure S11. . Normalized experimental UV-Vis spectrum of [{Tc^I(NO)Cl$_2$(PPh$_3$)}$_2${μ$_2$-{2-pyTe)$_2$}].

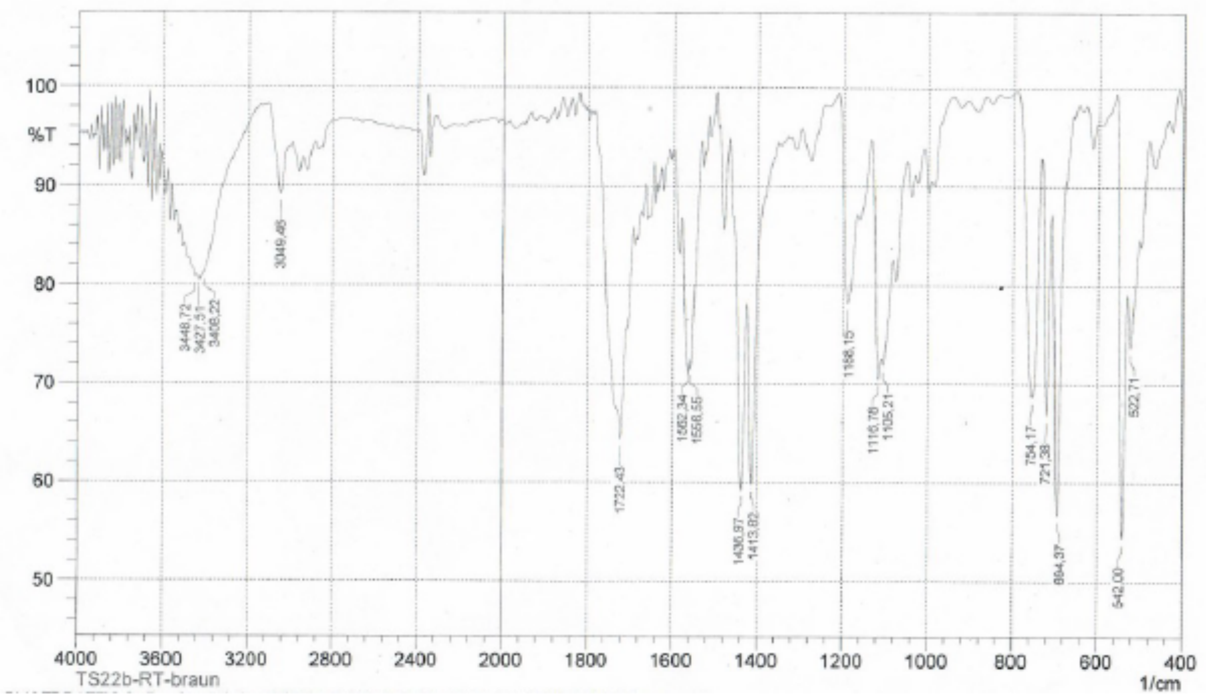

Figure S12. IR spectrum (KBr) of of [{Tc^I(NO)Cl$_2$(PPh$_3$)}$_2${μ$_2$-(2-pySe)$_2$}] (**4**).

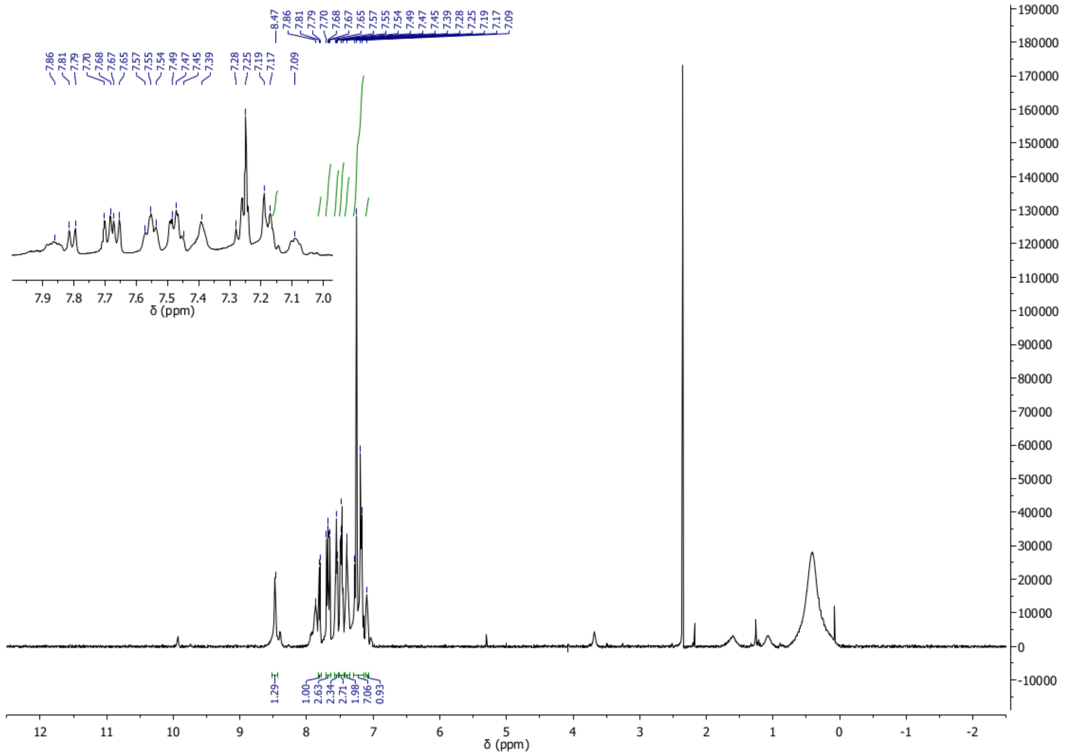

Figure S13. ^{1}H NMR spectra of of [{$Tc^{I}(NO)Cl_2(PPh_3)$}$_2${μ_2-(2-pySe)$_2$}] (**4**) in DMSO-D_6.

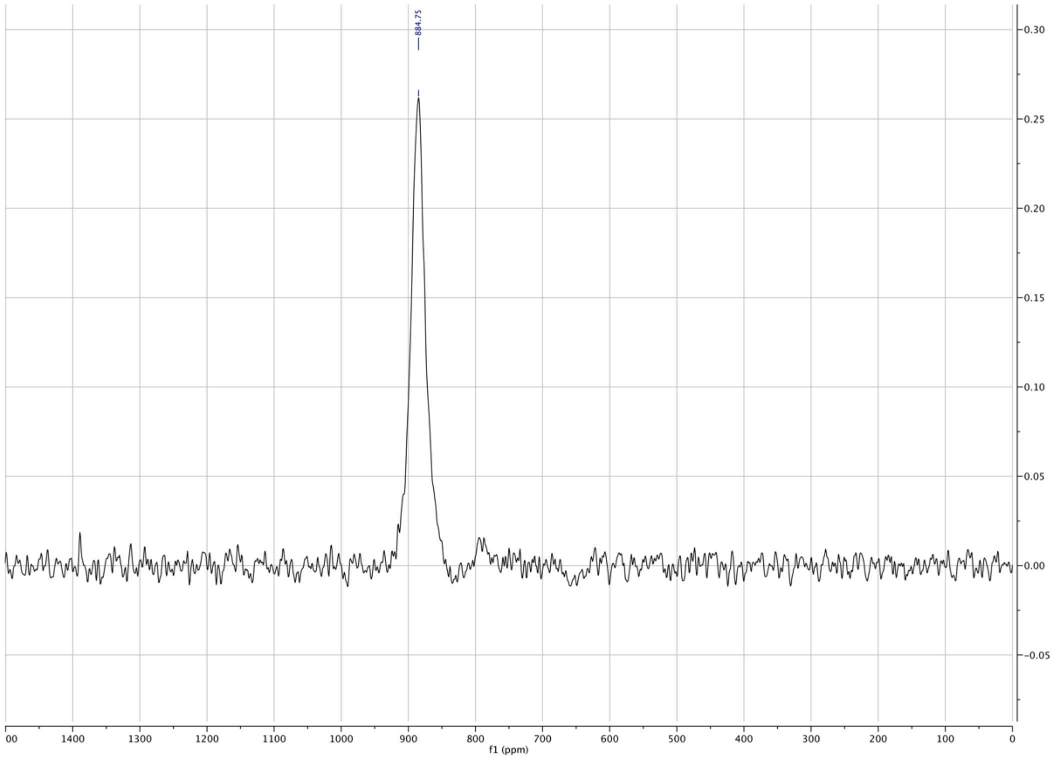

Figure S14. ^{99}Tc spectrum of [{$Tc^{I}(NO)Cl_2(PPh_3)$}$_2${μ_2-(2-pySe)$_2$}] (**4**) DMSO-D_6.

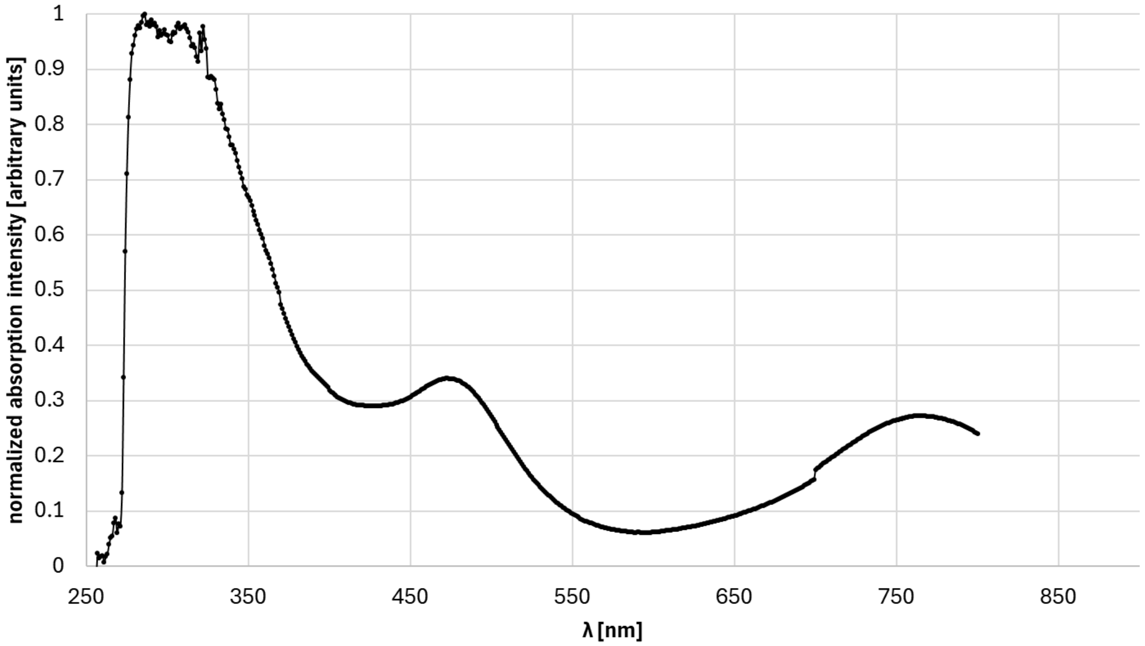

Figure S15. Normalized experimental UV-Vis spectrum of [Tc^{II}(NO)Cl_2(PPh_3)(2-pySe)].

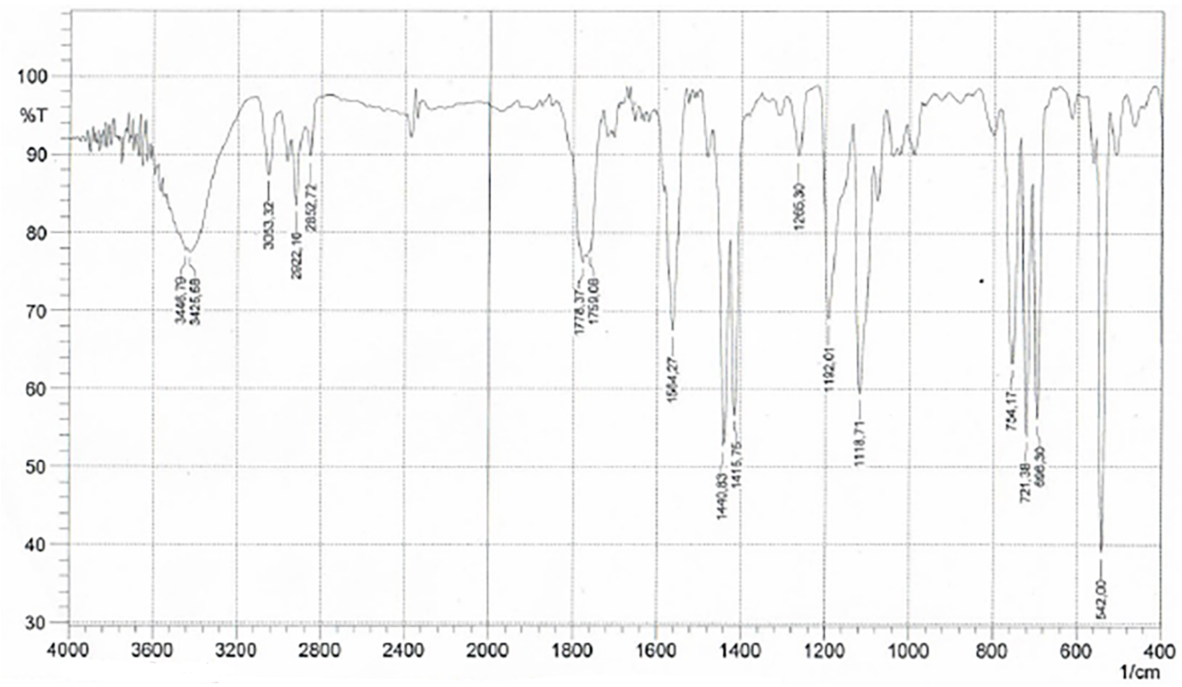

Figure S16. IR spectrum (KBr) of [Tc(NO)Cl_2(PPh_3)(PySe)].

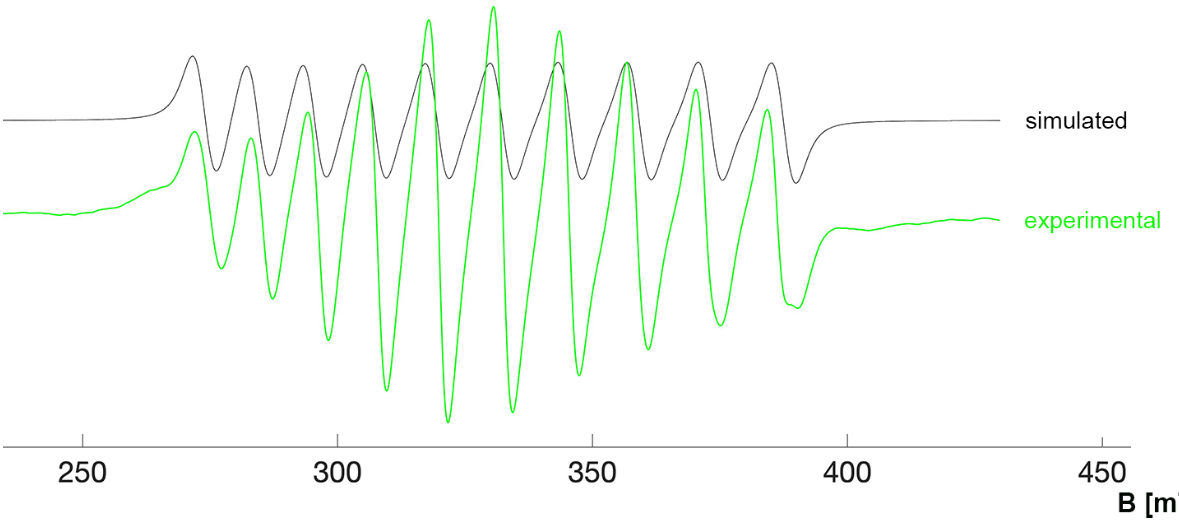

Figure S17. Room-temperature X-band EPR spectrum of $[Tc(NO)Cl_2(PPh_3)(PySe)]$ in $CHCl_3$.

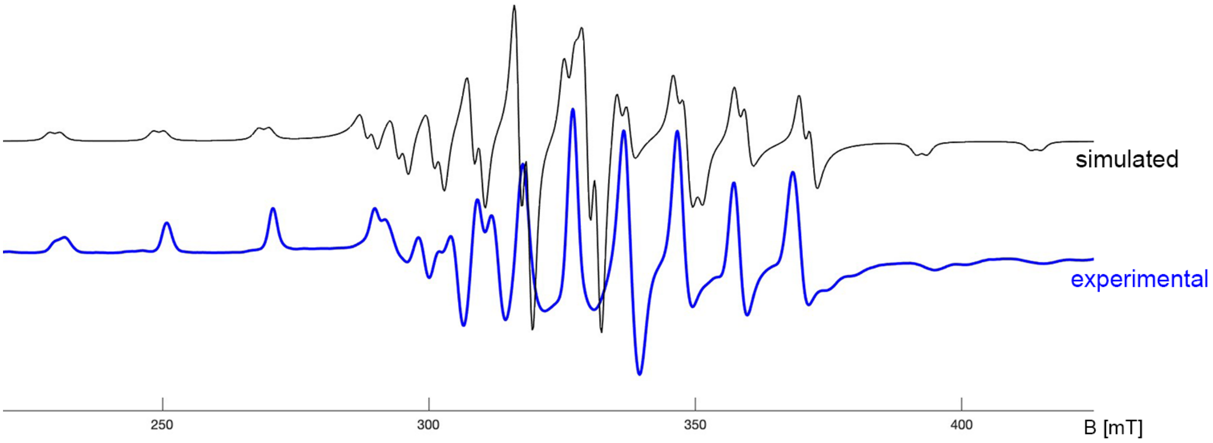

Figure S18. Frozen-solution (T = 77 K) X-band EPR spectrum of $[Tc(NO)Cl_2(PPh_3)(PySe)]$ in $CHCl_3$.

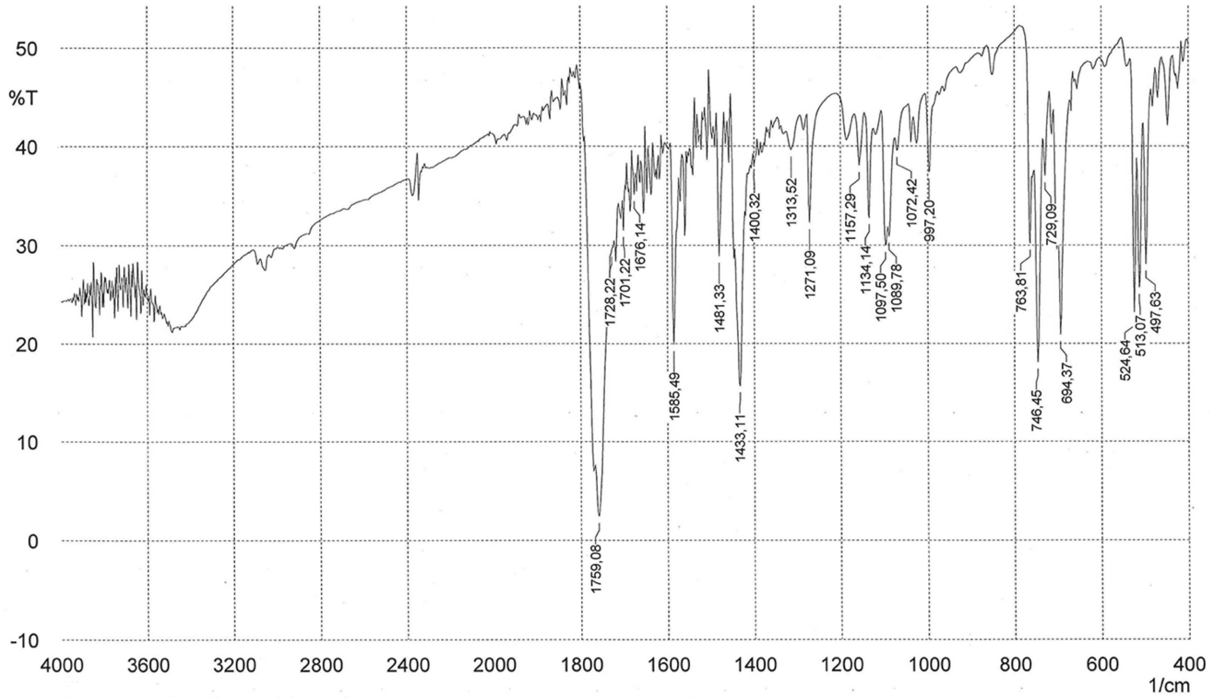

Figure S19. IR spectrum (KBr) of [Tc(NO)Cl_2(PPh_3)(PyS)].

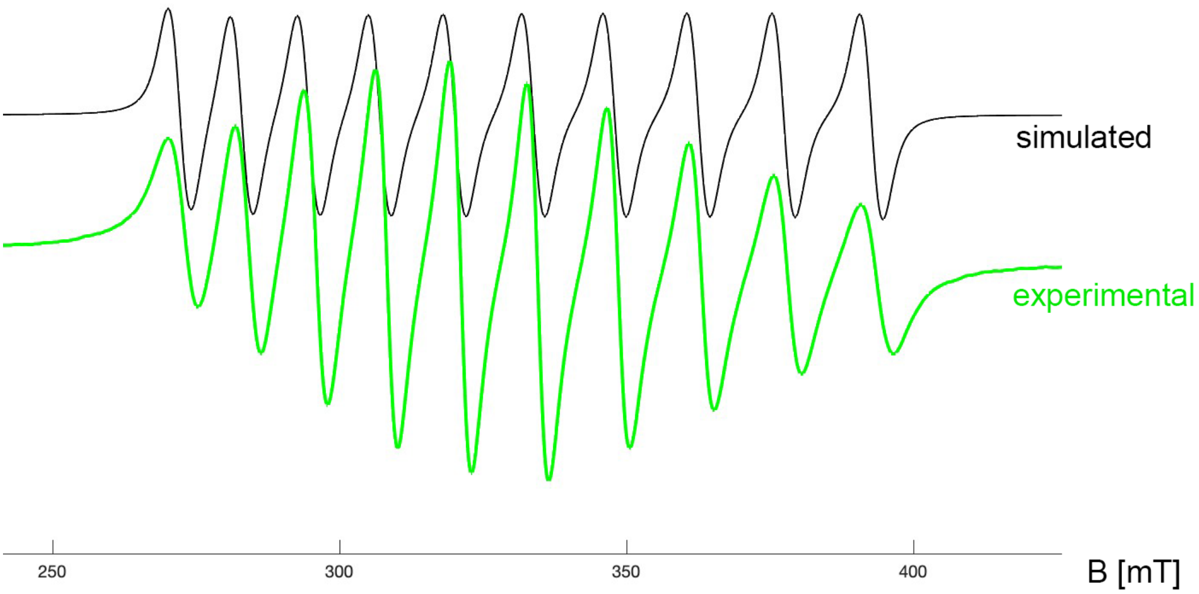

Figure S20. Room-temperature X-band EPR spectrum of [Tc(NO)Cl_2(PPh_3)(PyS)] in $CHCl_3$.

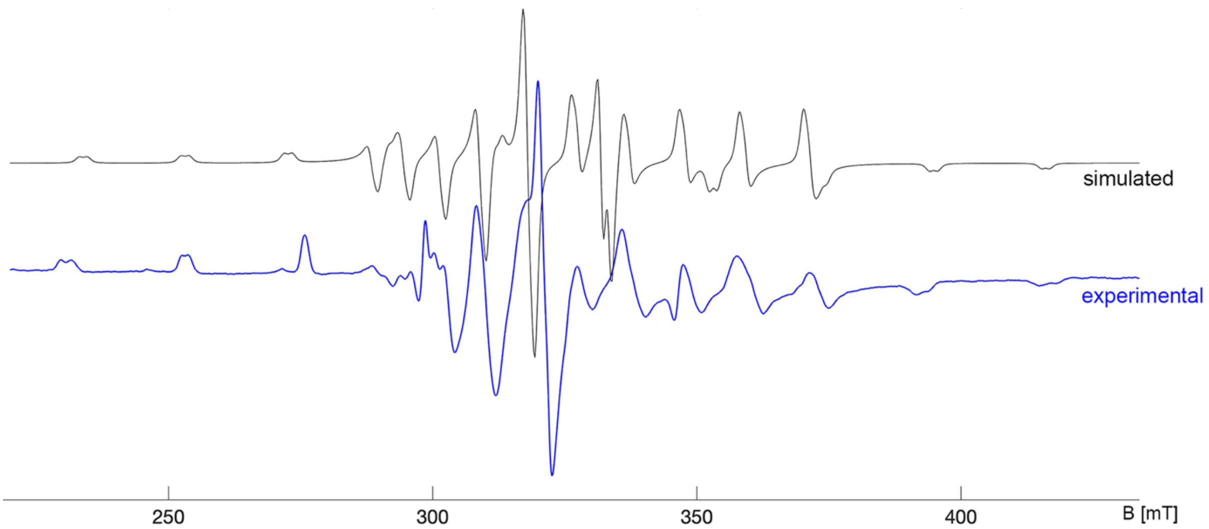

Figure S21. Frozen-solution (T = 77 K) X-band EPR spectrum of [Tc(NO)Cl_2(PPh_3)(PyS)] in $CHCl_3$.

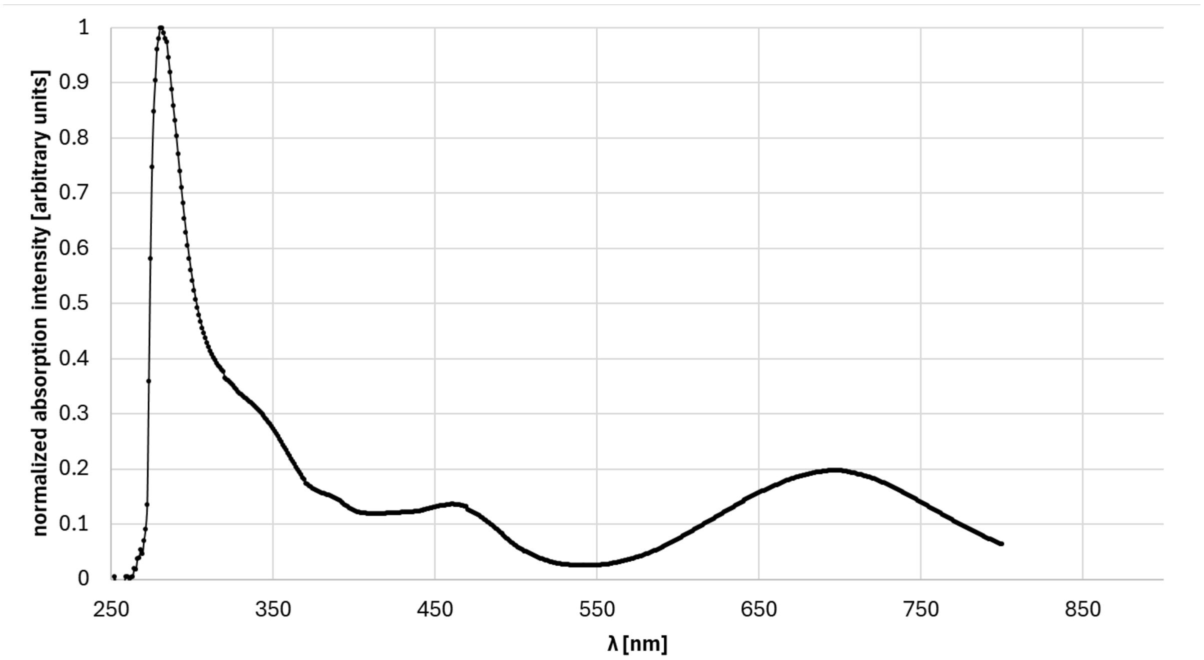

Figure S22. Normalized experimental UV-Vis spectrum of [Tc^{II}(NO)Cl_2(PPh_3)(2-pyS)].

3. Computational Chemistry

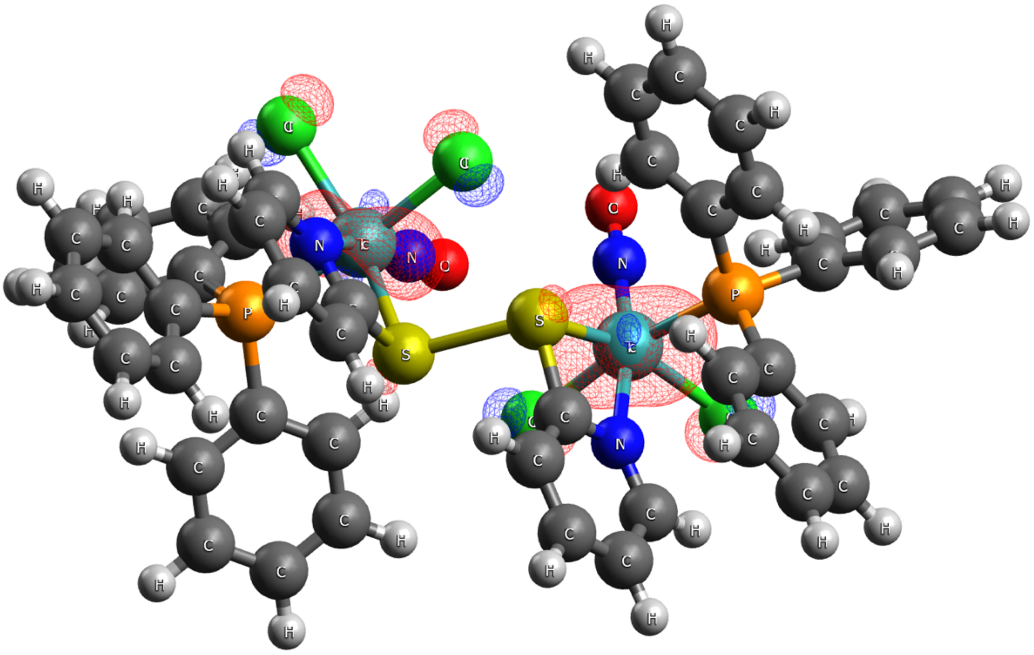

Figure S23. HOMO of $[\{Tc^{I}(NO)Cl_2(PPh_3)\}_2\{\mu_2\text{-}\{2\text{-}pyS\}_2\}]$ at an isosurface value of 0.05. B3LYP-GD3B/StuttgartRSC(Tc)/StuttgartRLC+STO-3G(S)/6-31G*(C,N,P,Cl)/6-31G(H) level.

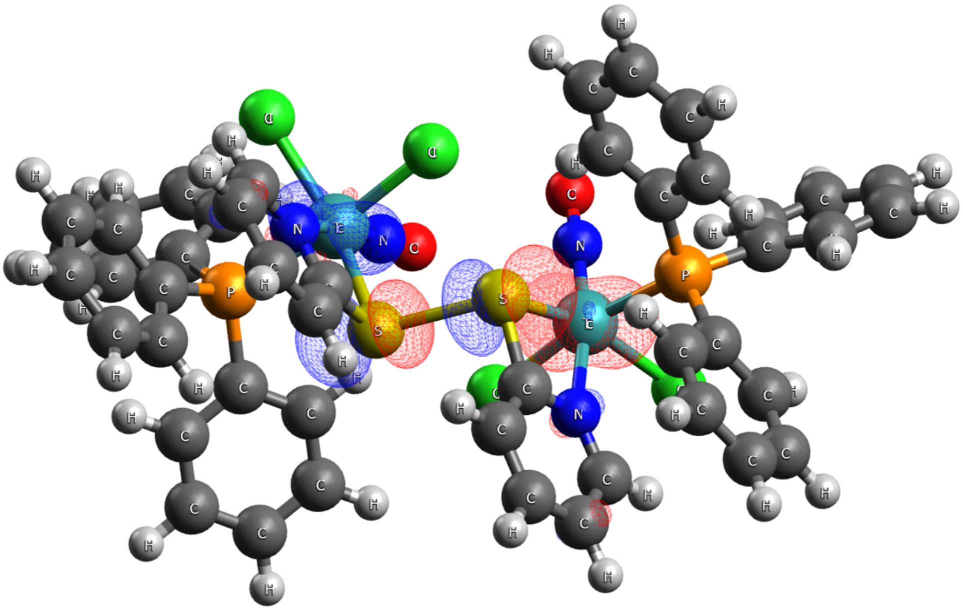

Figure S24. LUMO of $[\{Tc^{I}(NO)Cl_2(PPh_3)\}_2\{\mu_2\text{-}\{2\text{-}pyS\}_2\}]$ at an isosurface value of 0.05. B3LYP-GD3B/StuttgartRSC(Tc)/StuttgartRLC+STO-3G(S)/6-31G*(C,N,P,Cl)/6-31G(H) level.

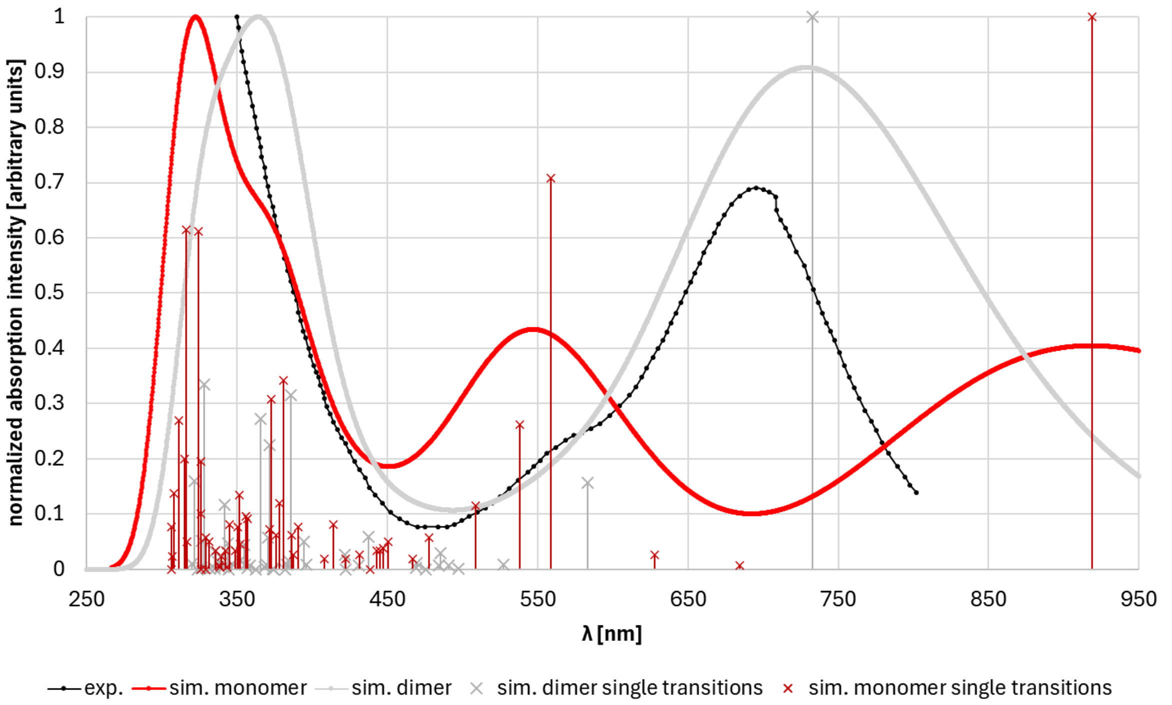

Figure S25. Comparison between experimental UV-Vis spectrum of [{Tc^{I}(NO)Cl_2(PPh_3)}$_2${μ$_2$-{2-pyTe)$_2$}] and simulated UV-Vis spectra of the (hypothetical) monomeric [Tc^{II}(NO)Cl_2(PPh_3)(2-pyTe)] or dimeric [{Tc^{I}(NO)Cl_2(PPh_3)}$_2${μ$_2$-{2-pyTe)$_2$}]; 50 transitions were respectively considered (indicated by lines). B3LYP-GD3B/StuttgartRSC(Tc)/StuttgartRLC+STO-3G(Te)/6-31G*(C,N,P,Cl)/6-31G(H) level. The spectral signature in the visible part of the spectrum is consistent with the presence of the dimeric Tc(I) instead of the (hypothetical) monomeric Tc(II) compound.

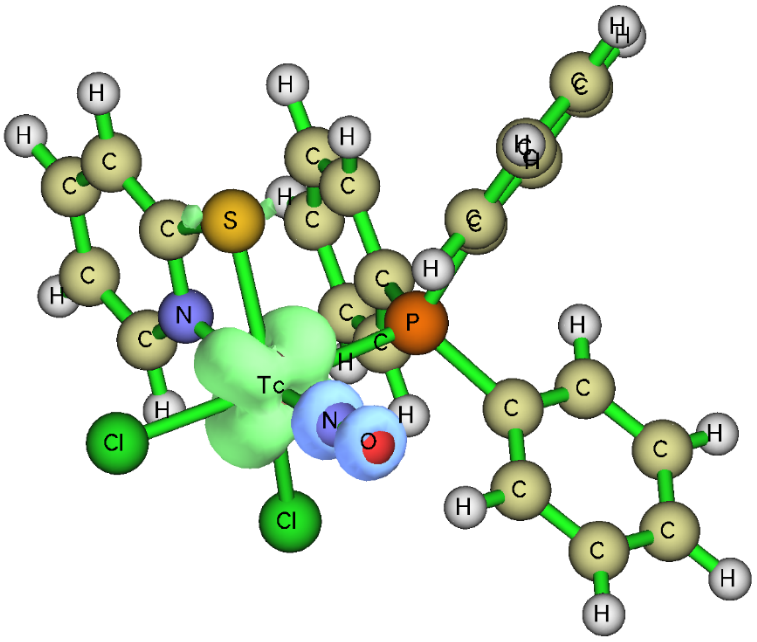

Figure S26. Spin density of [Tc(NO)Cl_2(PPh_3)(2-pyS)] at an isosurface level of 0.01. B3LYP-GD3B/StuttgartRSC(Tc)/StuttgartRLC+STO-3G(S)/6-31G*(C,N,P,Cl)/6-31G(H) level.

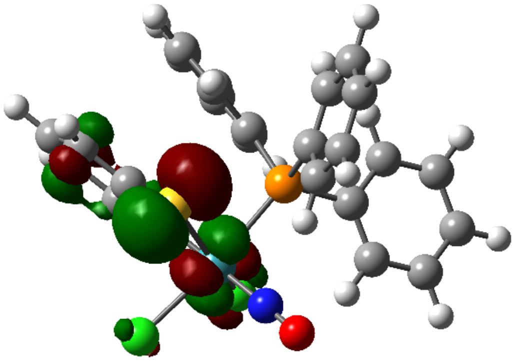

Figure S27. SOMO of [Tc(NO)Cl_2(PPh_3)(2-pyS)]. B3LYP-GD3B/StuttgartRSC(Tc)/StuttgartRLC+STO-3G(S)/6-31G*(C,N,P,Cl)/6-31G(H) level.

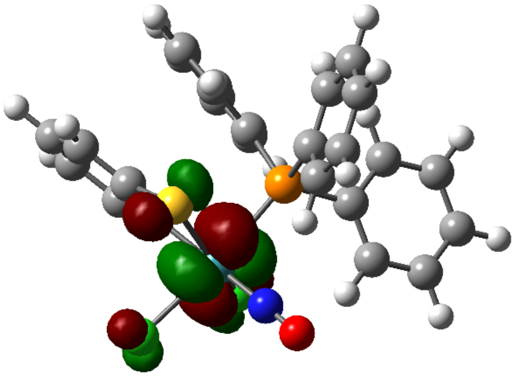

Figure S28. LUMO of [Tc(NO)Cl_2(PPh_3)(2-pyS)]. B3LYP-GD3B/StuttgartRSC(Tc)/StuttgartRLC+STO-3G(S)/6-31G*(C,N,P,Cl)/6-31G(H) level.

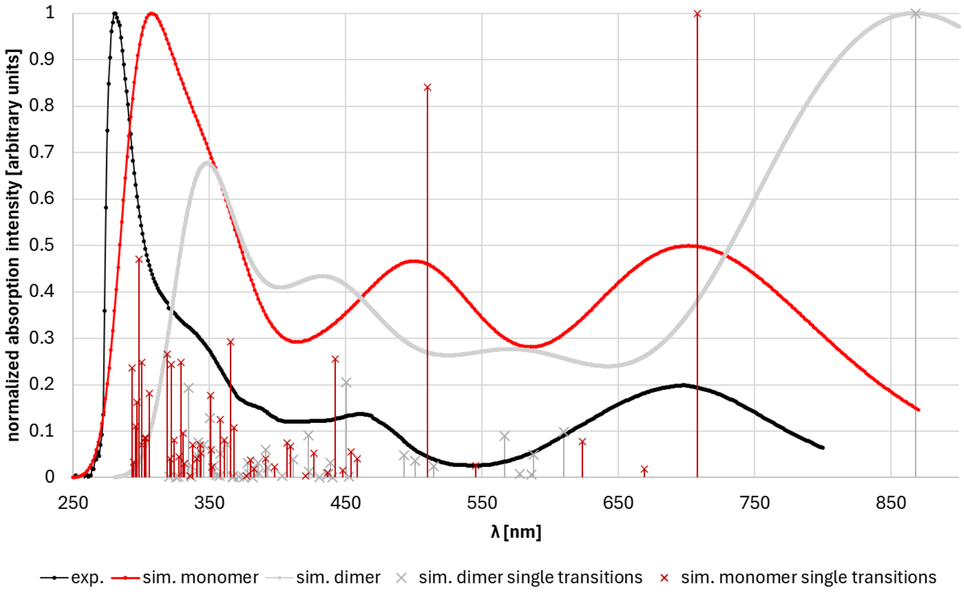

Figure S29 Comparison between experimental UV-Vis spectrum of [Tc^{II}(NO)Cl_2(PPh_3)(2-pyS)] and simulated UV-Vis spectra of the monomeric [Tc^{II}(NO)Cl_2(PPh_3)(2-pyS)] or (hypothetic) dimeric [{Tc^{I}(NO)Cl_2(PPh_3)}$_2${μ_2-{2-pyS)$_2$}]; 50 transitions were respectively considered (indicated by lines). B3LYP-GD3B/StuttgartRSC(Tc)/StuttgartRLC+STO-3G(S)/6-31G*(C,N,P,Cl)/6-31G(H) level. The spectral signature in the visible part of the spectrum is consistent with the presence of the monomeric Tc(II) instead of the (hypothetical) dimeric Tc(I) compound.

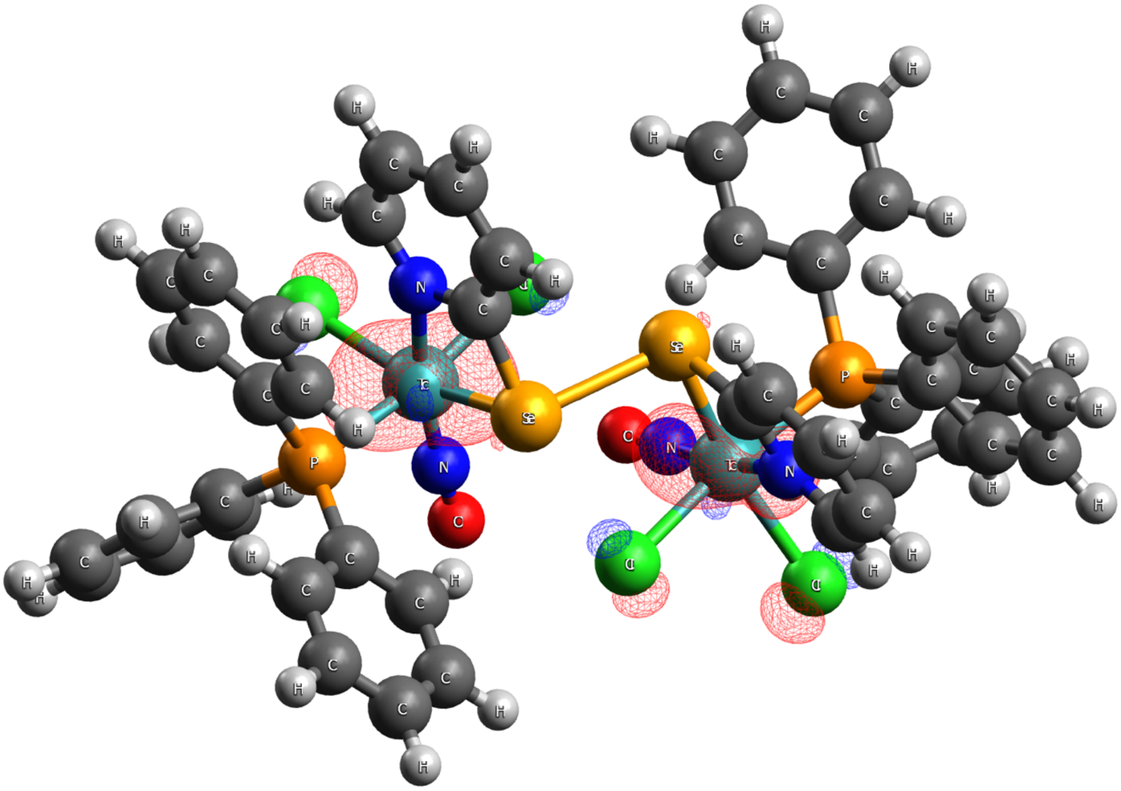

Figure S30. HOMO of [{Tc^{I}(NO)Cl_2(PPh_3)}$_2${μ_2-{2-pySe)$_2$}] at an isosurface value of 0.05. B3LYP-GD3B/StuttgartRSC(Tc)/StuttgartRLC+STO-3G(Se)/6-31G*(C,N,P,Cl)/6-31G(H) level.

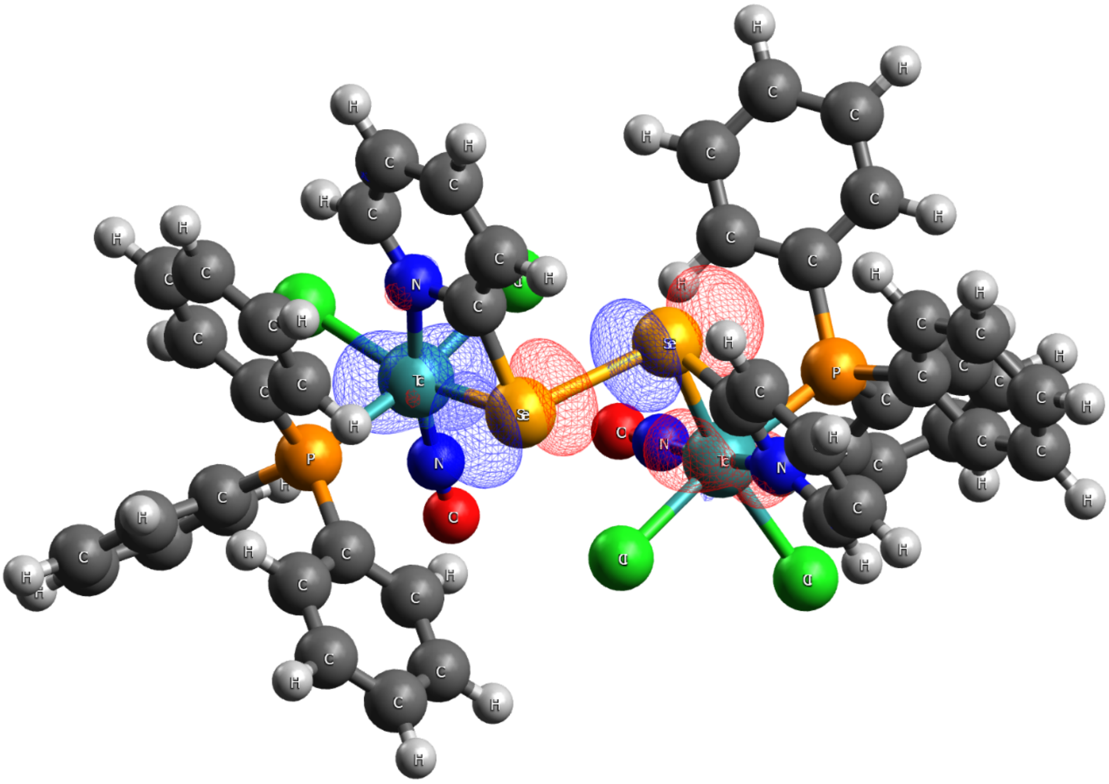

Figure S31. LUMO of [{Tc^{I}(NO)Cl_2(PPh_3)}$_2${μ_2-{2-pySe)$_2$}] at an isosurface value of 0.05. B3LYP-GD3B/StuttgartRSC(Tc)/StuttgartRLC+STO-3G(Se)/6-31G*(C,N,P,Cl)/6-31G(H) level.

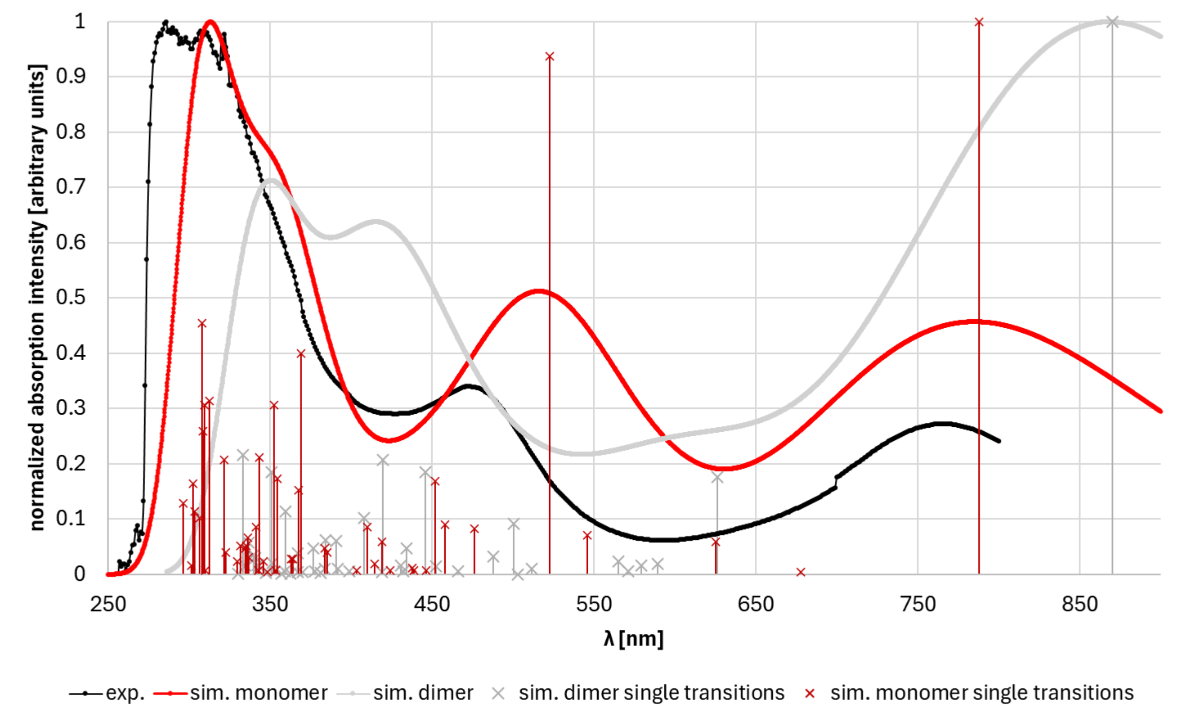

Figure S32. Comparison between experimental UV-Vis spectrum of [Tc^{II}(NO)Cl$_2$(PPh$_3$)(2-pySe)] and simulated UV-Vis spectra of the monomeric [Tc^{II}(NO)Cl$_2$(PPh$_3$)(2-pySe)] or dimeric [{Tc^{I}(NO)Cl$_2$(PPh$_3$)}$_2${μ$_2$-{2-pySe)$_2$}]; 50 transitions were respectively considered (indicated by lines). B3LYP-GD3B/StuttgartRSC(Tc)/StuttgartRLC+STO-3G(Se)/6-31G*(C,N,P,Cl)/6-31G(H) level. The spectral signature in the visible part of the spectrum is consistent with the presence of the monomeric Tc(II) instead of the dimeric Tc(I) compound.

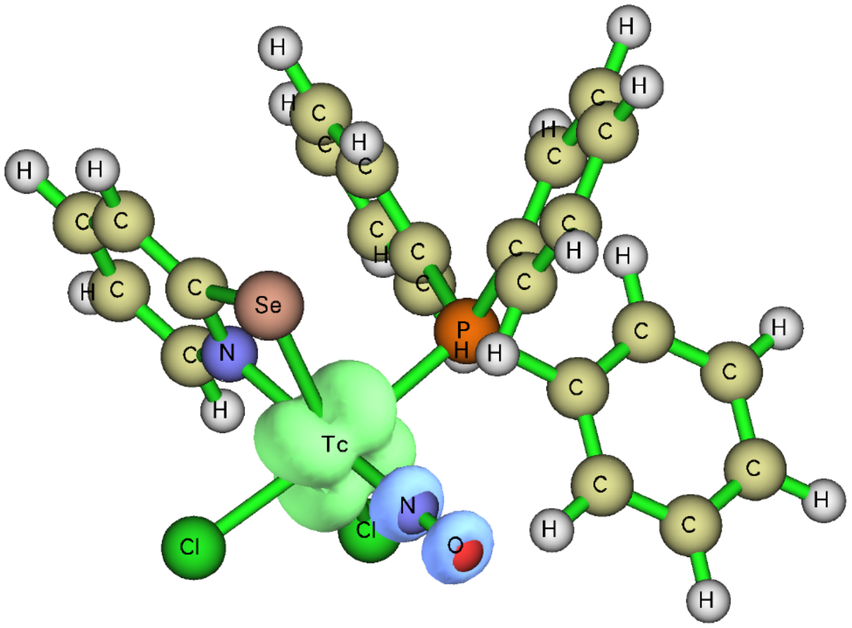

Figure S33. Spin density of [Tc(NO)Cl$_2$(PPh$_3$)(2-pySe)] at an isosurface level of 0.01. B3LYP-GD3B/StuttgartRSC(Tc)/StuttgartRLC+STO-3G(Se)/6-31G*(C,N,P,Cl)/6-31G(H) level.

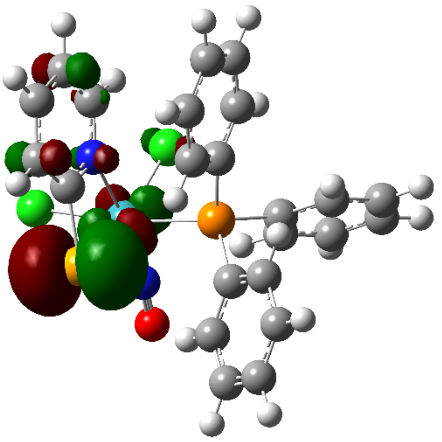

Figure S34. SOMO of [Tc(NO)Cl_2(PPh_3)(2-pySe)]. B3LYP-GD3B/StuttgartRSC(Tc)/StuttgartRLC+STO-3G(Se)/6-31G*(C,N,P,Cl)/6-31G(H) level.

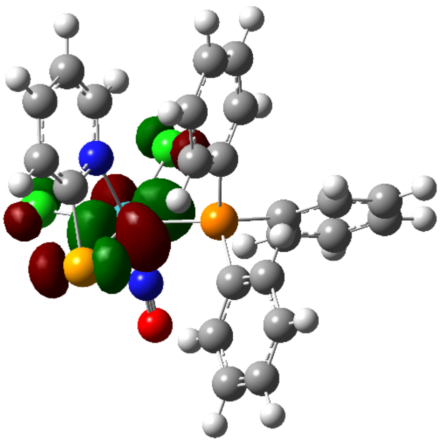

Figure S35. LUMO of [Tc(NO)Cl_2(PPh_3)(2-pySe)]. B3LYP-GD3B/StuttgartRSC(Tc)/StuttgartRLC+STO-3G(Se)/6-31G*(C,N,P,Cl)/6-31G(H) level.

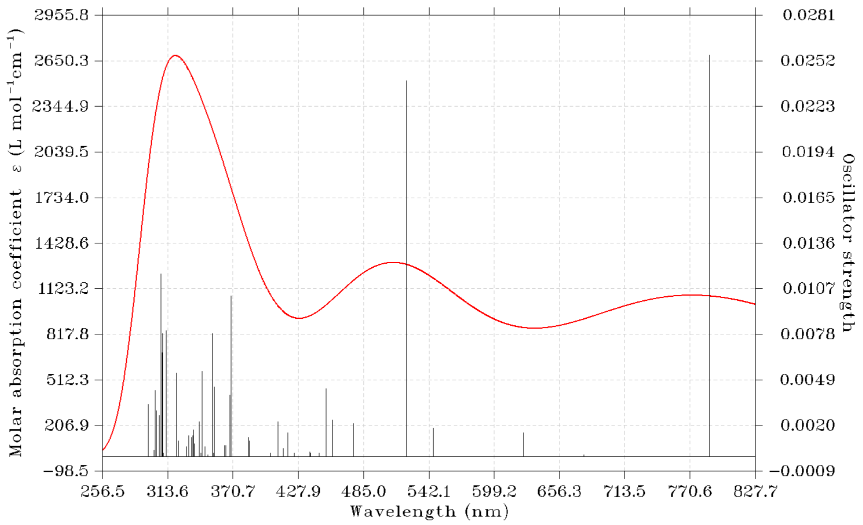

Figure S36. Simulated UV-Vis spectrum of [Tc(NO)Cl_2(PPh_3)(2-pySe)]; 50 transitions were considered. B3LYP-GD3B/StuttgartRSC(Tc)/StuttgartRLC+STO-3G(Se)/6-31G*(C,N,P,Cl)/6-31G(H) level.

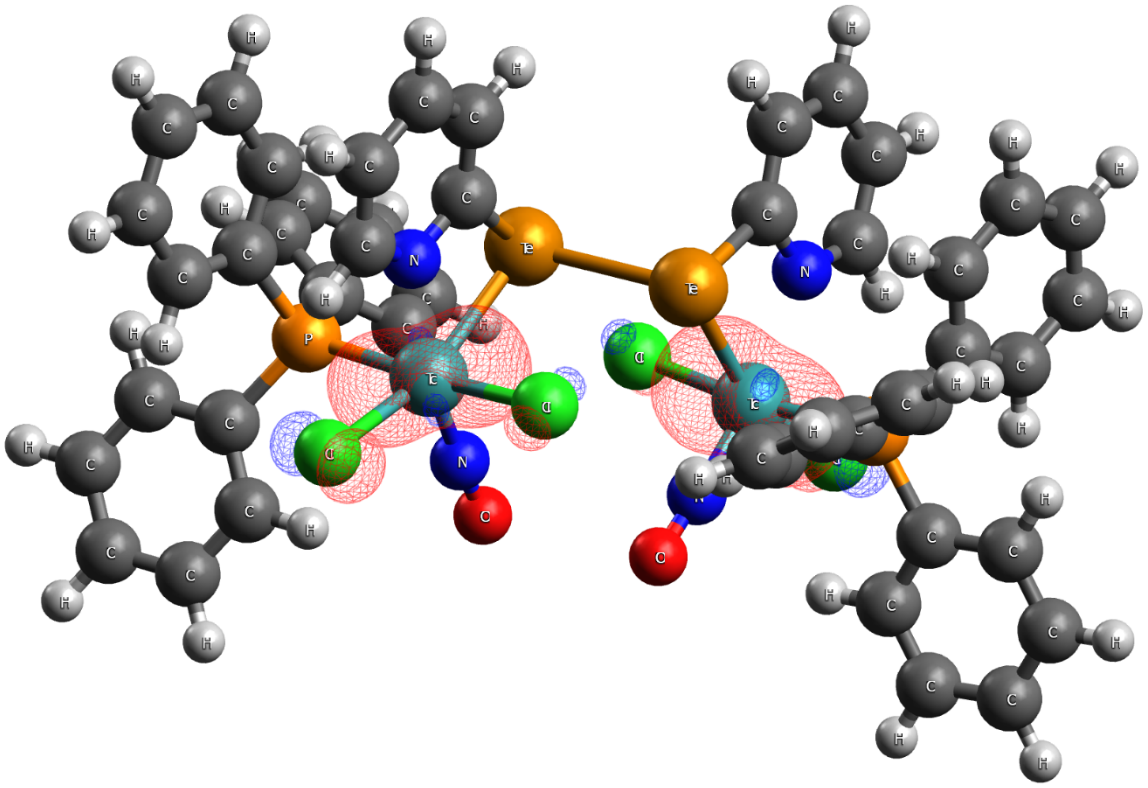

Figure S37. HOMO of $[\{Tc^{I}(NO)Cl_2(PPh_3)\}_2\{\mu_2\text{-}(2\text{-}pyTe)_2\}]$ at an isosurface value of 0.05. B3LYP-GD3B/StuttgartRSC(Tc)/StuttgartRLC+STO-3G(Te)/6-31G*(C,N,P,Cl)/6-31G(H) level.

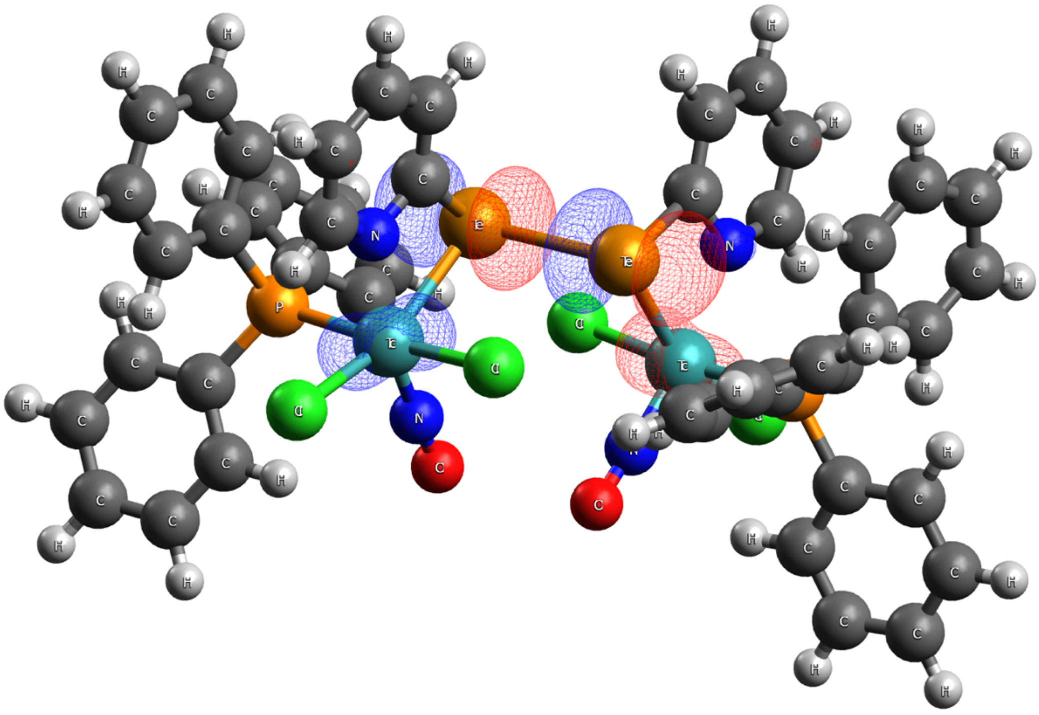

Figure S38. LUMO of $[\{Tc^{I}(NO)Cl_2(PPh_3)\}_2\{\mu_2\text{-}\{2\text{-}pyTe)_2\}]$ at an isosurface value of 0.05. B3LYP-GD3B/StuttgartRSC(Tc)/StuttgartRLC+STO-3G(Te)/6-31G*(C,N,P,Cl)/6-31G(H) level.

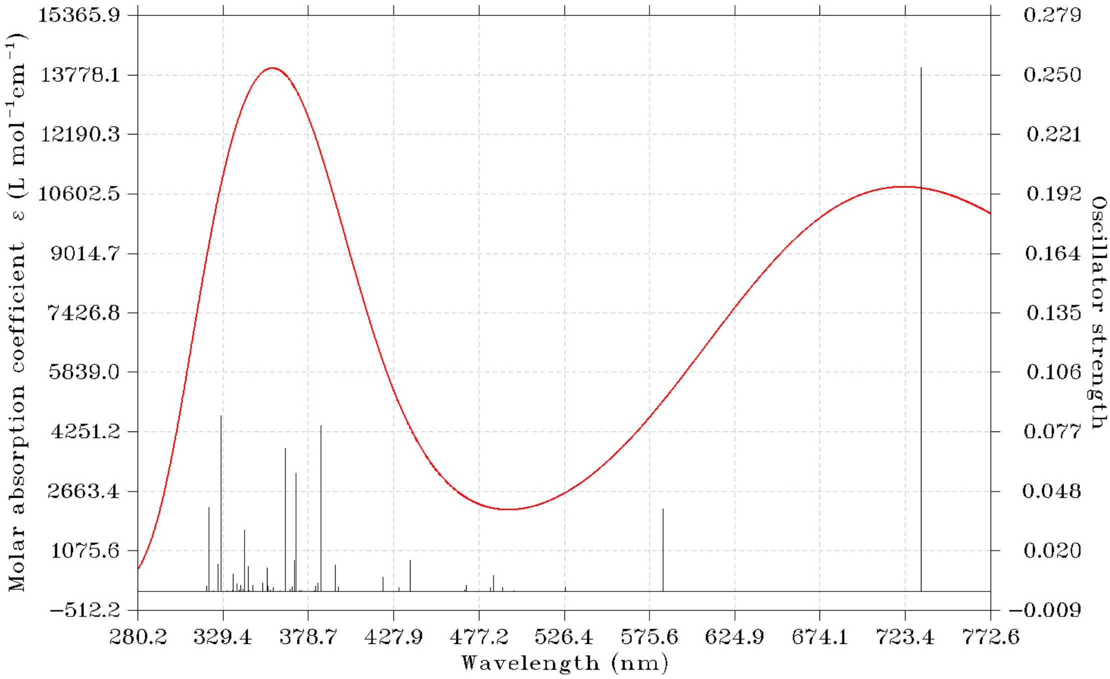

Figure S39. Simulated UV-Vis spectrum of [{Tc^{I}(NO)Cl_2(PPh_3)}$_2${μ_2-(2-pyTe)$_2$}]; 50 transitions were considered. B3LYP-GD3B/StuttgartRSC(Tc)/StuttgartRLC+STO-3G(S,Te,Te)/6-31G*(C,N,P,Cl)/6-31G(H) level.

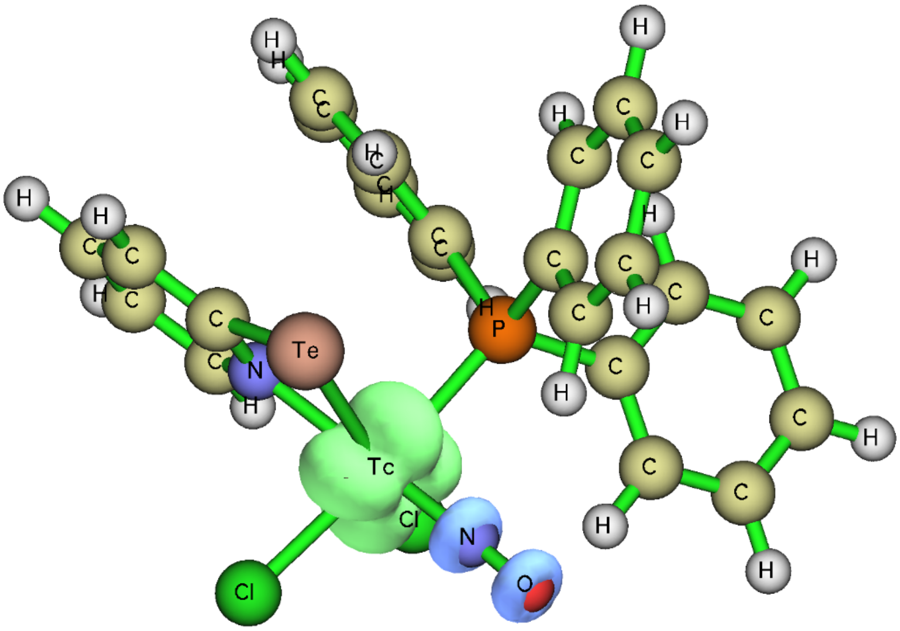

Figure S40. Spin density of [Tc(NO)Cl_2(PPh_3)(2-pyTe)] at an isosurface level of 0.01. B3LYP-GD3B/StuttgartRSC(Tc)/StuttgartRLC+STO-3G(Te)/6-31G*(C,N,P,Cl)/6-31G(H) level.

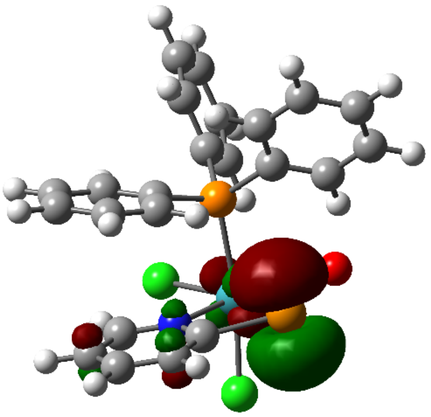

Figure S41. SOMO of [Tc(NO)Cl2(PPh3)(2-pyTe)]. B3LYP-GD3B/StuttgartRSC(Tc)/StuttgartRLC+STO-3G(Te)/6-31G*(C,N,P,Cl)/6-31G(H) level.

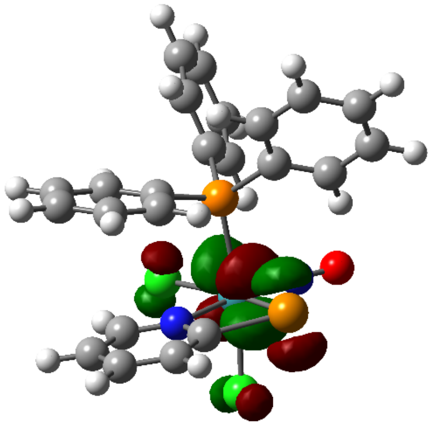

Figure S42. LUMO of [Tc(NO)Cl2(PPh3)(2-pyTe)]. B3LYP-GD3B/StuttgartRSC(Tc)/StuttgartRLC+STO-3G(Te)/6-31G*(C,N,P,Cl)/6-31G(H) level.

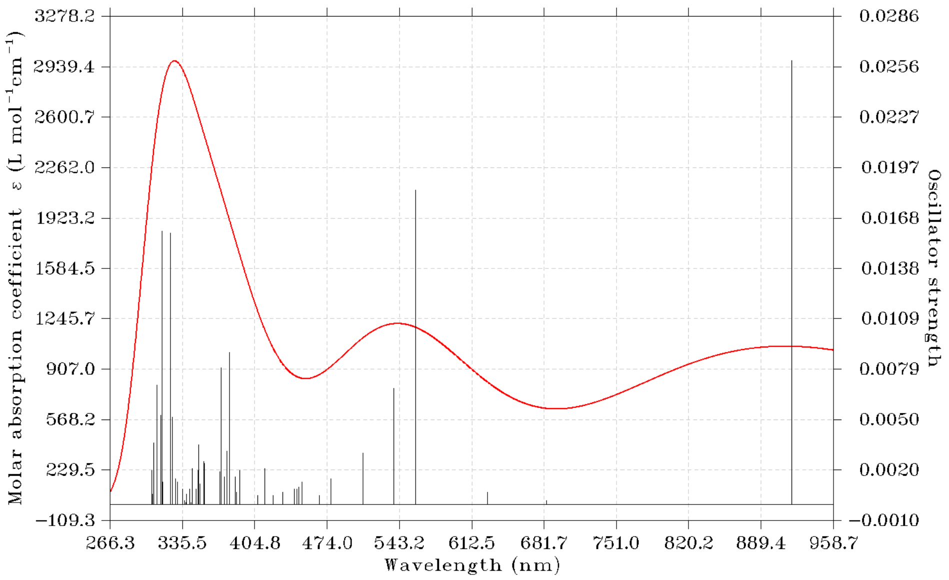

Figure S43. Simulated UV-Vis spectrum of [Tc(NO)Cl2(PPh3)(2-pyTe)]; 50 transitions were considered. B3LYP-GD3B/StuttgartRSC(Tc)/StuttgartRLC+STO-3G(Te)/6-31G*(C,N,P,Cl)/6-31G(H) level.

Table S10. Free energies Δ*G* obtained by the DFT calculations at different levels (gas-phase: standard conditions, B3LYP/StuttgartRSC(Tc)/StuttgartRLC+STO-3G(Te)/6-31G*(C,N,P,Cl)/6-31G(H) level; solvent: IEF-PCM for toluene at B3LYP-GD3B/StuttgartRSC(Tc)/StuttgartRLC+STO-3G(Te)/6-31G*(C,N,P,Cl)/6-31G(H) level & solvent with correction for rotational modes).

conditions		ΔG_{mono} [Hartree]	$2\Delta G_{mono}$ [Hartree]	ΔG_{dimer} [Hartree]	$\Delta\Delta G$ [Hartree]	$\Delta\Delta G$ [kJ/mol]
vacuum	S	-2425.2041	-4850.4081	-4850.3649	-0.0432	-114
	Se	-2424.3531	-4848.7062	-4848.6722	-0.0340	-89
	Te	-2423.0772	-4846.1544	-4846.1412	-0.0131	-34
solv. disp.	S	-2425.3848	-4850.7695	-4850.7543	-0.0153	-40
	Se	-2424.5362	-4849.0724	-4849.0681	-0.0043	-11
	Te	-2423.2649	-4846.5297	-4846.5479	0.0182	48
solv. disp. rot.	S	-2425.4132	-4850.8264	-4850.8116	-0.0148	-39
	Se	-2424.5645	-4849.1290	-4849.1241	-0.0049	-13
	Te	-2423.293	-4846.5860	-4846.6039	0.0179	47

Table S11. Highest occupied molecular orbital (HOMO or singly occupied molecular orbital; SOMO) and highest unoccupied molecular orbital (LUMO) energies and energy differences. Calculations with IEF-PCM for solvent toluene at B3LYP-GD3B/StuttgartRSC(Tc)/StuttgartRLC+STO-3G(Te)/6-31G*(C,N,P,Cl)/6-31G(H) level & solvent with correction for rotational modes).

		E_{HOMO} [eV]	E_{LUMO} [eV]	$\Delta E_{HOMO-LUMO}$ [eV]	$\Delta E_{HOMO-LUMO}$ [nm]
dimer	S	-5.187	-3.51	-1.677	739
	Se	-5.184	-3.481	-1.703	728
	Te	-5.218	-3.112	-2.106	589
monomer	S	-6.016	-2.151	-3.865	321
	Se	-5.904	-2.132	-3.772	329
	Te	-5.707	-2.087	-3.62	342

Table S12. ^{99}Tc NMR chemical shifts and shielding tensors calculated at B3P86/x2c-TZVPPall-s level based on geometries calculated with IEF-PCM for solvent toluene at B3LYP-GD3B/StuttgartRSC(Tc)/StuttgartRLC+STO-3G(Te)/6-31G*(C,N,P,Cl)/6-31G(H) level & solvent with correction for rotational modes).

Compound	$\delta_{exp.}$(^{99}Tc) [ppm]	Calc. ^{99}Tc shielding	$\delta_{calc.}$(^{99}Tc) [ppm]	$\delta_{calc.}$
[{Tc^{I}(NO)Cl$_2$(PPh$_3$)}$_2$(μ$_2$-pySSpy)]	Not observed	-3155.9	927	
[{Tc^{I}(NO)Cl$_2$(PPh$_3$)}$_2$(μ$_2$-pySeSepy)]	894	-3218.28	989	95
[{Tc^{I}(NO)Cl$_2$(PPh$_3$)}$_2$(μ$_2$-pyTeTepy)]	716	-2932.03	703	-13
TcO_4^- (reference)	0	-2229		

The delocalization of the unpair-electron into the organic pyridyl residue in the SOMO of the radicals ·EPy obtained from homolytic bond cleavage of free $(2\text{-pyE})_2$ increases in the order S > Se > Te, while a localization of the unpaired electron at the chalcogen becomes more probable, the heavier the chalcogen atom becomes; this is also evident from the respective spin-density distributions. Energetically, the homolytic bond cleavage is severely disfavored in all cases. Although it is surprisingly lowest for Te, the energy barrier is still very high and such reactivity should not be expected from free $(2\text{-pyE})_2$ without external stimulant lowering the energy barrier for this cleavage, i.e. in this case Tc(I) ions. Interestingly, technetium reduces $(2\text{-pyS})_2$ most efficiently, reversing the trend observed for the free dichalcogenides.

Table S13. Free energies ΔG obtained by the DFT calculations for the dissociation of $(2\text{-pyE})_2$ in toluene (IEF-PCM) at B3LYP-GD3B/StuttgartRSC(Tc)/StuttgartRLC+STO-3G(Te)/6-31G*(C,N,P,Cl)/6-31G(H) level.

	$\Delta G_{\cdot EPy}$ [Hartree]	$2\Delta G_{mono}$ [Hartree]	ΔG_{E2Py2} [Hartree]	$\Delta\Delta G$ [Hartree]	$\Delta\Delta G$ [kJ/mol]
**S**	-257.8122	-515.6244	-515.6999	0.0755	198
**Se**	-256.9729	-513.9457	-513.9978	0.0521	137
**Te**	-255.707	-511.4147	-511.466	0.0513	135

Table S14. Highest occupied molecular orbital (HOMO or singly occupied molecular orbital; SOMO) and highest unoccupied molecular orbital (LUMO) energies and energy differences for ·{2-pyE} and $(2\text{-pyE})_2$. Calculations with IEF-PCM for solvent toluene at B3LYP-GD3B/StuttgartRSC(Tc)/StuttgartRLC+STO-3G(Te)/6-31G*(C,N,P,Cl)/6-31G(H) level & solvent with correction for rotational modes).

		E_{HOMO} [eV]	E_{LUMO} [eV]	$\Delta E_{HOMO-LUMO}$ [eV]	$\Delta E_{HOMO-LUMO}$ [nm]
	S	-6.302	-1.255	-5.047	246
$(2\text{-pyE})_2$	Se	-6.058	-2.614	-3.444	360
	Te	-5.71	-2.655	-3.055	406
	S	-6.479	-0.91	-5.569	321
·{EPy}	Se	-6.346	-0.893	-5.453	329
	Te	-6.058	-0.852	-5.206	342

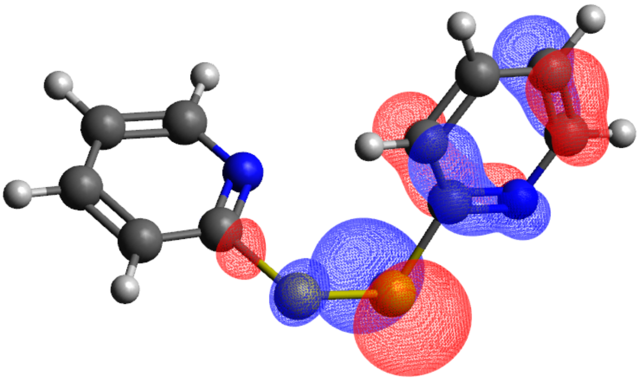

Figure S44. HOMO of $(2\text{-pyS})_2P$ at an isosurface value of 0.05. B3LYP-GD3B/StuttgartRSC(Tc)/StuttgartRLC+STO-3G(S)/6-31G*(C,N,P,Cl)/6-31G(H) level.

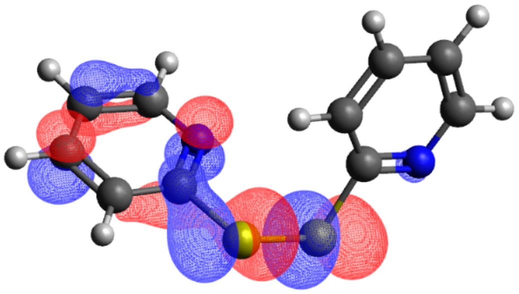

Figure S45. LUMO of $(2\text{-pyS})_2$ at an isosurface value of 0.05. B3LYP-GD3B/StuttgartRSC(Tc)/StuttgartRLC+STO-3G(S)/6-31G*(C,N,P,Cl)/6-31G(H) level.

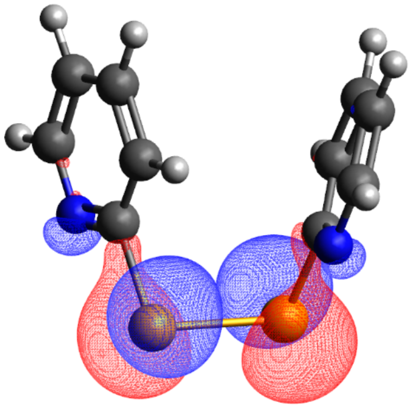

Figure S46. HOMO of $(2\text{-pySe})_2$ at an isosurface value of 0.05. B3LYP-GD3B/StuttgartRSC(Tc)/StuttgartRLC+STO-3G(Se)/6-31G*(C,N,P,Cl)/6-31G(H) level.

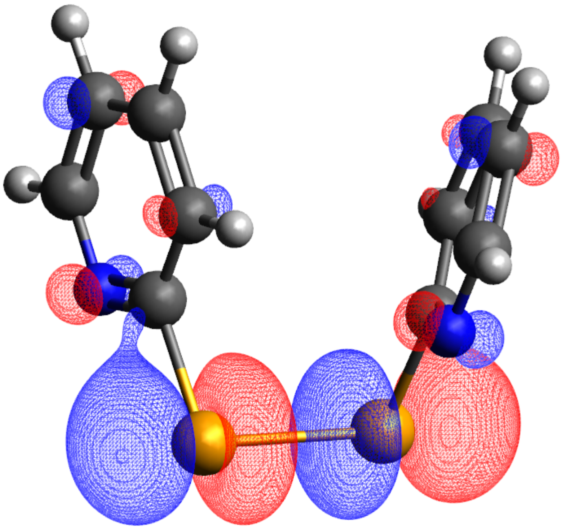

Figure S47. LUMO of $(2\text{-pyTe})_2$ at an isosurface value of 0.05. B3LYP-GD3B/StuttgartRSC(Tc)/StuttgartRLC+STO-3G(Se)/6-31G*(C,N,P,Cl)/6-31G(H) level.

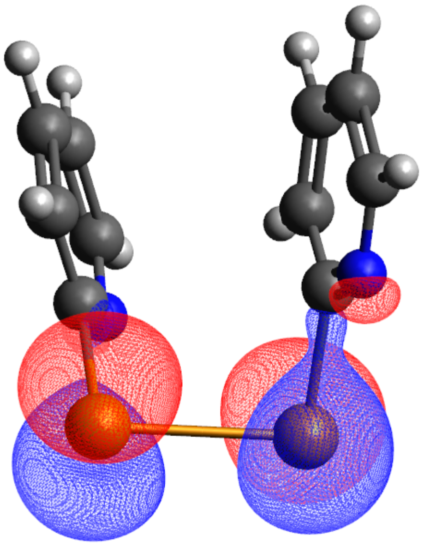

Figure S48. HOMO of (2-pyTe)$_2$ at an isosurface value of 0.05. B3LYP-GD3B/StuttgartRSC(Tc)/StuttgartRLC+STO-3G(Te)/6-31G*(C,N,P,Cl)/6-31G(H) level.

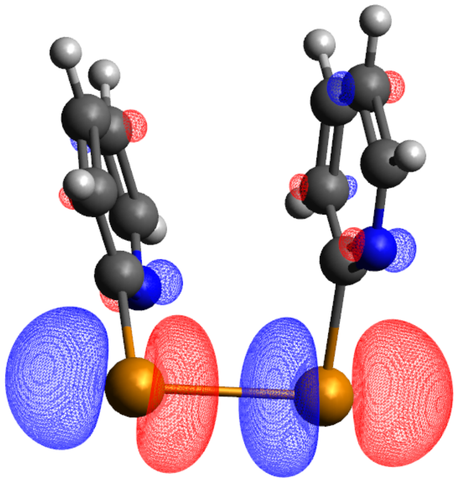

Figure S49. LUMO of (2-pyTe)$_2$ at an isosurface value of 0.05. B3LYP-GD3B/StuttgartRSC(Tc)/StuttgartRLC+STO-3G(Te)/6-31G*(C,N,P,Cl)/6-31G(H) level.

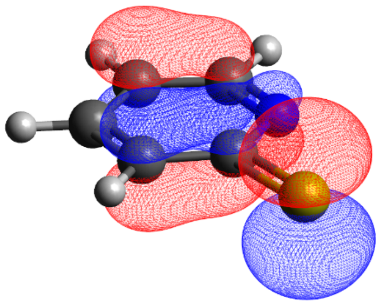

Figure S50. SOMO of ·{2-pyS} at an isosurface value of 0.05. U-B3LYP-GD3B/StuttgartRSC(Tc)/StuttgartRLC+STO-3G(S)/6-31G*(C,N,P,Cl)/6-31G(H) level.

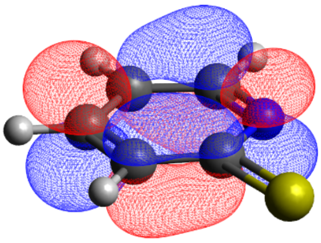

Figure S51. LUMO of ·{pyS} at an isosurface value of 0.05. U-B3LYP-GD3B/StuttgartRSC(Tc)/StuttgartRLC+STO-3G(S)/6-31G*(C,N,P,Cl)/6-31G(H) level.

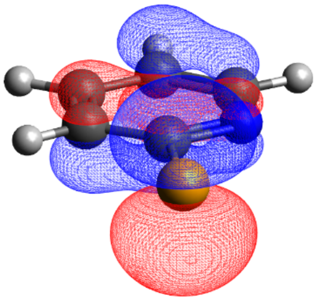

Figure S52. SOMO of ·{2-pySe} at an isosurface value of 0.05. U-B3LYP-GD3B/StuttgartRSC(Tc)/StuttgartRLC+STO-3G(Se)/6-31G*(C,N,P,Cl)/6-31G(H) level.

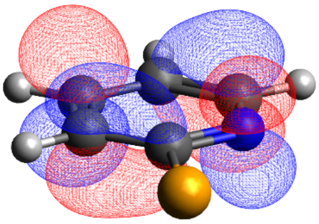

Figure S53. LUMO of ·{2-pySe} at an isosurface value of 0.05. U-B3LYP-GD3B/StuttgartRSC(Tc)/StuttgartRLC+STO-3G(Se)/6-31G*(C,N,P,Cl)/6-31G(H) level.

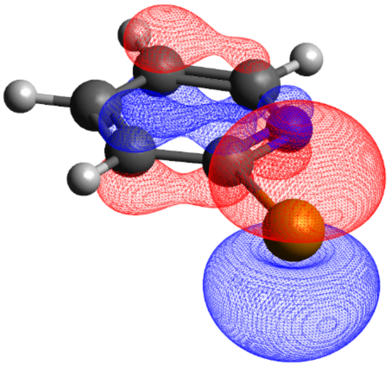

Figure S54. SOMO of ·{2-pyTe} at an isosurface value of 0.05. U-B3LYP-GD3B/StuttgartRSC(Tc)/StuttgartRLC+STO-3G(Te)/6-31G*(C,N,P,Cl)/6-31G(H) level.

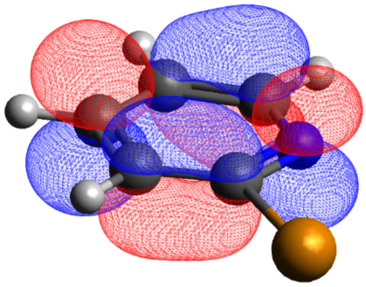

Figure S55. LUMO of ·{2-pyTe} at an isosurface value of 0.05. U-B3LYP-GD3B/StuttgartRSC(Tc)/StuttgartRLC+STO-3G(Te)/6-31G*(C,N,P,Cl)/6-31G(H) level.

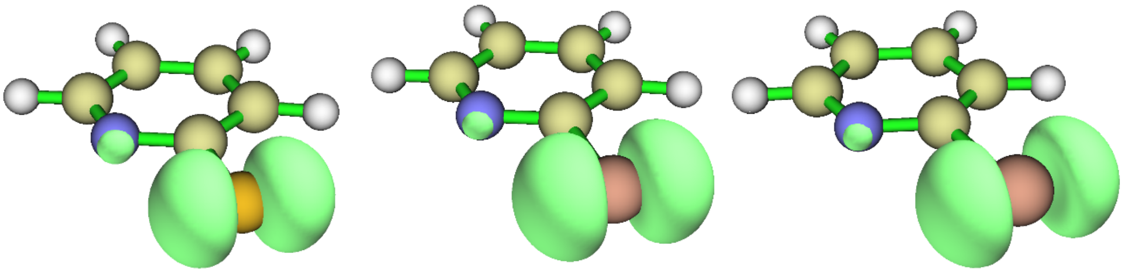

Figure S56. Spin densities of ·{2-pyE} (left to right; E = S, Se, Te) at an isosurface value of 0.01. U-B3LYP-GD3B/StuttgartRSC(Tc)/StuttgartRLC+STO-3G(S)/6-31G*(C,N,P,Cl)/6-31G(H) level.

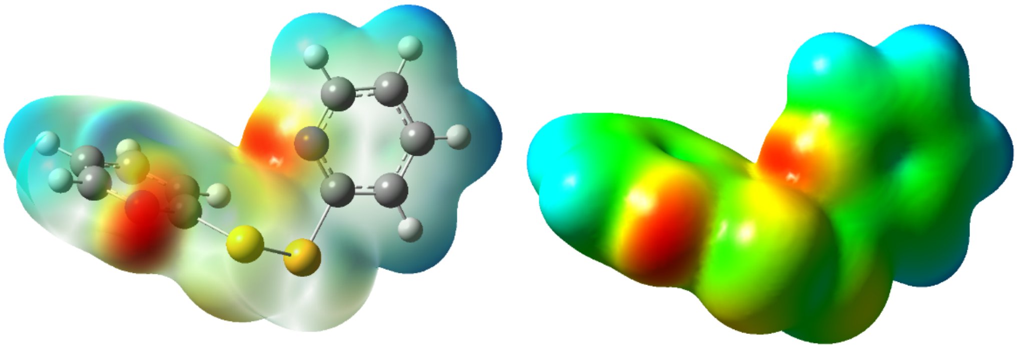

Figure S57. Electrostatic potential (ESP) mapping of $(2\text{-pyS})_2$ at an isosurface value of 0.004 (left: transparent mesh to highlight molecular orientation, right: untransparent mesh to highlight the values). Dark blue: corresponds to a surface potential of $8.314{\cdot}10^{-2}$, while green is 0 and red is $-8.314{\cdot}10^{-2}$. B3LYP-GD3B/StuttgartRSC(Tc)/StuttgartRLC+STO-3G(S)/6-31G*(C,N,P,Cl)/6-31G(H) level.

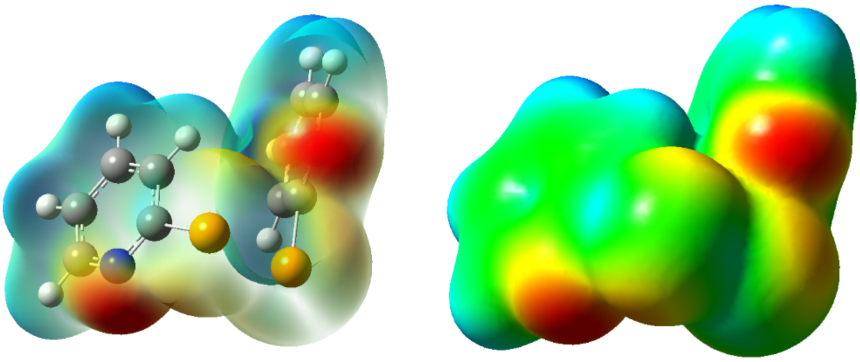

Figure S58. Electrostatic potential (ESP) mapping of (2-pySe)2 at an isosurface value of 0.004 (left: transparent mesh to highlight molecular orientation, right: untransparent mesh to highlight the values). Dark blue: corresponds to a surface potential of $8.314 \cdot 10^{-2}$, while green is 0 and red is $-8.314 \cdot 10^{-2}$. The values are normalized to those of free (2-pyS)2. B3LYP-GD3B/StuttgartRSC(Tc)/StuttgartRLC+STO-3G(Se)/6-31G*(C,N,P,Cl)/6-31G(H) level.

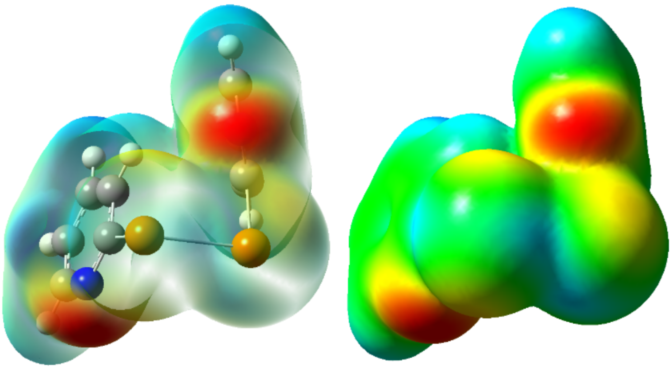

Figure S59. Electrostatic potential (ESP) mapping of (2-pyTe)2 at an isosurface value of 0.004 (left: transparent mesh to highlight molecular orientation, right: untransparent mesh to highlight the values). Dark blue: corresponds to a surface potential of $8.314 \cdot 10^{-2}$, while green is 0 and red is $-8.314 \cdot 10^{-2}$. The values are normalized to those of free (2-pyS)2. B3LYP-GD3B/StuttgartRSC(Tc)/StuttgartRLC+STO-3G(Te)/6-31G*(C,N,P,Cl)/6-31G(H) level.

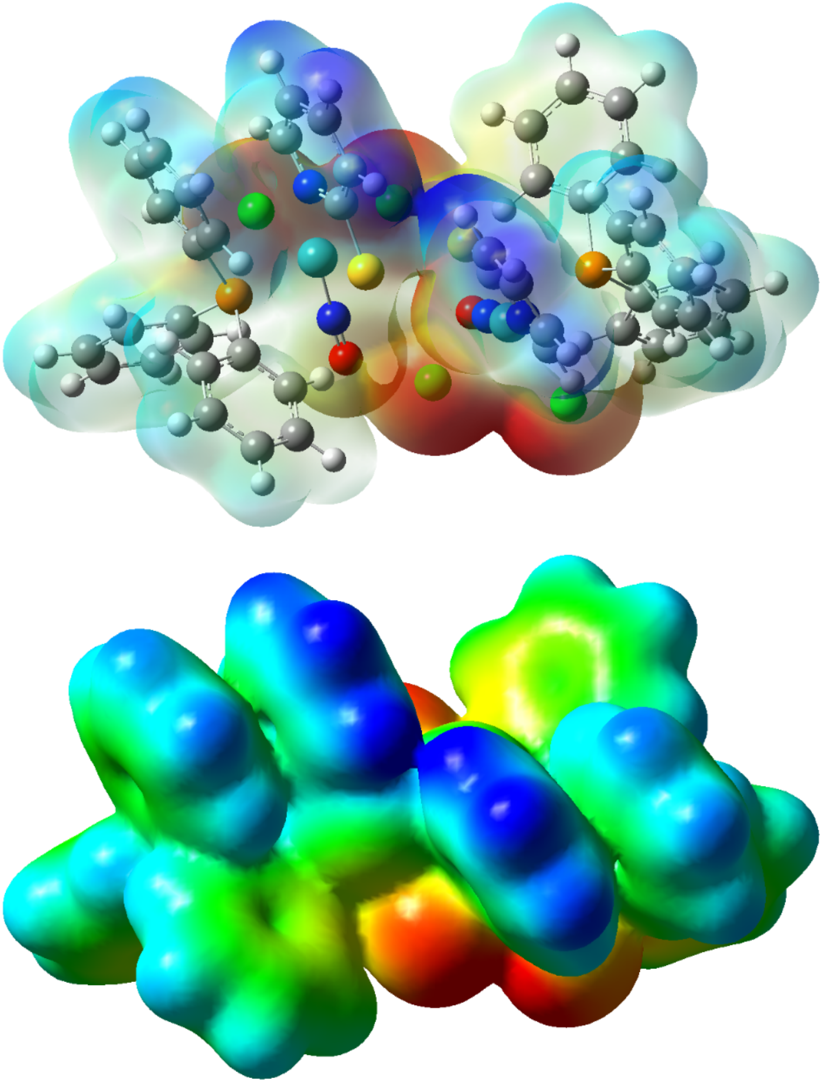

Figure S60. Electrostatic potential (ESP) mapping of [{$Tc^{I}(NO)Cl_2(PPh_3)$}$_2${μ_2-{2-pyS)$_2$}] at an isosurface value of 0.004 (left: transparent mesh to highlight molecular orientation, right: untransparent mesh to highlight the values). Dark blue: corresponds to a surface potential of $8.314 \cdot 10^{-2}$, while green is 0 and red is $-8.314 \cdot 10^{-2}$. B3LYP-GD3B/StuttgartRSC(Tc)/StuttgartRLC+STO-3G(S)/6-31G*(C,N,P,Cl)/6-31G(H) level. The position of the σ-hole *trans* to the pyridyl substituent is occupied by Cl and therefore obscured, while the position of σ-hole opposite to the chalcogen-chalcogen bond is obscured by the bulk of triphenyl phosphine.

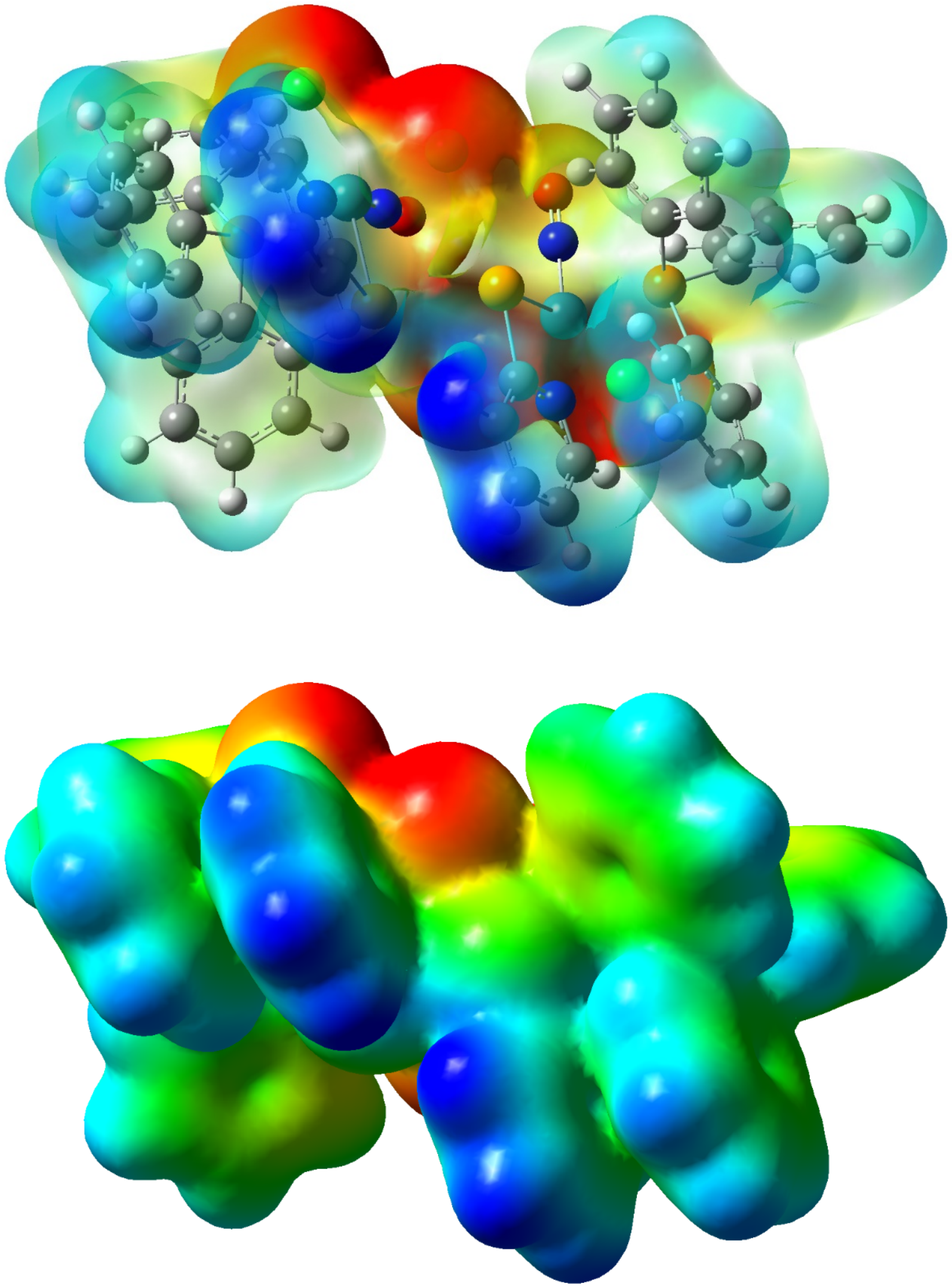

Figure S61. Electrostatic potential (ESP) mapping of [{$Tc^{I}(NO)Cl_2(PPh_3)$}$_2${μ_2-{2-pySe)$_2$}] at an isosurface value of 0.004 (top: transparent mesh to highlight molecular orientation, bottom: untransparent mesh to highlight the values). Dark blue: corresponds to a surface potential of $8.314 \cdot 10^{-2}$, while green is 0 and red is $-8.314 \cdot 10^{-2}$. The values are normalized to those of free (2-pyS)$_2$. B3LYP-GD3B/StuttgartRSC(Tc)/StuttgartRLC+STO-3G(Se)/6-31G*(C,N,P,Cl)/6-31G(H) level. The position of the σ-hole *trans* to the pyridyl substituent is occupied by Cl and therefore obscured, while the position of σ-hole opposite to the chalcogen-chalcogen bond is obscured by the bulk of triphenyl phosphine.

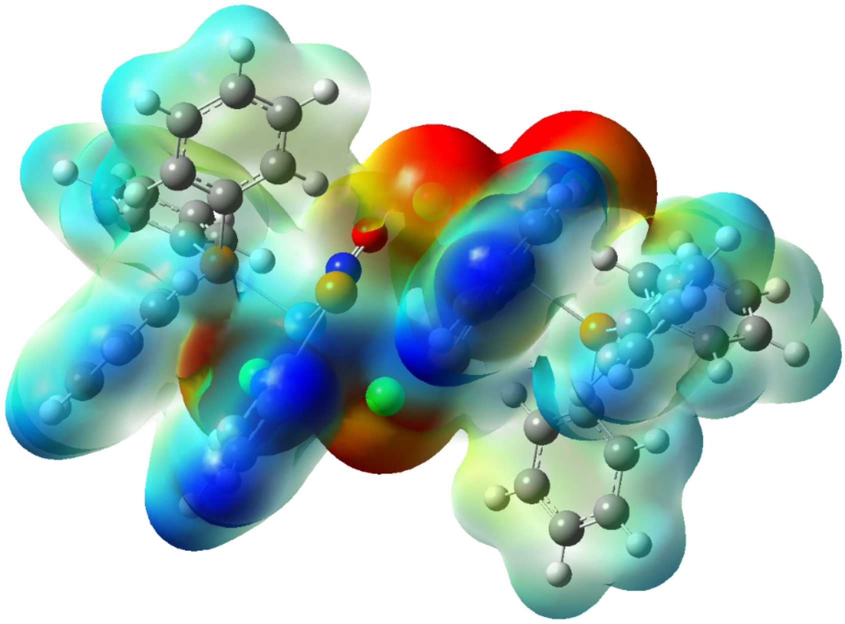

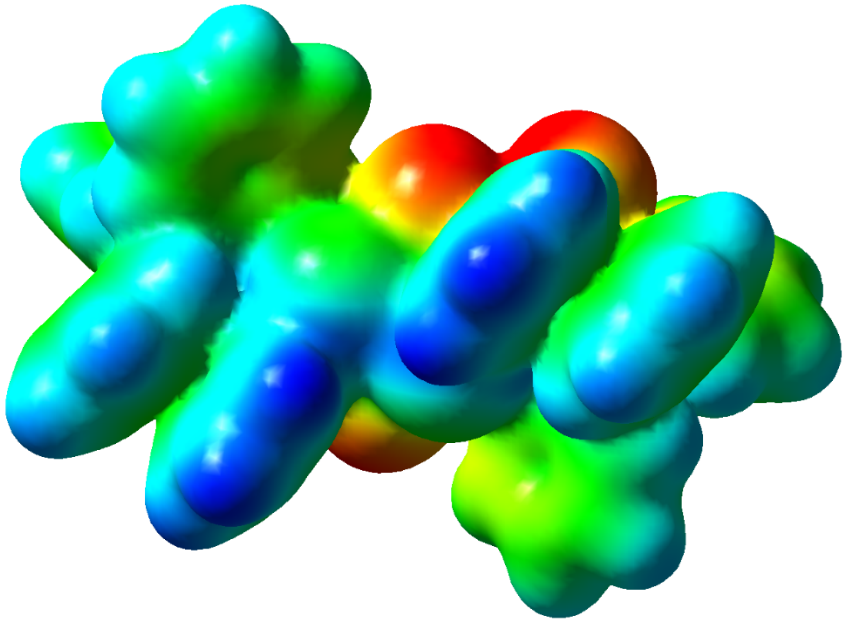

Figure S62. Electrostatic potential (ESP) mapping of [{Tc^{I}(NO)Cl$_2$(PPh$_3$)}$_2${μ$_2$-(2-pyTe)$_2$}] at an isosurface value of 0.004 (left: transparent mesh to highlight molecular orientation, right: untransparent mesh to highlight the values). Dark blue: corresponds to a surface potential of 8.314·10^{-2}, while green is 0 and red is -8.314·10^{-2}. The values are normalized to those of free (2-pyS)$_2$. B3LYP-GD3B/StuttgartRSC(Tc)/StuttgartRLC+STO-3G(Te)/6-31G*(C,N,P,Cl)/6-31G(H) level.
