# Supplementary material for: [Tc(NO)Cl2(PPh3)2(CH3CN)] and Its Reactions with 2,2′-Dipyridyl Dichalcogenides
Source: Molecules. 2025 Feb 8;30(4):793. doi: 10.3390/molecules30040793 (PMC11858252; doi:10.3390/molecules30040793)

# checkCIF/PLATON report

Structure factors have been supplied for datablock(s) tcnoop2ua, tcnose2ua, tcnosncl2pua, tcnote2pyua

THIS REPORT IS FOR GUIDANCE ONLY. IF USED AS PART OF A REVIEW PROCEDURE FOR PUBLICATION, IT SHOULD NOT REPLACE THE EXPERTISE OF AN EXPERIENCED CRYSTALLOGRAPHIC REFEREE.

No syntax errors found. CIF dictionary Interpreting this report

# Datablock: tcnoop2ua

| | | | |
|---|---|---|---|
| Bond precision: | C-C = 0.0113 A | | Wavelength=0.71073 |
| Cell: | a=12.5998(8) | b=14.1927(11) | c=44.238(3) |
| | alpha=90 | beta=97.499(2) | gamma=90 |
| Temperature: | 258 K | | |

| | Calculated | Reported |
|---|---|---|
| Volume | 7843.2(9) | 7843.3(10) |
| Space group | P 21/n | P 1 21/n 1 |
| Hall group | -P 2yn | -P 2yn |
| Moiety formula | C36 H30 Cl3 N O3 P2 Tc [+ solvent] | C36 H30 Cl3 N O3 P2 Tc, 0.5[C7H8] |
| Sum formula | C36 H30 Cl3 N O3 P2 Tc [+ solvent] | C39.50 H34 Cl3 N O3 P2 Tc |
| Mr | 791.81 | 836.97 |
| Dx,g cm-3 | 1.341 | 1.418 |
| Z | 8 | 8 |
| Mu (mm-1) | 0.686 | 0.690 |
| F000 | 3208.0 | 3408.0 |
| F000' | 3202.59 | |
| h,k,lmax | 15,16,52 | 15,16,52 |
| Nref | 14038 | 14016 |
| Tmin,Tmax | 0.959,0.966 | 0.670,0.745 |
| Tmin' | 0.871 | |

Correction method= # Reported T Limits: Tmin=0.670 Tmax=0.745
AbsCorr = EMPIRICAL

Data completeness= 0.998 Theta(max)= 25.152

R(reflections)= 0.0709( 10800) wR2(reflections)= 0.1513( 14016)

S = 1.077 Npar= 769

---

The following ALERTS were generated. Each ALERT has the format
**test-name_ALERT_alert-type_alert-level.**
Click on the hyperlinks for more details of the test.

---

### Alert level B

```
PLAT230_ALERT_2_B Hirshfeld Test Diff for    O20      --N20      .        9.3 s.u.
```

**Author Response: The NO unit is part of a badly resolved NO/Cl disorder**
**The NO unit is part of a badly resolved NO/Cl disorder**

---

### Alert level C

```
PLAT232_ALERT_2_C Hirshfeld Test Diff (M-X)  Tc2      --N20      .        8.0 s.u.
PLAT234_ALERT_4_C Large Hirshfeld Difference C13      --C14      .       0.18 Ang.
PLAT241_ALERT_2_C High   'MainMol' Ueq as Compared to Neighbors of        C21 Check
PLAT241_ALERT_2_C High   'MainMol' Ueq as Compared to Neighbors of        C45 Check
PLAT241_ALERT_2_C High   'MainMol' Ueq as Compared to Neighbors of        C51 Check
PLAT241_ALERT_2_C High   'MainMol' Ueq as Compared to Neighbors of        C65 Check
PLAT241_ALERT_2_C High   'MainMol' Ueq as Compared to Neighbors of        C66 Check
PLAT241_ALERT_2_C High   'MainMol' Ueq as Compared to Neighbors of        C82 Check
PLAT241_ALERT_2_C High   'MainMol' Ueq as Compared to Neighbors of        C83 Check
PLAT241_ALERT_2_C High   'MainMol' Ueq as Compared to Neighbors of       C105 Check
PLAT242_ALERT_2_C Low    'MainMol' Ueq as Compared to Neighbors of        Tc1 Check
PLAT242_ALERT_2_C Low    'MainMol' Ueq as Compared to Neighbors of         P1 Check
PLAT242_ALERT_2_C Low    'MainMol' Ueq as Compared to Neighbors of         P2 Check
PLAT242_ALERT_2_C Low    'MainMol' Ueq as Compared to Neighbors of        N10 Check
PLAT242_ALERT_2_C Low    'MainMol' Ueq as Compared to Neighbors of        C41 Check
PLAT242_ALERT_2_C Low    'MainMol' Ueq as Compared to Neighbors of        Tc2 Check
PLAT242_ALERT_2_C Low    'MainMol' Ueq as Compared to Neighbors of        C84 Check
PLAT331_ALERT_2_C Small Aver Phenyl C-C Dist C21      --C26      .       1.37 Ang.
PLAT331_ALERT_2_C Small Aver Phenyl C-C Dist C61      --C66      .       1.37 Ang.
PLAT331_ALERT_2_C Small Aver Phenyl C-C Dist C71      --C76      .       1.37 Ang.
PLAT331_ALERT_2_C Small Aver Phenyl C-C Dist C81      --C86      .       1.37 Ang.
PLAT331_ALERT_2_C Small Aver Phenyl C-C Dist C101     --C106     .       1.37 Ang.
PLAT342_ALERT_3_C Low Bond Precision on  C-C Bonds ..............    0.01132 Ang.
PLAT906_ALERT_3_C Large K Value in the Analysis of Variance ......      4.990 Check
PLAT906_ALERT_3_C Large K Value in the Analysis of Variance ......      2.193 Check
PLAT910_ALERT_3_C Missing # of FCF Reflection(s) Below Theta(Min).          6 Note
               -1  0  1,   1  0  1,   0  1  1,   0  0  2,   0  1  2,   0  0  4,
PLAT911_ALERT_3_C Missing FCF Refl Between Thmin & STh/L=    0.598         14 Report
                1  2  0,   1  1  1,   1  7  1, -15  1  3,  -3  6  3,   0  0  6,
                0  3 12, -14  6 12,  -6  6 13,  13  6 13,   5 15 15,  -1  1 18,
               10  6 30, -11  8 31,
PLAT971_ALERT_2_C Check Calcd Resid. Dens.  0.12Ang From C21           1.53 eA-3
```

---

### Alert level G

```
FORMU01_ALERT_2_G  There is a discrepancy between the atom counts in the
            _chemical_formula_sum and the formula from the _atom_site* data.
            Atom count from _chemical_formula_sum:C39.5 H34 Cl3 N1 O3 P2 Tc1
            Atom count from the _atom_site data:  C36 H30 Cl3 N1 O3 P2 Tc1
CELLZ01_ALERT_1_G Difference between formula and atom_site contents detected.
CELLZ01_ALERT_1_G ALERT: Large difference may be due to a
            symmetry error - see SYMMG tests
           From the CIF: _cell_formula_units_Z    8
           From the CIF: _chemical_formula_sum  C39.50 H34 Cl3 N O3 P2 Tc
           TEST: Compare cell contents of formula and atom_site data

           atom    Z*formula  cif sites diff
           C        316.00    288.00   28.00
           H        272.00    240.00   32.00
           Cl        24.00     24.00    0.00
           N          8.00      8.00    0.00
           O         24.00     24.00    0.00
           P         16.00     16.00    0.00
           Tc         8.00      8.00    0.00
PLAT002_ALERT_2_G Number of Distance or Angle Restraints on AtSite         12 Note
PLAT041_ALERT_1_G Calc. and Reported SumFormula    Strings  Differ     Please Check
              Calc: C36 H30 Cl3 N O3 P2 Tc
              Rep.: C39.50 H34 Cl3 N O3 P2 Tc
PLAT042_ALERT_1_G Calc. and Reported MoietyFormula Strings  Differ     Please Check
              Calc: C36 H30 Cl3 N O3 P2 Tc
              Rep.: C36 H30 Cl3 N O3 P2 Tc, 0.5[C7H8]
PLAT083_ALERT_2_G SHELXL Second Parameter in WGHT  Unusually Large      32.12 Why ?
PLAT171_ALERT_4_G The CIF-Embedded .res File Contains EADP Records          2 Report
PLAT172_ALERT_4_G The CIF-Embedded .res File Contains DFIX Records          3 Report
PLAT176_ALERT_4_G The CIF-Embedded .res File Contains SADI Records          3 Report
PLAT232_ALERT_2_G Hirshfeld Test Diff (M-X)  Tc2      --Cl12     .       30.3 s.u.
PLAT606_ALERT_4_G Solvent Accessible VOID(S) in Structure ........          ! Info
PLAT860_ALERT_3_G Number of Least-Squares Restraints ............          25 Note
PLAT868_ALERT_4_G ALERTS Due to the Use of _smtbx_masks Suppressed          ! Info
PLAT909_ALERT_3_G Percentage of I>2sig(I) Data at Theta(Max) Still        47% Note
PLAT933_ALERT_2_G Number of HKL-OMIT Records in Embedded .res File          8 Note
               -6  6 13,  -3  6  3,  -1  1 18,   0  0  6,   0  3 12,   1  1  1,
                1  2  0,   1  7  1,
PLAT969_ALERT_5_G The 'Henn et al.' R-Factor-gap value ..........      5.032 Note
              Predicted wR2: Based on SigI**2  3.01 or SHELX Weight 14.05
PLAT978_ALERT_2_G Number C-C Bonds with Positive Residual Density.          2 Info
```

---

```
   0 ALERT level A = Most likely a serious problem - resolve or explain
   1 ALERT level B = A potentially serious problem, consider carefully
  28 ALERT level C = Check. Ensure it is not caused by an omission or oversight
  18 ALERT level G = General information/check it is not something unexpected

   4 ALERT type 1 CIF construction/syntax error, inconsistent or missing data
  29 ALERT type 2 Indicator that the structure model may be wrong or deficient
   7 ALERT type 3 Indicator that the structure quality may be low
   6 ALERT type 4 Improvement, methodology, query or suggestion
   1 ALERT type 5 Informative message, check
```

---

# Datablock: tcnote2pyua

Bond precision: C-C = 0.0090 A Wavelength=0.71073

| | | | |
|---|---|---|---|
| Cell: | a=15.7206(14) | b=23.827(2) | c=33.806(4) |
| | alpha=90 | beta=90 | gamma=90 |
| Temperature: | 275 K | | |

| | Calculated | Reported |
|---|---|---|
| Volume | 12663(2) | 12663(2) |
| Space group | I b c a | I b c a |
| Hall group | -I 2b 2c | -I 2b 2c |
| Moiety formula | C46 H38 Cl4 N4 O2 P2 Tc2 Te2, C H2 Cl2 [+ solvent] | C46 H38 Cl4 N4 O2 P2 Tc2 Te2, C H2 Cl2, 2[C7H8] |
| Sum formula | C47 H40 Cl6 N4 O2 P2 Tc2 Te2 [+ solvent] | C61 H56 Cl6 N4 O2 P2 Tc2 Te2 |
| Mr | 1420.49 | 1602.93 |
| Dx,g cm-3 | 1.490 | 1.682 |
| Z | 8 | 8 |
| Mu (mm-1) | 1.678 | 1.689 |
| F000 | 5504.0 | 6304.0 |
| F000' | 5482.42 | |
| h,k,lmax | 19,29,42 | 19,29,42 |
| Nref | 6498 | 6489 |
| Tmin,Tmax | 0.904,0.919 | 0.618,0.745 |
| Tmin' | 0.509 | |

Correction method= # Reported T Limits: Tmin=0.618 Tmax=0.745
AbsCorr = MULTI-SCAN

Data completeness= 0.999 Theta(max)= 26.381

R(reflections)= 0.0361( 5136) wR2(reflections)= 0.0984( 6489)

S = 1.108 Npar= 294

The following ALERTS were generated. Each ALERT has the format
**test-name_ALERT_alert-type_alert-level**.
Click on the hyperlinks for more details of the test.

## Alert level C

ABSTY02_ALERT_1_C An _exptl_absorpt_correction_type has been given without
a literature citation. This should be contained in the
_exptl_absorpt_process_details field.
Absorption correction given as multi-scan

CHEMW01_ALERT_1_C The difference between the given and expected weight for

```
            compound is greater 1 mass unit. Check that all hydrogen
            atoms have been taken into account.
PLAT220_ALERT_2_C NonSolvent   Resd 1  C   Ueq(max)/Ueq(min) Range        3.1 Ratio
PLAT260_ALERT_2_C Large Average Ueq of Residue Including      Cl41      0.126 Check
PLAT342_ALERT_3_C Low Bond Precision on  C-C Bonds ..............    0.00895 Ang.
PLAT905_ALERT_3_C Negative K value in the Analysis of Variance ...     -2.863 Report
```

---

### Alert level G

```
FORMU01_ALERT_2_G  There is a discrepancy between the atom counts in the
            _chemical_formula_sum and the formula from the _atom_site* data.
            Atom count from _chemical_formula_sum:C61 H56 Cl6 N4 O2 P2 Tc2 Te2
            Atom count from the _atom_site data:  C47 H40 Cl6 N4 O2 P2 Tc2 Te2
CELLZ01_ALERT_1_G Difference between formula and atom_site contents detected.
CELLZ01_ALERT_1_G ALERT: Large difference may be due to a
            symmetry error - see SYMMG tests
           From the CIF: _cell_formula_units_Z    8
           From the CIF: _chemical_formula_sum  C61 H56 Cl6 N4 O2 P2 Tc2 Te2
           TEST: Compare cell contents of formula and atom_site data

           atom    Z*formula  cif sites diff
           C        488.00    376.00  112.00
           H        448.00    320.00  128.00
           Cl        48.00     48.00    0.00
           N         32.00     32.00    0.00
           O         16.00     16.00    0.00
           P         16.00     16.00    0.00
           Tc        16.00     16.00    0.00
           Te        16.00     16.00    0.00
PLAT041_ALERT_1_G Calc. and Reported SumFormula    Strings  Differ     Please Check
             Calc: C47 H40 Cl6 N4 O2 P2 Tc2 Te2
             Rep.: C61 H56 Cl6 N4 O2 P2 Tc2 Te2
PLAT042_ALERT_1_G Calc. and Reported MoietyFormula Strings  Differ     Please Check
             Calc: C46 H38 Cl4 N4 O2 P2 Tc2 Te2, C H2 Cl2
             Rep.: C46 H38 Cl4 N4 O2 P2 Tc2 Te2, C H2 Cl2, 2[C7H8
                   ]
PLAT083_ALERT_2_G SHELXL Second Parameter in WGHT  Unusually Large      64.93 Why ?
PLAT232_ALERT_2_G Hirshfeld Test Diff (M-X)  Te1      --Tc1      .        6.0 s.u.
PLAT299_ALERT_4_G Atom Site Occupancy Constrained at ............        0.5 Check
             H40A    H40B
PLAT606_ALERT_4_G Solvent Accessible VOID(S) in Structure ........          ! Info
PLAT868_ALERT_4_G ALERTS Due to the Use of _smtbx_masks Suppressed          ! Info
PLAT910_ALERT_3_G Missing # of FCF Reflection(s) Below Theta(Min).          4 Note
               0  2  0,   0  0  2,   1  1  2,   0  2  2,
PLAT912_ALERT_4_G Missing # of FCF Reflections Above STh/L=  0.600          3 Note
PLAT913_ALERT_3_G Missing # of Very Strong Reflections in FCF ....          1 Note
               0  0  2,
PLAT933_ALERT_2_G Number of HKL-OMIT Records in Embedded .res File          1 Note
               1  1  2,
PLAT969_ALERT_5_G The 'Henn et al.' R-Factor-gap value ..........      3.876 Note
             Predicted wR2: Based on SigI**2  2.54 or SHELX Weight  8.88
PLAT978_ALERT_2_G Number C-C Bonds with Positive Residual Density.          0 Info
```

---

0 **ALERT level A** = Most likely a serious problem - resolve or explain
0 **ALERT level B** = A potentially serious problem, consider carefully
6 **ALERT level C** = Check. Ensure it is not caused by an omission or oversight

16 **ALERT level G** = General information/check it is not something unexpected

6 ALERT type 1 CIF construction/syntax error, inconsistent or missing data
7 ALERT type 2 Indicator that the structure model may be wrong or deficient
4 ALERT type 3 Indicator that the structure quality may be low
4 ALERT type 4 Improvement, methodology, query or suggestion
1 ALERT type 5 Informative message, check

---

# Datablock: tcnose2ua

---

Bond precision: C-C = 0.0086 A Wavelength=0.71073

| | | | |
|---|---|---|---|
| Cell: | a=12.9663(4) | b=28.6368(10) | c=16.0265(6) |
| | alpha=90 | beta=107.384(1) | gamma=90 |
| Temperature: | 107 K | | |

| | Calculated | Reported |
|---|---|---|
| Volume | 5679.0(3) | 5679.0(3) |
| Space group | P 21/c | P 1 21/c 1 |
| Hall group | -P 2ybc | -P 2ybc |
| Moiety formula | C46 H38 Cl4 N4 O2 P2 Se2 Tc2, C7 H8 [+ solvent] | C46 H38 Cl4 N4 O2 P2 Se2 Tc2, C7 H8, 0.5[C7H8] |
| Sum formula | C53 H46 Cl4 N4 O2 P2 Se2 Tc2 [+ solvent] | C56.50 H50 Cl4 N4 O2 P2 Se2 Tc2 |
| Mr | 1330.42 | 1374.66 |
| Dx,g cm-3 | 1.556 | 1.608 |
| Z | 4 | 4 |
| Mu (mm-1) | 2.055 | 2.058 |
| F000 | 2640.0 | 2740.0 |
| F000' | 2631.53 | |
| h,k,lmax | 16,35,20 | 16,35,20 |
| Nref | 11632 | 11622 |
| Tmin,Tmax | 0.884,0.902 | 0.642,0.745 |
| Tmin' | 0.598 | |

Correction method= # Reported T Limits: Tmin=0.642 Tmax=0.745
AbsCorr = MULTI-SCAN

Data completeness= 0.999 Theta(max)= 26.388

R(reflections)= 0.0437( 9012) wR2(reflections)= 0.1232( 11622)

S = 1.176 Npar= 623

The following ALERTS were generated. Each ALERT has the format
**test-name_ALERT_alert-type_alert-level.**
Click on the hyperlinks for more details of the test.

## Alert level C

```
ABSTY02_ALERT_1_C  An _exptl_absorpt_correction_type has been given without
            a literature citation. This should be contained in the
            _exptl_absorpt_process_details field.
            Absorption correction given as multi-scan
CHEMW01_ALERT_1_C  The difference between the given and expected weight for
            compound is greater 1 mass unit. Check that all hydrogen
            atoms have been taken into account.
PLAT250_ALERT_2_C Large U3/U1 Ratio for <U(i,j)> Tensor(Resd    2)        2.4 Note
PLAT342_ALERT_3_C Low Bond Precision on  C-C Bonds ...............    0.00865 Ang.
PLAT905_ALERT_3_C Negative K value in the Analysis of Variance ...     -1.845 Report
```

## Alert level G

```
FORMU01_ALERT_2_G  There is a discrepancy between the atom counts in the
            _chemical_formula_sum and the formula from the _atom_site* data.
            Atom count from _chemical_formula_sum:C56.5 H50 Cl4 N4 O2 P2 Se2 Tc2
            Atom count from the _atom_site data:  C53 H46 Cl4 N4 O2 P2 Se2 Tc2
CELLZ01_ALERT_1_G Difference between formula and atom_site contents detected.
CELLZ01_ALERT_1_G ALERT: Large difference may be due to a
            symmetry error - see SYMMG tests
           From the CIF: _cell_formula_units_Z    4
           From the CIF: _chemical_formula_sum  C56.50 H50 Cl4 N4 O2 P2 Se2 Tc2
           TEST: Compare cell contents of formula and atom_site data

           atom    Z*formula  cif sites diff
           C        226.00    212.00   14.00
           H        200.00    184.00   16.00
           Cl        16.00     16.00    0.00
           N         16.00     16.00    0.00
           O          8.00      8.00    0.00
           P          8.00      8.00    0.00
           Se         8.00      8.00    0.00
           Tc         8.00      8.00    0.00
PLAT041_ALERT_1_G Calc. and Reported SumFormula    Strings  Differ     Please Check
              Calc: C53 H46 Cl4 N4 O2 P2 Se2 Tc2
              Rep.: C56.50 H50 Cl4 N4 O2 P2 Se2 Tc2
PLAT042_ALERT_1_G Calc. and Reported MoietyFormula Strings  Differ     Please Check
              Calc: C46 H38 Cl4 N4 O2 P2 Se2 Tc2, C7 H8
              Rep.: C46 H38 Cl4 N4 O2 P2 Se2 Tc2, C7 H8, 0.5[C7H8]
PLAT083_ALERT_2_G SHELXL Second Parameter in WGHT  Unusually Large      30.98 Why ?
PLAT232_ALERT_2_G Hirshfeld Test Diff (M-X)  Tc1      --N10      .        5.5 s.u.
PLAT605_ALERT_4_G Largest Solvent Accessible VOID in the Structure        303 A**3
PLAT868_ALERT_4_G ALERTS Due to the Use of _smtbx_masks Suppressed          ! Info
PLAT910_ALERT_3_G Missing # of FCF Reflection(s) Below Theta(Min).          4 Note
               1  0  0,   1  1  0,   0  2  0,   0  1  1,
PLAT912_ALERT_4_G Missing # of FCF Reflections Above STh/L=  0.600          7 Note
PLAT913_ALERT_3_G Missing # of Very Strong Reflections in FCF ....          1 Note
               0  2  0,
PLAT969_ALERT_5_G The 'Henn et al.' R-Factor-gap value ..........      3.547 Note
              Predicted wR2: Based on SigI**2  3.47 or SHELX Weight 10.48
```

PLAT978_ALERT_2_G Number C-C Bonds with Positive Residual Density. 0 Info

---

0 **ALERT level A** = Most likely a serious problem - resolve or explain
0 **ALERT level B** = A potentially serious problem, consider carefully
5 **ALERT level C** = Check. Ensure it is not caused by an omission or oversight
14 **ALERT level G** = General information/check it is not something unexpected

6 ALERT type 1 CIF construction/syntax error, inconsistent or missing data
5 ALERT type 2 Indicator that the structure model may be wrong or deficient
4 ALERT type 3 Indicator that the structure quality may be low
3 ALERT type 4 Improvement, methodology, query or suggestion
1 ALERT type 5 Informative message, check

---

# Datablock: tcnosncl2pua

---

Bond precision: C-C = 0.0040 A Wavelength=0.71073

| | | | |
|---|---|---|---|
| Cell: | a=11.5899(6) | b=15.0081(7) | c=15.6037(9) |
| | alpha=90 | beta=99.722(2) | gamma=90 |
| Temperature: | 100 K | | |

| | Calculated | Reported |
|---|---|---|
| Volume | 2675.2(2) | 2675.2(2) |
| Space group | P 21/n | P 1 21/n 1 |
| Hall group | -P 2yn | -P 2yn |
| Moiety formula | 2(C23 H19 Cl2 N2 O P S Tc), C3.50 H3.50, C3.50 H4.50 | C23 H19 Cl2 N2 O P S Tc, 0.5[C6H14] |
| Sum formula | C53 H46 Cl4 N4 O2 P2 S2 Tc2 | C26.50 H23 Cl2 N2 O P S Tc |
| Mr | 1236.62 | 617.40 |
| Dx,g cm-3 | 1.535 | 1.533 |
| Z | 2 | 4 |
| Mu (mm-1) | 0.898 | 0.898 |
| F000 | 1248.0 | 1248.0 |
| F000' | 1244.62 | |
| h,k,lmax | 14,18,19 | 14,18,19 |
| Nref | 5487 | 5474 |
| Tmin,Tmax | 0.824,0.906 | 0.676,0.745 |
| Tmin' | 0.737 | |

Correction method= # Reported T Limits: Tmin=0.676 Tmax=0.745
AbsCorr = MULTI-SCAN

Data completeness= 0.998 Theta(max)= 26.392

```
R(reflections)= 0.0317( 4787)                        wR2(reflections)=
                                                     0.0733( 5474)
S = 1.155                 Npar= 381
```

---

The following ALERTS were generated. Each ALERT has the format
**test-name_ALERT_alert-type_alert-level.**
Click on the hyperlinks for more details of the test.

---

## Alert level C

```
PLAT041_ALERT_1_C Calc. and Reported SumFormula    Strings  Differ     Please Check
              Calc: C53 H46 Cl4 N4 O2 P2 S2 Tc2
              Rep.: C26.50 H23 Cl2 N2 O P S Tc
PLAT042_ALERT_1_C Calc. and Reported MoietyFormula Strings  Differ     Please Check
              Calc: 2(C23 H19 Cl2 N2 O P S Tc), C3.50 H3.50, C3.50
                     H4.50
              Rep.: C23 H19 Cl2 N2 O P S Tc, 0.5[C6H14]
PLAT043_ALERT_1_C Calculated and Reported Mol. Weight Differ by ..       1.82 Check
PLAT234_ALERT_4_C Large Hirshfeld Difference N1       --C2B      .       0.16 Ang.
PLAT911_ALERT_3_C Missing FCF Refl Between Thmin & STh/L=    0.600          7 Report
                4  0  0,   0  2  0,   3  0  1,  -2  0  2,   0  0  2,   4  0  2,
               -1  1  2,
PLAT913_ALERT_3_C Missing # of Very Strong Reflections in FCF ....          7 Note
                4  0  0,   0  2  0,   3  0  1,  -2  0  2,   0  0  2,   4  0  2,
               -1  1  2,
PLAT977_ALERT_2_C Check Negative Difference Density on H42A      .      -0.41 eA-3
```

---

## Alert level G

```
FORMU01_ALERT_1_G  There is a discrepancy between the atom counts in the
            _chemical_formula_sum and _chemical_formula_moiety. This is
            usually due to the moiety formula being in the wrong format.
            Atom count from _chemical_formula_sum:   C26.5 H23 Cl2 N2 O1 P1 S1 Tc1
            Atom count from _chemical_formula_moiety:C26 H26 Cl2 N2 O1 P1 S1 Tc1
PLAT045_ALERT_1_G Calculated and Reported Z Differ by a Factor ...      0.500 Check
PLAT083_ALERT_2_G SHELXL Second Parameter in WGHT  Unusually Large       5.67 Why ?
PLAT171_ALERT_4_G The CIF-Embedded .res File Contains EADP Records          3 Report
PLAT232_ALERT_2_G Hirshfeld Test Diff (M-X)  Tc1      --Cl2A     .       14.8 s.u.
PLAT232_ALERT_2_G Hirshfeld Test Diff (M-X)  Tc1      --S1A      .       11.3 s.u.
PLAT232_ALERT_2_G Hirshfeld Test Diff (M-X)  Tc1      --N10      .        6.8 s.u.
PLAT299_ALERT_4_G Atom Site Occupancy Constrained at ............        0.5 Check
              C35A    C35B    C36A    C36B    H34     H34A    H35A    H35B
              H36A    H36B    C41     C43     C45     H41     H45     C42
              C44     C46     H42A    H42B    H46
PLAT300_ALERT_4_G Atom Site Occupancy of Cl2A       Constrained at       0.85 Check
PLAT300_ALERT_4_G Atom Site Occupancy of S1A        Constrained at       0.85 Check
PLAT300_ALERT_4_G Atom Site Occupancy of Cl2B       Constrained at       0.15 Check
PLAT300_ALERT_4_G Atom Site Occupancy of S1B        Constrained at       0.15 Check
PLAT300_ALERT_4_G Atom Site Occupancy of C2A        Constrained at       0.85 Check
PLAT300_ALERT_4_G Atom Site Occupancy of C3A        Constrained at       0.85 Check
PLAT300_ALERT_4_G Atom Site Occupancy of C4A        Constrained at       0.85 Check
PLAT300_ALERT_4_G Atom Site Occupancy of C5A        Constrained at       0.85 Check
PLAT300_ALERT_4_G Atom Site Occupancy of C6A        Constrained at       0.85 Check
PLAT300_ALERT_4_G Atom Site Occupancy of C2B        Constrained at       0.15 Check
PLAT300_ALERT_4_G Atom Site Occupancy of C3B        Constrained at       0.15 Check
```

```
PLAT300_ALERT_4_G Atom Site Occupancy of C4B        Constrained at       0.15 Check
PLAT300_ALERT_4_G Atom Site Occupancy of C5B        Constrained at       0.15 Check
PLAT300_ALERT_4_G Atom Site Occupancy of C6B        Constrained at       0.15 Check
PLAT300_ALERT_4_G Atom Site Occupancy of H3A        Constrained at       0.85 Check
PLAT300_ALERT_4_G Atom Site Occupancy of H4A        Constrained at       0.85 Check
PLAT300_ALERT_4_G Atom Site Occupancy of H5A        Constrained at       0.85 Check
PLAT300_ALERT_4_G Atom Site Occupancy of H6A        Constrained at       0.85 Check
PLAT300_ALERT_4_G Atom Site Occupancy of H3B        Constrained at       0.15 Check
PLAT300_ALERT_4_G Atom Site Occupancy of H4B        Constrained at       0.15 Check
PLAT300_ALERT_4_G Atom Site Occupancy of H5B        Constrained at       0.15 Check
PLAT300_ALERT_4_G Atom Site Occupancy of H6B        Constrained at       0.15 Check
PLAT300_ALERT_4_G Atom Site Occupancy of C48        Constrained at       0.25 Check
PLAT300_ALERT_4_G Atom Site Occupancy of H48A       Constrained at       0.25 Check
PLAT300_ALERT_4_G Atom Site Occupancy of H48B       Constrained at       0.25 Check
PLAT300_ALERT_4_G Atom Site Occupancy of H48C       Constrained at       0.25 Check
PLAT300_ALERT_4_G Atom Site Occupancy of C47        Constrained at       0.25 Check
PLAT300_ALERT_4_G Atom Site Occupancy of H47A       Constrained at       0.25 Check
PLAT300_ALERT_4_G Atom Site Occupancy of H47B       Constrained at       0.25 Check
PLAT300_ALERT_4_G Atom Site Occupancy of H47C       Constrained at       0.25 Check
PLAT301_ALERT_3_G Main Residue  Disorder ..............(Resd    1)        29% Note
PLAT302_ALERT_4_G Anion/Solvent/Minor-Residue Disorder (Resd    2)       100% Note
PLAT302_ALERT_4_G Anion/Solvent/Minor-Residue Disorder (Resd    3)       100% Note
PLAT432_ALERT_2_G Short Inter X...Y Contact  C34      ..C3B      .       3.19 Ang.
                                                   1-x,1-y,1-z  =      3_666 Check
PLAT811_ALERT_5_G No ADDSYM Analysis: Too Many Excluded Atoms ....          ! Info
PLAT910_ALERT_3_G Missing # of FCF Reflection(s) Below Theta(Min).          3 Note
                1  1  0,  -1  0  1,   0  1  1,
PLAT912_ALERT_4_G Missing # of FCF Reflections Above STh/L=  0.600          5 Note
PLAT969_ALERT_5_G The 'Henn et al.' R-Factor-gap value ..........       3.543 Note
              Predicted wR2: Based on SigI**2  2.07 or SHELX Weight  6.35
PLAT978_ALERT_2_G Number C-C Bonds with Positive Residual Density.          6 Info
```

---

0 **ALERT level A** = Most likely a serious problem - resolve or explain
0 **ALERT level B** = A potentially serious problem, consider carefully
7 **ALERT level C** = Check. Ensure it is not caused by an omission or oversight
47 **ALERT level G** = General information/check it is not something unexpected

5 ALERT type 1 CIF construction/syntax error, inconsistent or missing data
7 ALERT type 2 Indicator that the structure model may be wrong or deficient
4 ALERT type 3 Indicator that the structure quality may be low
36 ALERT type 4 Improvement, methodology, query or suggestion
2 ALERT type 5 Informative message, check

---

---

It is advisable to attempt to resolve as many as possible of the alerts in all categories. Often the minor alerts point to easily fixed oversights, errors and omissions in your CIF or refinement strategy, so attention to these fine details can be worthwhile. In order to resolve some of the more serious problems it may be necessary to carry out additional measurements or structure refinements. However, the purpose of your study may justify the reported deviations and the more serious of these should normally be commented upon in the discussion or experimental section of a paper or in the "special_details" fields of the CIF. checkCIF was carefully designed to identify outliers and unusual parameters, but every test has its limitations and alerts that are not important in a particular case may appear. Conversely, the absence of alerts does not guarantee there are no aspects of the results needing attention. It is up to the individual to critically assess their own results and, if necessary, seek expert advice.

**Publication of your CIF in IUCr journals**

A basic structural check has been run on your CIF. These basic checks will be run on all CIFs submitted for publication in IUCr journals (*Acta Crystallographica*, *Journal of Applied Crystallography*, *Journal of Synchrotron Radiation*); however, if you intend to submit to *Acta Crystallographica Section C* or *E* or *IUCrData*, you should make sure that full publication checks are run on the final version of your CIF prior to submission.

**Publication of your CIF in other journals**

Please refer to the *Notes for Authors* of the relevant journal for any special instructions relating to CIF submission.

---

**PLATON version of 19/12/2024; check.def file version of 19/12/2024**

**Datablock tcnoop2ua** - ellipsoid plot

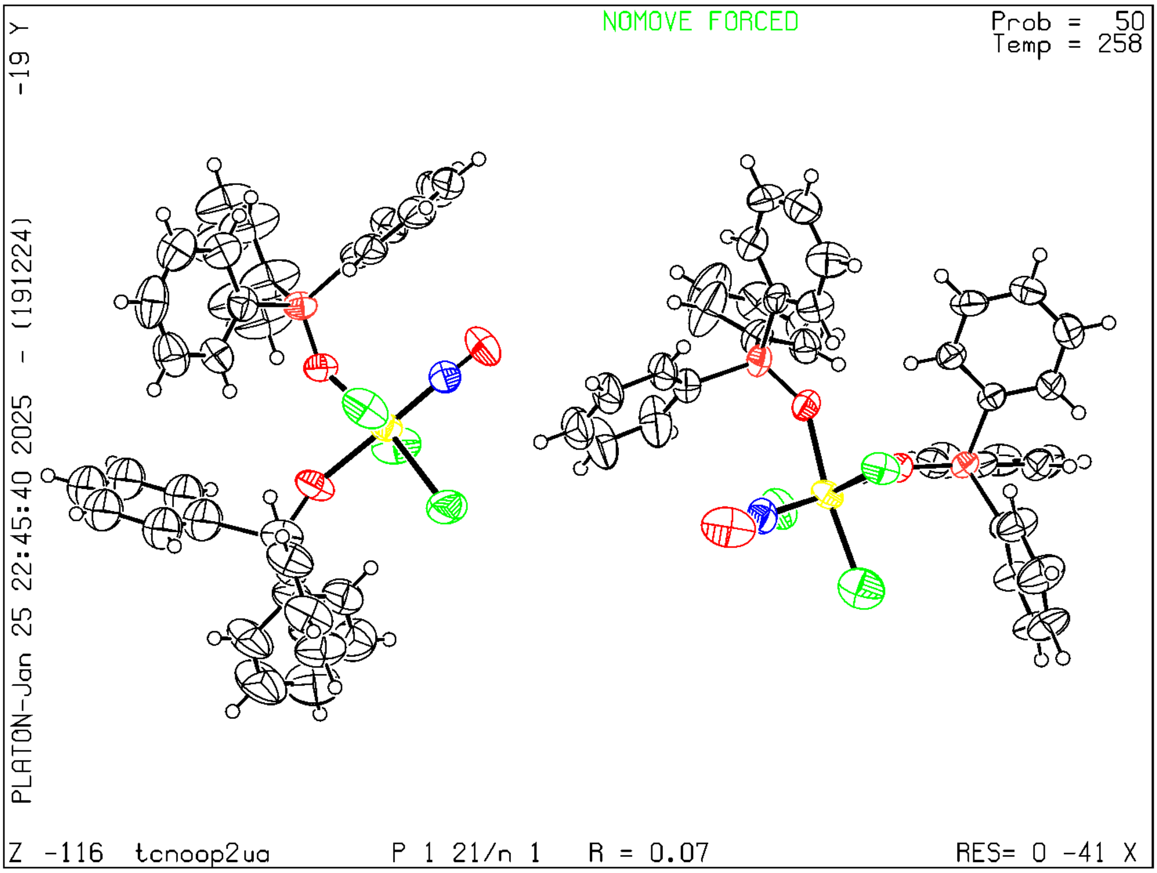

**Datablock tcnote2pyua** - ellipsoid plot

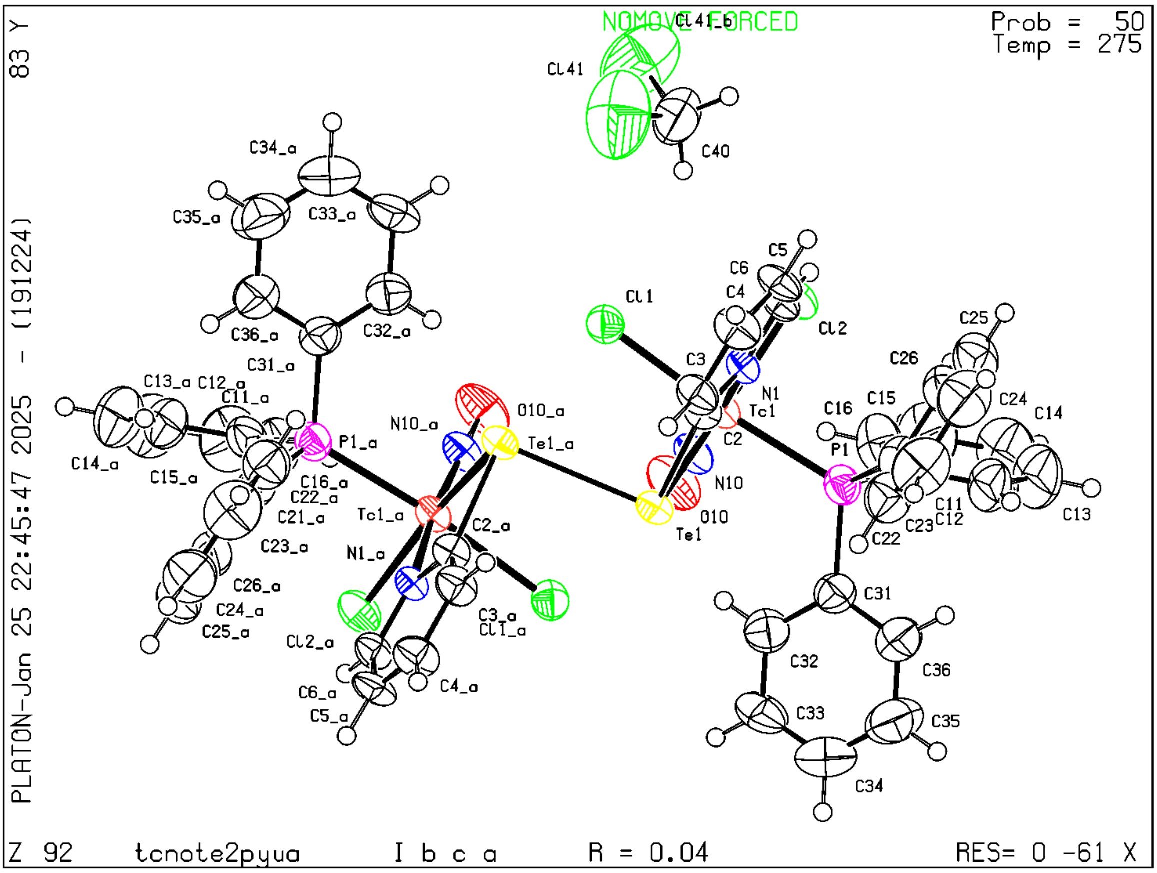

**Datablock tcnose2ua** - ellipsoid plot

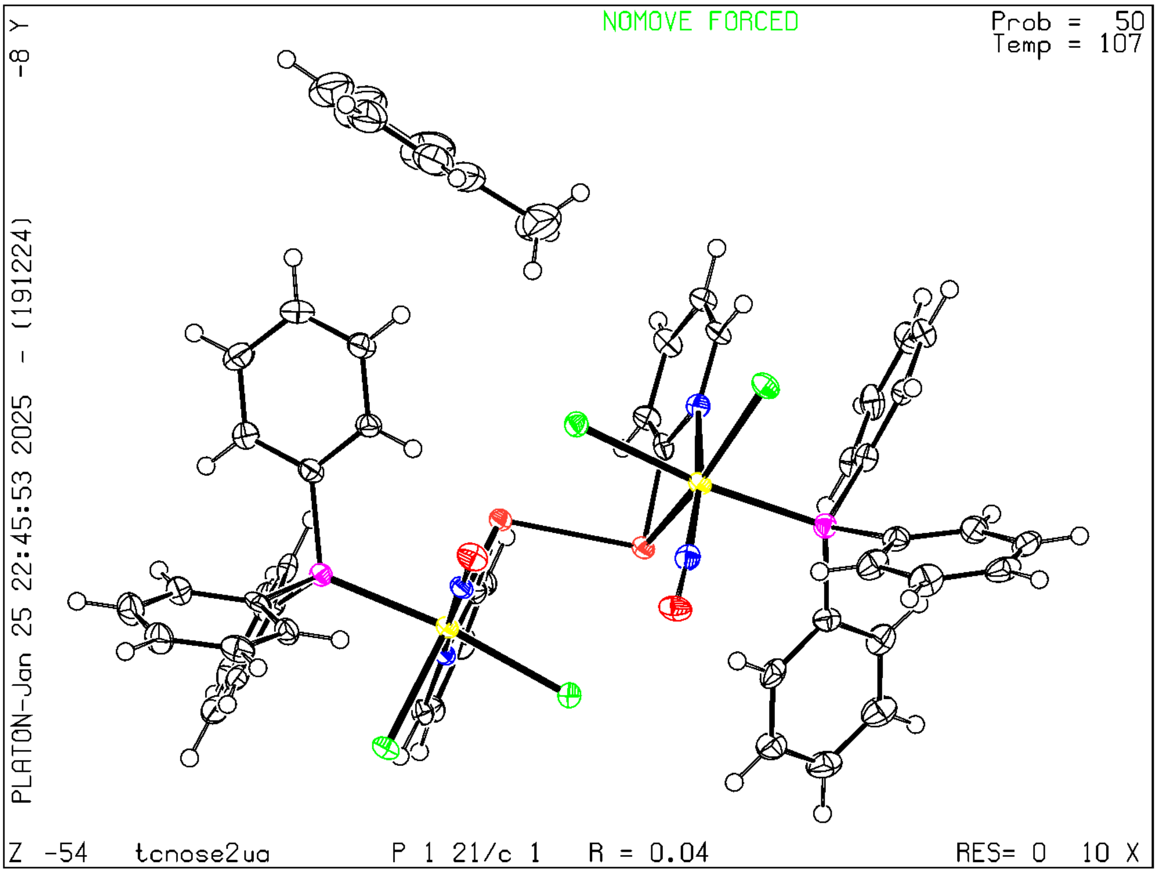

**Datablock tcnosncl2pua** - ellipsoid plot

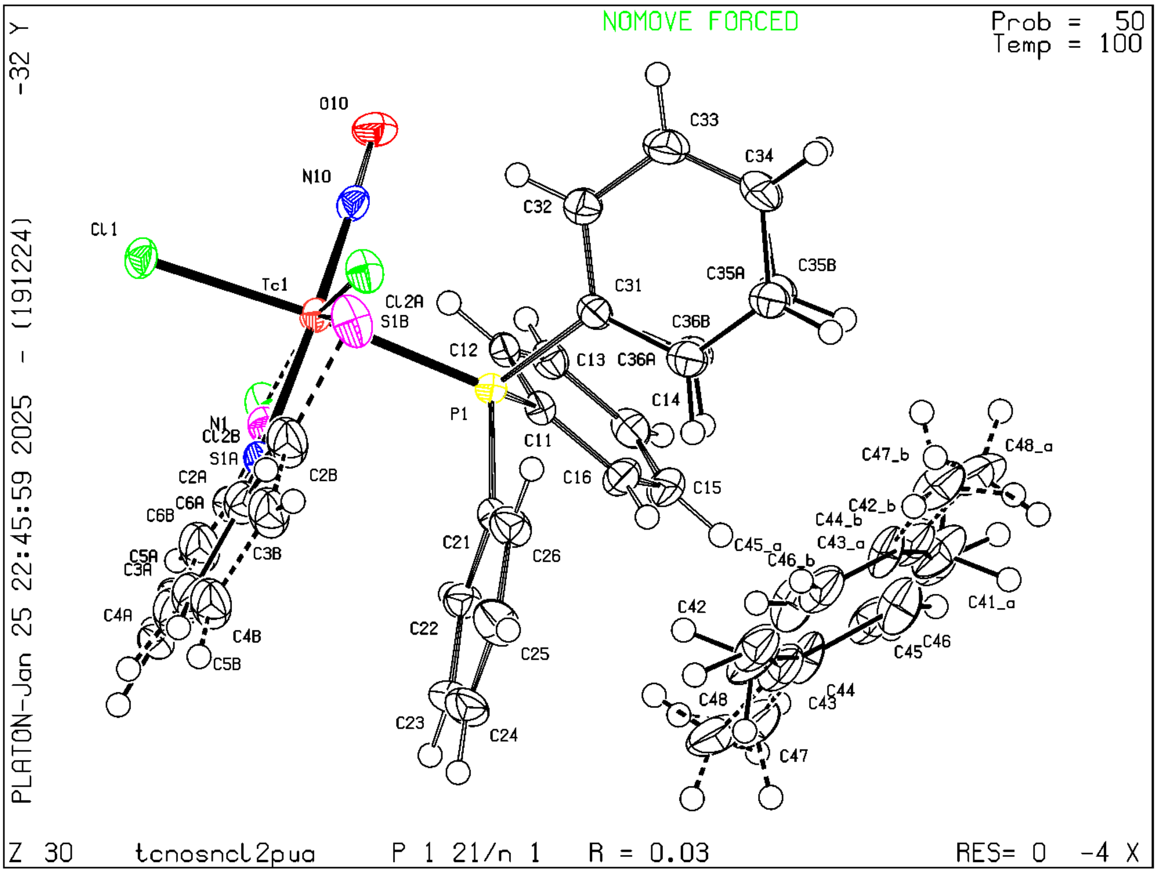

Supplement: Supplementary file 1 [file molecules-30-00793-s001.zip › molecules-3447112-supplementary/Supplement/Tc_NO_dichalcogenes_checkcif_rev.pdf]
